# Synthesis, Characterization, Molecular Docking Study and Biological Evaluation of Novel 1,3,4‐Thiadiazole‐Based Heterocycles as Potential Anticancer and Antioxidant Agents

**DOI:** 10.1111/cbdd.70361

**Published:** 2026-07-15

**Authors:** Nashwa M. El‐Metwaly, Abdulmajeed F. Alrefaei, Hossa F. Alshareef, Samar J. Almehmadi, Fatimah A. Alotaibi, Nadiyah M. Alshammari, Manal S. Ebaid, Hanadi A. Katouah

**Affiliations:** ^1^ Department of Chemistry College of Science, Umm Al‐Qura University Makkah Saudi Arabia; ^2^ Department of Biology/Genetic and Molecular Biology Central Laboratory (GMCL) Jamoum University College, Umm Al‐Qura University Makkah Saudi Arabia; ^3^ Department of Chemistry, Faculty of Science University of Tabuk Tabuk Saudi Arabia; ^4^ Department of Chemistry College of Science, Qassim University Buraidah Saudi Arabia; ^5^ Department of Chemistry College of Science, Northern Border University Arar Saudi Arabia

**Keywords:** 1,3,4‐Thiadiazole, anticancer activity, antioxidant activity, heterocyclic compounds, molecular docking

## Abstract

The design, synthesis, and biological assessment of new heterocyclic compounds containing the 1,3,4‐thiadiazole moiety, a well‐known pharmacophore with substantial medicinal significance, are the main topics of this study. To create a variety of thiadiazole‐based heterocyclic systems, such as pyrazoles, triazoles, pyrimidines, and thiophenes, a novel cyanoacetohydrazide derivative was created and used as a crucial intermediate. IR, ^1^H‐NMR, ^13^C‐NMR, and mass spectrometry were the spectroscopic methods used to confirm the structures of produced compounds. The synthesized compounds' biological properties were assessed for their potential as antioxidants and anticancer agents. Several derivatives showed impressive activity in cytotoxicity tests against HePG‐2 and MCF‐7 cancer cell lines, with several compounds showing greater potency than the reference medication 5‐fluorouracil. Compounds **17**, **18**, and **13** exhibited the strongest anticancer activity, highlighting the significance of structural and electronic properties in improving biological performance. Furthermore, an assessment of the synthesized compounds' antioxidant activity revealed that they have differing levels of free radical scavenging activity, with compounds **17** and **18** exhibiting the most notable benefits, albeit still less than ascorbic acid. Electron‐donating and electron‐withdrawing groups, molecular conjugation, and lipophilicity all play a part in regulating antioxidant and anticancer actions, according to structure–activity relationship (SAR) studies. Additionally, substantial interactions between specific drugs and the EGFR tyrosine kinase domain were suggested by molecular docking studies, indicating their possible mode of action. All things considered, our work shows that thiadiazole‐based heterocycles are attractive candidates for the creation of novel antioxidants and anticancer medicines and offers insightful information for future drug design.

## Graphical abstract

A new cyanoacetohydrazide derivative was developed as a key intermediate to synthesize diverse 1,3,4‐thiadiazole‐based heterocyclic compounds. The synthesized compounds were structurally confirmed. Several compounds demonstrated strong cytotoxic activity against HePG‐2 and MCF‐7 cancer cell lines, with compounds **17**, **18**, and **13** exceeding the activity of 5‐fluorouracil. The compounds exhibited dose‐dependent antioxidant activity, with compounds **17** and **18** showing the highest free radical scavenging effects. Biological activity was significantly influenced by electronic properties, lipophilicity, and molecular conjugation according to SAR analysis as well as the molecular docking against EGFR tyrosine kinase domain.

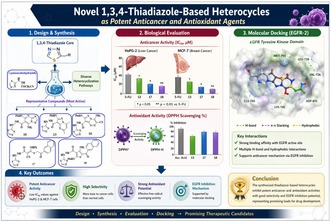

## 1 Introduction

Because of their diverse range of biological actions, heterocyclic compounds are among the most significant groups of organic molecules in medicinal chemistry. Among them, five‐membered heterocycles with sulfur and nitrogen atoms have garnered a lot of interest, especially the 1,3,4‐thiadiazole moiety, which is regarded as a preferred scaffold in drug discovery (Serban et al. 2019; Dawood and Farghaly 2017; Ghorai et al. 2020; Farzam and Abdullah 2021). A planar aromatic ring with two nitrogen atoms and one sulfur atom makes up the 1,3,4‐thiadiazole nucleus, which gives it special electronic properties, high stability, and the capacity to engage in hydrogen bonding and coordination with biological targets. Because of these characteristics, thiadiazole derivatives are extremely adaptable pharmacophores with a wide range of biological uses, such as antibacterial, anti‐inflammatory, anticonvulsant, antioxidant, and particularly anticancer properties (Serban et al. 2018; Can et al. 2018; Gür et al. 2020; Farooqi et al. 2018; Shivakumara and Krishna 2020).

**FIGURE 1:**
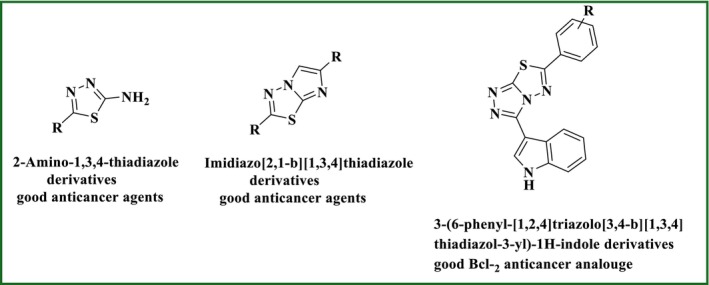
Examples of biologically active 1,3,4‐thiadiazole derivatives.

The development of thiadiazole‐based compounds as possible anticancer medicines has attracted increasing attention in recent years. These derivatives' capacity to interact with important cellular targets, including tubulin polymerization, protein kinases, and enzymes involved in DNA replication, is primarily responsible for their anticancer effect (Obakachi et al. 2021; Ismail et al. 2021; Serban 2019). Through mechanisms such as cell cycle arrest, apoptosis induction, and angiogenesis suppression, several thiadiazole derivatives have shown lethal effects against a variety of cancer cell lines (Figure 1). Because of their structural flexibility, pharmacological characteristics and target selectivity can be optimized with ease (Serban et al. 2018; Serban 2019; Suryawanshi et al. 2021; Zurawska et al. 2021; Horvath et al. 2014; Cascioferro et al. 2020; Mannam et al. 2019; Anthwal et al. 2022).

Epidermal growth factor receptor (EGFR) is a well‐established receptor tyrosine kinase involved in the regulation of cell proliferation, survival, and tumor progression. Aberrant EGFR expression has been reported in several cancers, including breast and hepatocellular carcinomas. Therefore, EGFR was selected as a relevant molecular target for docking studies to explore the possible binding mode of the synthesized compounds and provide a preliminary explanation for their observed cytotoxic activity.

1,3,4‐Thiadiazole compounds have demonstrated exceptional antioxidant action in addition to their potential as anticancer agents. The development of many diseases, including cancer, is significantly influenced by oxidative stress, which is brought on by an imbalance between reactive oxygen species (ROS) and antioxidant defenses (Djukic et al. 2008, 2012; Halyna 2014; Kagan et al. 2000; Grozav et al. 2017). By scavenging free radicals, chelating metal ions, and preventing lipid peroxidation, thiadiazole compounds function as potent antioxidants. Their capacity to donate electrons is improved by the presence of heteroatoms (N and S) and conjugated systems, which is crucial for neutralizing free radicals. In order to increase biological activity, recent synthetic approaches have concentrated on adding the thiadiazole ring to more intricate heterocyclic structures. The thiadiazole core is a crucial component for generating a variety of heterocyclic frameworks, including pyrazoles, oxadiazoles, triazoles, and thiophenes, as this work has shown. Because several bioactive moieties work in concert, these hybrid systems frequently show better pharmacological characteristics.

Oxidative stress has been recognized as an important factor in the initiation and progression of cancer through its effects on DNA damage, cellular signaling, and tumor development. Consequently, compounds possessing antioxidant properties may contribute to protection against oxidative damage and have attracted considerable interest in medicinal chemistry. Since many thiadiazole‐containing derivatives have been reported to exhibit both antioxidant and anticancer activities, evaluation of the antioxidant potential of the synthesized compounds was considered relevant in order to provide a broader assessment of their biological properties.

Furthermore, thiadiazole derivatives' plasticity in chemical transformations makes it possible to create new molecules with improved biological potential. The chemical space for drug development is greatly increased by the capacity to go through cyclization reactions and create fused heterocycles. This strategy is in line with contemporary medicinal chemistry techniques that attempt to create multifunctional compounds that can target several biological processes. In conclusion, a potential class of molecules in the hunt for novel antioxidants and anticancer medicines is heterocyclic compounds with the 1,3,4‐thiadiazole moiety. They are appealing prospects for additional pharmaceutical research due to their distinctive structural characteristics, wide range of biological activity, and synthetic accessibility.

## 2 Experiment

### 2.1 Synthesis and Procedure

Detailed synthetic procedures and characterization data are provided in the Supporting Information.

### 2.2 Biological Activity

#### 2.2.1 Antitumor Evaluation

It was carried out according to the previously reported work (Fadda et al. 2015).

#### 2.2.2 Antioxidant Activity

The antioxidant activity was assessed in vitro utilizing 1,1‐diphenyl‐2‐picrylhydrazyl (DPPH) radical scavenging activity. To do this, various quantities of the produced compounds were combined with a solution of DPPH in ethanol (0.1 mM). A spectrophotometer was used to measure the absorbance change at 515 nm after the solution was mixed at room temperature for 10 min. Ascorbic acid's ability to scavenge radicals was used as a control to compare the synthetic compounds' activity (Covell et al. 2007; Madhuri and Pandey 2009).

#### 2.2.3 Molecular Docking Study

As the five‐dimensional structures of the synthesized compounds **10**, **13**, **16**, **17**, **18**, and the 5‐fluorouracil were constructed using the M.O.E. program, energy minimization was achieved using the MMFF94x force field till a gradient of 0.01 kcal/mol/Å was performed. EGFR was selected as the molecular docking target because of its well‐established role in cancer cell proliferation, survival, and tumor progression. Furthermore, EGFR dysregulation has been reported in both breast and hepatocellular carcinomas, the cancer types represented by the MCF‐7 and HepG‐2 cell lines used in the present study. Therefore, molecular docking against the EGFR tyrosine kinase domain was performed to explore the potential binding interactions of the synthesized compounds and to provide a preliminary mechanistic interpretation of their observed cytotoxic activity. The crystal structure of the EGFR tyrosine kinase domain (PDB ID: 1M17) was retrieved from the Protein Data Bank (PDB) (Hussein et al. 2025). The protein was protonated, and both hydrogen atoms and water molecules were removed. Docking replications were performed using the Triangle Matcher placement technique and the London dG scoring function, followed by refinement with the GBVI/WSA dG scoring function. The binding sites were defined based on the co‐crystallized ligand. For each compound, **10** docking poses were generated, and the best pose was selected based on the lowest binding energy (S, kcal/mol) and RMSD values less than two. In addition, Ligand‐protein interactions (H‐bonds, pi‐H stacking, and hydrophobic interactions) were analyzed using the M.O.E.

## 3 Results and Discussion

### 3.1 The Characterization of Newly Synthesized Compounds

**SCHEME 1:**
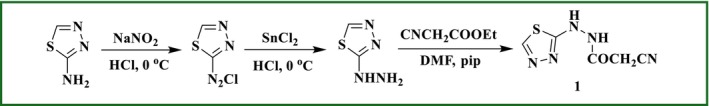
Synthesis of cyanoacetohydrazide **1**.

According to our knowledge, cyanoacetohydrazide **1** has not been previously published or prepared. Its preparation in this work is the first of its kind and represents a novel synthetic approach, as it was used as a starting material for the preparation of several heterocyclic compounds linked to the thiadiazol ring to study their biological activity, which is also novel. Compound **1** was prepared starting from 2‐amino‐thiadiazole. The diazonium salt of this compound was prepared and reduced to obtain 2‐hydrazino‐1,3,4‐thiadiazole, which in turn reacted with ethyl cyanoacetate in the presence of boiling DMF and piperidine drops as a catalyst (Scheme 1).

**SCHEME 2:**
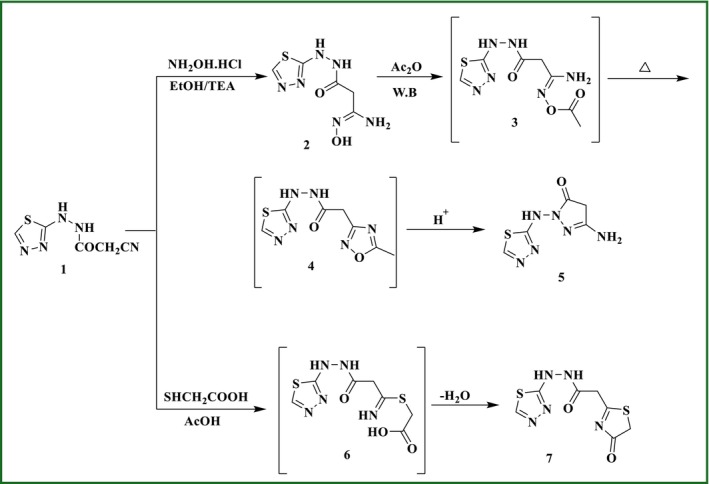
Synthesis of aminopyrazolone and thiazolone derivatives **5** and **7**, respectively.

Thus, precursor **1** was treated with hydroxylamine in the presence of TEA as a base to synthesize amidoxime **2**. Acetic anhydride was used to acetylate amidoxime **2**, resulting in *O*‐acetylation. Pyrazolin‐5‐one derivative **5** was obtained by acid catalysis after compound **3** was thermally cyclized to produce 1,2,4‐oxadiazole derivative **4** (Scheme 2). The amidoxime **2** was distinguished by the lack of nitrile function in its infrared spectrum, the appearance of a hydroxyl group at absorption band 3460 cm^−1^, and amino and imino function at absorption bands 3420, 3230, and 3210 cm^−1^. The presence of a singlet due to two protons at *δ* 3.19 ppm in the ^1^H‐NMR spectrum, which corresponds to the methylene protons of the pyrazolone moiety, allowed for the identification of the structure of **5**. Its infrared spectra also showed stretching frequencies of 3430, 3310, and 1682 cm^−1^, which were identified as NH_2_, NH, amidic, and CO, respectively.

By cyclocondensing cyanoacetohydrazide **1** with 2‐mercapto acetic acid in boiling glacial acetic acid, the thiazolinone derivative **7** was obtained in significant amounts. The infrared spectrum of thiazolinone derivative **7** revealed stretching frequencies at 3220 cm^−1^, which were linked to 2NH groups, and a notable absorption band at 1710 and 1682 cm^−1^, which were linked to 2CO functions. Its ^1^H‐NMR spectra revealed a singlet signal at *δ* 2.95 ppm, which was assigned to methylene protons, and a singlet signal at *δ* 4.20 ppm, which was attributed to two protons of the thiazolinone moiety's methylene protons.

**SCHEME 3:**
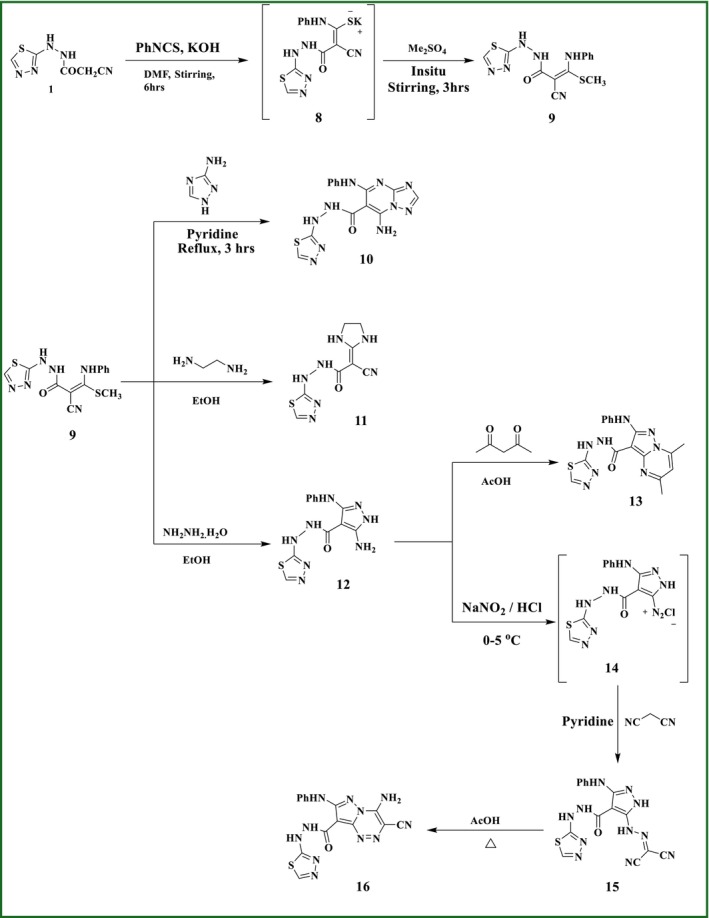
Reaction of thiocarbamoyl **9** with *N*‐nucleophiles.

When **1** interacted with phenyl isothiocyanate in DMF with potassium hydroxide at room temperature, the non‐isolable intermediate thiocarbamoyl salt (**8**) was obtained. The intermediate thiocarbamoyl salt **8** was in situ alkylated with dimethyl sulfate to create acrylamide **9** (Scheme 3). The presence of the ketene *N*,*S*‐acetal **9** was verified by spectral and elemental analyses. S‐CH_3_ protons were identified as the source of a singlet signal at *δ* 2.30 ppm in its ^1^H‐NMR spectra. The reactivity of acrylamide **9** with nitrogen nucleophiles was investigated. After that, compound **9** was heated with 3‐amino‐1H‐1,2,4‐triazole in pyridine to produce triazolo[1,5‐a]pyrimidine derivative **10**. The reaction process may be assumed to involve the Michael addition of the amino group to the ethylenic bond in **9**, followed by the elimination of methanethiol. After that, **10** was produced by nucleophilically adding the nitrogen atom to the nitrile carbon (Scheme 3). Structure **10** was validated by its spectral observations. Consequently, its infrared spectra revealed no conjugated cyano function absorption band and absorption bands at 3425, 3315, and 1681 cm^−1^ that were ascribed to NH_2_, 3NH, and amidic CO functions, respectively.

Compound **9** was treated with bifunctional nucleophilic reagents, such as ethylenediamine in boiling ethanol, to produce the imidazolidine derivative **11** (Scheme 3). The IR spectra of **11** showed a stretching broad band at 3235 cm^−1^ assigned to four NH groups and an absorption band at 2220 cm^−1^ associated with the cyano function, in addition to a strong absorption band at 1683 cm^−1^ attributed to the carbonyl function. The signal at *δ* 3.75 ppm in its ^1^H‐NMR spectrum, or four protons, was caused by the two methylene protons. After heating under reflux, acrylamide **9** was cyclocondensed with hydrazine hydrate in EtOH to obtain the necessary 5‐aminopyrazole derivatives **12**. The IR spectra of **12** revealed that the absorption band at 1673 cm^−1^ is caused by the amidic (CO) function group, the absorption broad band at 3310 cm^−1^ is caused by the (4NH) groups, and the absorption band at 3445 cm^−1^ is caused by the (NH_2_) function. ^1^H‐NMR identified NH_2_ protons as a singlet signal comparable to two protons at *δ* 6.40 ppm.

5‐Aminopyrazoles have been used extensively as a flexible precursor for the synthesis of several heterocycles and as an essential intermediary for the synthesis of numerous polyfunctionalized fused pyrazoles with anticipated biological reactions. Thus, the cyclocondensation reaction of aminopyrazole **12** with acetylacetone in glacial acetic acid under reflux yielded pyrazolo[1,5‐a]pyrimidine derivative **13** (Scheme 3). The reported structure **13** was compatible with its IR, NMR, and elemental studies, with a parent ion peak (M^+^) at m/z 380, assigned to the molecular formula C_17_H_16_N_8_OS. Furthermore, compound **12** was diazotized using strong HCl and sodium nitrite to create the corresponding diazonium chloride **14**. The desired hydrazono derivatives **15** were then obtained by the reaction of thin with malononitrile in pyridine. Compound **15** was heated in glacial acetic acid to produce pyrazolo[5,1‐c][1,2,4]triazine derivatives **16** (Scheme 3). A carbonyl function at 1675 cm^−1^, two cyano functions at 2220 and 2219 cm^−1^, huge absorption bands at 3315 cm^−1^ attributed to (5NH), and a C=N peak at 1622 cm^−1^ were all visible in the infrared spectra of **15**.

The synthesis of thiazole, pyrazole, oxazine, and pyrimidine ring systems has been made easier by investigating the reaction between phenyl isothiocyanate and active methylene compounds under alkaline circumstances. Using phenyl isothiocyanate as a crucial starting material, our present synthetic efforts have expanded to include the synthesis of heterocyclic ring systems that were previously unattainable. After interacting with α‐halocarbonyl compounds, a variety of substrates with the N=C=S moiety undergo cyclization, producing thiazoles, 2,3‐dihydrothiazoles, and thiazolidines, which have demonstrated fungicidal, antiprotozoal, and local anesthetic effects (Pandya 2019).

**SCHEME 4:**
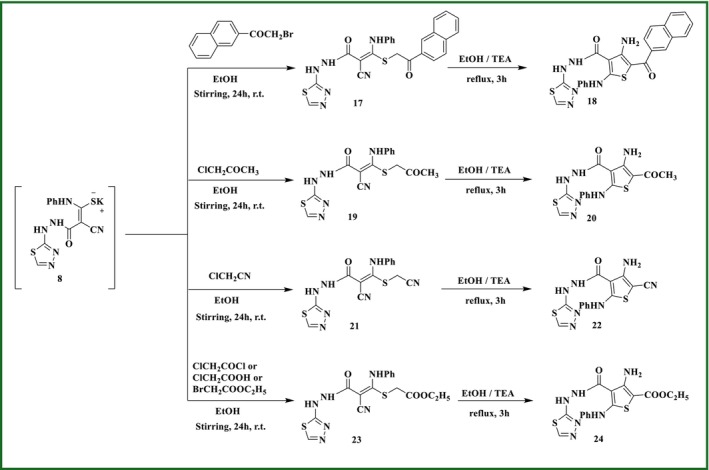
Reaction of thiocarbamoyl potassium salt **8** with α‐haloketone.

We offer a broadly applicable extension of the synthetic methodology first presented by Hantzsch and Weber (Hantzsch and Weber 1887). This enhanced method produces a yellow‐colored product **17** by stirring at room temperature compound **8** with equimolar quantities of 2‐(naphthalen‐2‐yl) acetyl bromide in boiling ethanol. To create thiophene derivative **18**, compound **17** was controversially heated for 4 h in ethanol with a catalytic quantity of TEA or refluxing DMF/TEA. Structures **17** and **18** were established using accurate spectral and elemental studies. Compound **17's** infrared spectrum revealed absorption spectra at 2220 cm^−1^, which corresponded to the nitrile group. In contrast, compound **18's** infrared spectrum showed a stretching frequency band at 3410 cm^−1^, which corresponded to the NH_2_ function, rather than an absorption band in the region at 2220 cm^−1^ due to the CN group. The chemical formula C_24_H_18_N_6_O_2_S_2_ (M^+^, 486.5) was revealed by the mass spectrum of **18** (Scheme 4).

Intermediate **8** readily interacted with chloroacetone in ethanol at room temperature to create the acyclic **19** by removing KCl. By refluxing **19** in ethanol with a catalytic quantity of TEA, the thiophene derivative **20** was created; its structure was confirmed by spectroscopic data and basic analysis. Its structure was further verified by a different synthesis of **20**. Therefore, by refluxing intermediate **8** directly with chloroacetone in DMF/TEA, the thiophene derivative **20** can be produced in a respectably high yield (Scheme 4).

Furthermore, the corresponding acyclic **21** is clearly and completely separated from stirring the intermediate **8** in ethanol with chloroacetonitrile. The structure of **21** has been verified by spectroscopic and elemental investigations. For instance, the infrared spectrum has bands at 3315 (NH), 2220, and 2218 cm^−1^ (2CN). Its ^1^H‐NMR shows a CH_2_ signal at *δ* 4.31 ppm. Furthermore, the thiophene derivative **22** is produced by heating **21** in ethanol with a catalytic amount of TEA. The structure was established using the IR spectra of thiophene structure **22**, which verified bands linked to NH_2_, NH, and CN functions. Its ^1^H‐NMR spectra show distinctive broad bands at *δ* 5.34 and 10.68 ppm caused by NH_2_ and NH. Nevertheless, it has been shown that it is directly formed by refluxing **8** and chloroacetonitrile in DMF/TEA (Scheme 4).

A favorable yield of a product with the formula was obtained by treating intermediate **8** with equimolar amounts of either chloroacetic acid or chloroacetyl chloride in ethanol. The identification of the acyclic structure **23** was made easier by the bands in its infrared spectra that corresponded to the NH, CN, and CO functionalities. Characteristic signals for CH_3_CH_2_, CH_2_, and CH_2_CH_3_ were detected in the ^1^H‐NMR spectrum at *δ* 1.49, 3.89, and 4.30 ppm, respectively. NH signal, interchangeable with D_2_O, at *δ* 10.29 ppm. On the other hand, when **8** was treated with ethyl bromoacetate in ethanol, a single product with the same melting point, mixed melting point, and infrared spectrum as **23** was formed. Compound **23's** mass spectrum (M^+^, 404) was shown. The equivalent thiophene derivative **24** was obtained by refluxing **23** in ethanol with a catalytic quantity of TEA (Scheme 4).

### 3.2 Biological Activity

#### 3.2.1 Anticancer Activity

HePG‐2 and MCF‐7 cancer cell lines were used to test the synthesized compounds' cytotoxic activities, and the results showed a distinct difference in potency between the tested derivatives. Compound **17** had IC_50_ values of 2.7 μM against HePG‐2 and 3.4 μM against MCF‐7, indicating that it was the most effective contender. Compared to the reference medication 5‐fluorouracil (5‐FU), which had IC_50_ values of 7.5 μM and 12 μM, respectively, its activity is substantially higher. Strong cytotoxic activity was also demonstrated by compounds **18** and **13**, which had IC_50_ values of 3.1 and 4.3 μM for HePG‐2 and 3.8 and 5.3 μM for MCF‐7, respectively. With IC_50_ values of 5.1 and 5.8 μM (HePG‐2) and 5.8 and 6.6 μM (MCF‐7), respectively, compounds **10** and **16** likewise showed significant potency, suggesting that they are part of the most active group.

Derivatives like **15**, **9**, and **21** showed IC_50_ values ranging from 6.3 to 9.7 μM against HePG‐2 and 7.0 to 9.6 μM against MCF‐7, making them moderately active compounds. Furthermore, compounds **23**, **24**, and **20** had moderate activity, with IC_50_ values of 12.8, 11.9, and 13.8 μM for HePG‐2 and 13.7, 12.8, and 14.6 μM for MCF‐7, respectively. Compounds **12**, **11**, and **7** showed somewhat less activity, with IC_50_ values of 13.2, 17.7, and 19.6 μM against HePG‐2 and 14.1, 18.5, and 20 μM against MCF‐7, respectively. Compounds **5**, **2**, and **1**, on the other hand, showed the least amount of cytotoxicity, with IC_50_ values of 27.6, 31, and 40 μM (HePG‐2) and 29.7, 36, and 46 μM (MCF‐7), respectively, suggesting minimal biological activity.

The most effective drugs maintained comparatively higher IC_50_ values against normal cells as compared to cancer cell lines (VERO and WI‐38), indicating some degree of selectivity. Compound **17**, for instance, demonstrated IC_50_ values of 16 μM (VERO) and 20 μM (WI‐38), in contrast to its significantly lower values against cancer cells (2.7 and 3.4 μM). In a similar vein, compound **18** had IC_50_ values of 18 and 22 μM against normal cells, but compounds **13** and **10** demonstrated IC_50_ values of 26–30 μM and 27–33 μM, respectively, suggesting a favorable safety margin. Conversely, less active compounds (e.g., compound **1**: 128 μM (VERO) and 142 μM (WI‐38)) showed high IC_50_ values in both cancer and normal cells, indicating poor overall cytotoxicity rather than genuine selectivity.

The structure–activity link can be used to explain the observed variations in activity. Compounds **17**, **18**, and **13** have superior activity, which may indicate the existence of structural elements that improve interaction with biological targets, such as electron‐withdrawing groups that boost electrophilicity and binding affinity. Furthermore, compounds **10** and **16** have relatively low IC_50_ values, suggesting that the ideal ratio of lipophilicity to molecular size may be essential for promoting cellular uptake and target engagement. Compounds **1**, **2**, and **5**, on the other hand, may have low activity due to steric hindrance or a lack of advantageous electronic properties, which may restrict their capacity to enter cells or bind to important biomolecules.

Overall, the findings unequivocally show that a number of the produced compounds, especially compound **17**, have better anticancer activity than 5‐FU, followed by compounds **18**, **13**, **10**, and **16**. The gradual decline in activity from these extremely powerful derivatives to weakly active substances emphasizes how crucial structural optimization is for boosting cytotoxic effects. These results bolster the series' promise as effective anticancer drugs and call for additional research to clarify their modes of action and enhance their pharmacological characteristics (Table 1).

**TABLE 1:** Cytotoxic activity (IC_50_, μM) of the synthesized compounds against HepG‐2, MCF‐7, WI‐38, and Vero cell lines.

Compound	HePG‐2	MCF‐7	VERO	WI‐38
**1**	40	46	128	142
**2**	31	36	112	120
**5**	27.6	29.7	92	101
**7**	19.6	20	66	73
**9**	8.3	8.9	32	37
**10**	5.1	5.8	27	33
**11**	17.7	18.5	63	70
**12**	13.2	14.1	55	60
**13**	4.3	5.3	26	30
**15**	6.3	7	25	31
**16**	5.8	6.6	24	29
**17**	2.7	3.4	16	20
**18**	3.1	3.8	18	22
**19**	9	9.8	34	40
**20**	13.8	14.6	49	54
**21**	9.7	9.6	35	41
**22**	14.7	15.7	51	59
**23**	12.8	13.7	47	56
**24**	11.9	12.8	45	52
**5‐Fu**	7.5	12	75	90

#### 3.2.2 Structure Activity Relationship

The synthetic compounds' structural characteristics and cytotoxic activity against the HePG‐2 and MCF‐7 cell lines were well correlated, according to the structure–activity relationship (SAR) study. Compounds can be classified into highly active, moderately active, and weakly active groups based on the measured IC_50_ values, which sheds light on the essential structural prerequisites for anticancer action. The compounds with the highest activity, especially compounds **17** (IC_50_ = 2.7 and 3.4 μM) and **18** (IC_50_ = 3.1 and 3.8 μM), indicate that strong electron‐withdrawing substituents are essential for increasing cytotoxic activity. These substituents probably make the molecules more electrophilic, which makes it easier for them to interact with biological targets like DNA or the active sites of enzymes. Compound **13** (IC_50_ = 4.3 and 5.3 μM) likewise showed great potency, demonstrating the significance of electronic properties in enhancing biological activity.

Lipophilicity seems to have a major impact on activity in addition to electrical variables. An ideal balance between hydrophilic and lipophilic characteristics improves cell membrane permeability and intracellular accumulation, as demonstrated by compounds like **10** and **16**, which have IC_50_ values of 5.1 and 5.8 μM (HePG‐2) and 5.8 and 6.6 μM (MCF‐7), respectively. These substances are probably able to effectively reach and interact with intracellular targets because of this balance.

Compounds with moderate activity, such as **15** (6.3 and 7 μM), **9** (8.3 and 8.9 μM), and **21** (9.7 and 9.6 μM), indicate that intermediate activity is caused by partial retention of advantageous structural characteristics. In contrast to the most powerful derivatives, small changes in substituent type or position may lower binding affinity or cellular absorption. In a similar vein, substances like **23**, **24**, and **20**, whose IC_50_ values range from 11.9 to 13.8 μM (HePG‐2), show that decreased activity is caused by weakened electronic properties or poor lipophilicity.

Conversely, compounds **12**, **11**, and **7** may have less favorable structural alignment or less interaction with biological targets, as evidenced by their IC_50_ values of 13.2–19.6 μM (HePG‐2) and 14.1–20 μM (MCF‐7). Compounds **5** (27.6 μM), **2** (31 μM), and **1** (40 μM) showed a further decrease in activity, indicating that steric hindrance or the absence of electron‐withdrawing groups considerably lowers cytotoxic potential. These substances may have more molecular bulk or poor planarity, which can make it more difficult for them to attach to DNA or enzyme active sites.

Additionally, the selectivity profile corroborates the SAR results because highly active compounds like **17** and **18** demonstrated selective toxicity toward cancer cells, as seen by their comparatively higher IC_50_ values versus normal cells (16–22 μM). This selectivity could result from preferred interaction with cancer‐specific molecular targets or increased absorption in quickly proliferating cancer cells. Overall, the SAR analysis shows that lipophilicity, steric parameters, and electronic properties have a significant impact on cytotoxic action in this series. Compounds **17**, **18**, and **13** show that obtaining high anticancer activity appears to need the presence of electron‐withdrawing substituents, ideal molecular size, and balanced polarity. These results offer important direction for future structural modification and the creation of stronger anticancer drugs.

#### 3.2.3 Antioxidant Activity

IC_50_ values and percentage inhibition at different concentrations (2, 5, and 10 μg/mL) were used to evaluate the antioxidant activity of the synthesized compounds. The results revealed considerable variation in antioxidant potency among the tested compounds, with inhibition percentages increasing progressively as the concentration increased, indicating a clear dose‐dependent response.

Among the investigated compounds, compound **17** exhibited the highest antioxidant activity, with an IC_50_ value of 2.3 μg/mL and inhibition percentages of 40%, 66%, and 89% at concentrations of 2, 5, and 10 μg/mL, respectively. Similarly, compound **18** demonstrated remarkable antioxidant activity, showing inhibition values of 38%, 64%, and 87%, with an IC_50_ value of 2.5 μg/mL. Compound **13** also displayed strong antioxidant potential, with an IC_50_ value of 3.3 μg/mL and a maximum inhibition of 83% at 10 μg/mL. Although these compounds were the most active antioxidants within the synthesized series, their activity remained lower than that of the reference standard ascorbic acid (Vit. C), which exhibited 96% inhibition at 10 μg/mL and an IC_50_ value of 0.8 μg/mL.

Compounds **10** and **12** also showed pronounced antioxidant activity, with IC_50_ values of 3.8 and 4.3 μg/mL, respectively. Compound **10** displayed inhibition percentages of 30%, 55%, and 80%, while compound **12** achieved inhibition values of 28%, 53%, and 78% at the corresponding concentrations. Furthermore, compounds **16** and **15** demonstrated comparable antioxidant activity, with IC_50_ values of 4.6 and 5.0 μg/mL and maximum inhibition percentages of 76% and 73%, respectively. These compounds can therefore be considered moderately to highly active antioxidants.

Moderate antioxidant activity was observed for compounds **9**, **19**, and **21**, which exhibited IC_50_ values of 5.8, 6.3, and 6.8 μg/mL, respectively, and inhibition percentages ranging from 63% to 68% at 10 μg/mL. Compounds **20**, **22**, **23**, and **24** showed comparatively lower activity, with IC_50_ values ranging from 7.8 to 9.3 μg/mL and maximum inhibition percentages between 51% and 58%.

In contrast, compounds **11**, **7**, **5**, **2**, and **1** displayed the weakest antioxidant activity. Compound **1** exhibited the highest IC_50_ value (22.0 μg/mL) and produced only 23% inhibition at 10 μg/mL, followed by compound **2** (21.0 μg/mL, 28% inhibition) and compound **5** (16.9 μg/mL, 33% inhibition). These findings indicate a relatively poor radical‐scavenging capacity for these derivatives.

From a structure–activity relationship perspective, the superior antioxidant activity of compounds **17**, **18**, and **13** may be attributed to the presence of structural features capable of efficiently donating hydrogen atoms or electrons, thereby enhancing their radical‐scavenging ability. In addition, conjugated systems and electron‐donating substituents may contribute to stabilization of the resulting radical species after hydrogen transfer. The relatively high activity observed for compounds **10**, **12**, **15**, and **16** further suggests that an appropriate balance of molecular architecture and electronic properties plays an important role in determining antioxidant efficiency.

Conversely, the lower antioxidant activity exhibited by compounds **1**, **2**, and **5** may be associated with the absence of efficient electron‐donating groups and/or insufficient stabilization of radical intermediates. Steric factors may also contribute by limiting the accessibility of reactive sites involved in free‐radical scavenging.

Overall, hydrogen‐donating ability, molecular conjugation, and electronic effects appear to significantly influence the antioxidant activity of this series. The observed activity order (**17** > **18** > **13** > **10** > **12** > **16** > **15** > **9** > **19** > **21** > others) highlights the importance of these structural features in enhancing radical‐scavenging efficiency and provides useful guidance for the future design of more potent antioxidant agents within this class of compounds (Table 2).

**TABLE 2:** IC_50_ values (μg/mL) and percentage inhibition of the antioxidant activity of the investigated compounds.

Compound no.	IC_50_ (μg/mL)	% Inhibition (2 μg/mL)	% Inhibition (5 μg/mL)	% Inhibition (10 μg/mL)
**1**	22.0	1	10	23
**2**	21.0	2	13	28
**5**	16.9	3	16	33
**7**	12.3	6	23	43
**9**	5.8	20	44	68
**10**	3.8	30	55	80
**11**	10.8	8	26	46
**12**	4.3	28	53	78
**13**	3.3	33	58	83
**15**	5.0	24	48	73
**16**	4.6	26	51	76
**17**	2.3	40	66	89
**18**	2.5	38	64	87
**19**	6.3	18	42	66
**20**	8.3	12	34	56
**21**	6.8	16	40	63
**22**	7.8	13	36	58
**23**	9.3	10	30	51
**24**	8.8	11	32	53
Vit. C	0.8	65	88	96

#### 3.2.4 Structure–Activity Relationship

The structure–activity relationship (SAR) analysis based on the IC_50_ values and percentage inhibition data revealed a clear correlation between structural features and antioxidant activity. The results indicate that antioxidant potency is mainly governed by the ability of the compounds to donate hydrogen atoms or electrons and to stabilize the resulting radical species.

Compounds **17** and **18** exhibited the highest antioxidant activity, with IC_50_ values of 2.3 and 2.5 μg/mL, respectively. These compounds also displayed high inhibition percentages of 89% and 87%, respectively, at 10 μg/mL. Similarly, compound **13** showed remarkable antioxidant activity with an IC_50_ value of 3.3 μg/mL and 83% inhibition at the highest tested concentration. The superior activity of these compounds may be attributed to the presence of structural features that enhance radical‐scavenging ability, such as extended conjugation and electron‐donating substituents, which facilitate stabilization of the generated radical intermediates.

Compounds **10** and **12** also demonstrated strong antioxidant activity, with IC_50_ values of 3.8 and 4.3 μg/mL, respectively, and inhibition percentages of 80% and 78% at 10 μg/mL. Furthermore, compounds **16** (IC_50_ = 4.6 μg/mL) and **15** (IC_50_ = 5.0 μg/mL) exhibited comparable activity, suggesting that an appropriate molecular framework combined with favorable electronic properties contributes significantly to antioxidant performance. These observations emphasize the importance of maintaining an optimal balance between molecular architecture and electron‐donating capability.

Moderately active compounds such as **9** (IC_50_ = 5.8 μg/mL), **19** (6.3 μg/mL), and **21** (6.8 μg/mL) displayed inhibition percentages of 68%, 66%, and 63%, respectively, at 10 μg/mL. The reduced activity of these compounds may be attributed to weaker electron‐donating ability or less efficient delocalization of the generated radical species. Likewise, compounds **20**, **22**, **23**, and **24** exhibited moderate to low antioxidant activity, with IC_50_ values ranging from 7.8 to 9.3 μg/mL, indicating that even minor structural modifications can markedly influence antioxidant efficiency.

In contrast, compounds **1**, **2**, and **5** displayed the weakest antioxidant activity, with IC_50_ values of 22.0, 21.0, and 16.9 μg/mL, respectively. These compounds produced low inhibition percentages that did not exceed 23%–33% at 10 μg/mL, suggesting either a lack of effective hydrogen‐donating groups or the presence of structural features that limit interaction with free radicals. Reduced conjugation, steric hindrance, and insufficient stabilization of radical intermediates may also contribute to their diminished activity.

Overall, the SAR analysis indicates that electronic properties, particularly the presence of electron‐donating groups, together with the degree of conjugation and molecular planarity, play a crucial role in determining antioxidant activity within this series. The most active compounds, namely **17**, **18**, and **13**, were characterized by enhanced electron density and efficient radical stabilization, whereas compounds lacking these features exhibited significantly lower potency. These findings provide valuable guidance for the rational design of more potent antioxidant agents within this class of compounds.

Although oxidative stress is closely associated with cancer development and progression, the antioxidant activity observed for the synthesized compounds should not be considered a direct predictor of their cytotoxic effects. The antioxidant assay was included to provide additional information regarding the biological profile of the synthesized derivatives. Based on the current data, no direct relationship between antioxidant activity and cytotoxic potency could be established.

#### 3.2.5 Molecular Docking Study

**FIGURE 2:**
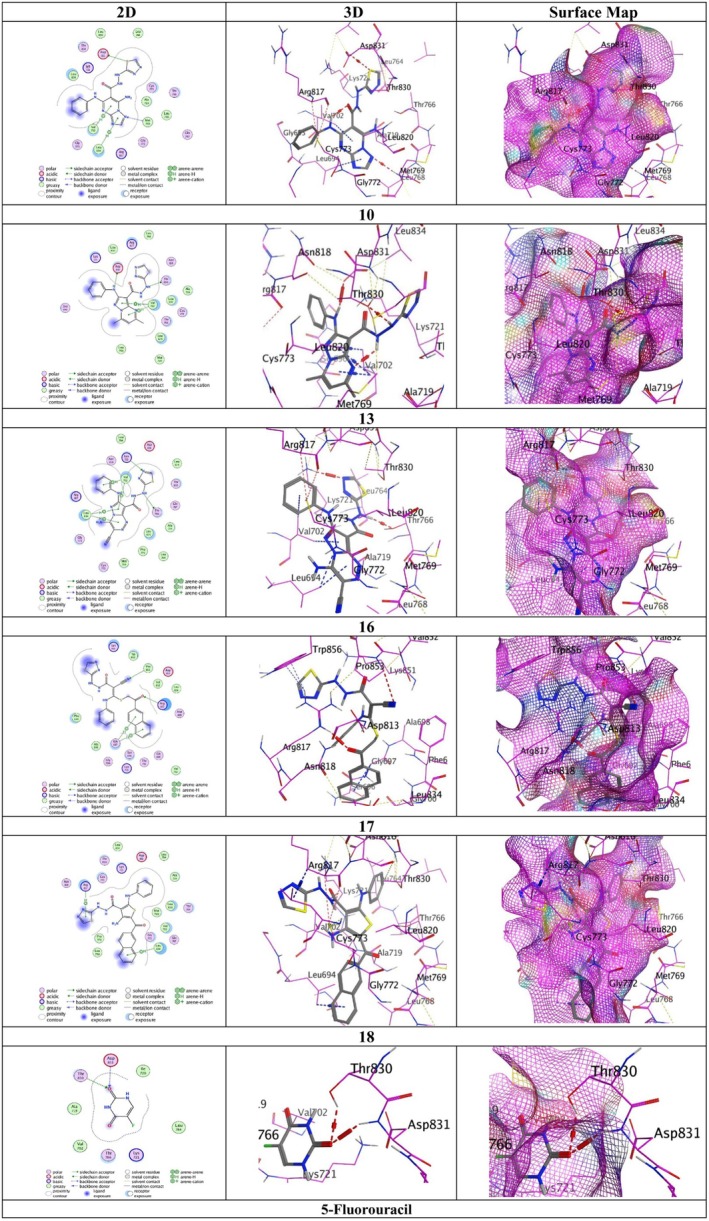
Binding images between derivatives **10**, **13**, **16**, **17**, **18**, and 5‐fluorouracil with PDB ID: 1M17.

The selected derivatives **10**, **13**, **16**, **17**, and **18** were docked by captivating a selected PDB ID: 1M17 protein, using the M.O.E program and their fallouts were recognized (Table 3 and Figure 2). Derivative **18** exhibited the strongest binding score (S = −8.1425 kcal/mol), greater than the other derivatives by a significant margin. This was followed by derivatives **17** (S = −7.4928), **16** (S = −7.4410), **13** (S = −7.3674), and **10** (S = −7.2119). The 5‐fluorouracil was the lowest binding; confirmative the influences of the heterocyclic moieties present in these Derivatives. In the meantime, derivative **10** formed a H‐bond through the S14‐atom of the thiadiazole ring with Asp831 and an H‐acceptor bond by the N10‐atom of the triazole ring with Met769, a key residue in the EGFR hinge district. Additional two pi‐H stackings with Leu694 and Val702 contributed to hydrophobic stabilization. Moreover, derivative **13** showed two H‐donor bonds from the hydrazone moiety and aniline ring with Thr830 and Asp831, respectively, and pi‐H stacking with Val702. The existence of two pi‐H stackings with the same active pocket Val702 recommends a strong hydrophobic fit. However, derivative **16** displayed diverse bindings, including H‐donor (Thr766), H‐acceptor (Lys721), and four pi‐H stackings involving triazine, pyrazole, and phenyl rings with Leu694 and Val702. This wide‐ranging hydrophobic appointment probably explains its promising binding energy in spite of lacking the highest score. Though, compound **17** uniquely included two H‐acceptor bonds from a ketonic oxygen (8) atom and a nitrogen 27 atom of the nitrile group with Arg817 and Pro853, respectively, with two pi‐H stackings from two naphthalene rings with Gly697. This bidentate hydrophobic binding may improve the binding specificity. Furthermore, the 5‐Fluoro reference, in spite of being a known pharmacophore, completed poorly (S = −4.3288 kcal/mol), forming only two H‐acceptor bonds with both of Thr830 and Asp831, and absence of any hydrophobic ring bindings. This highlights the role of extended heterocyclic rings in achieving high‐affinity binding to EGFR.

**TABLE 3:** Docking results of the synthesized derivatives with higher cytotoxicity.

Der.	S (Kcal/mol)	RMSD	Ligand bindings with the amino‐acid residues	Types of interactions	Distance (Å)
**10**	−7.2119	1.5111	S14 of the thiadiazole‐ring with Asp831	H‐donor	3.85
			N10 of the triazole‐ring with Met769	H‐acceptor	3.44
			The triazole‐ring with Leu694	pi‐H	3.90
			The pyrimidine‐ring with Val702	pi‐H	4.35
**13**	−7.3674	1.8321	N 20 of the hydrazone moiety with Thr830	H‐donor	3.00
			N 21 of the aniline‐ring with Asp831	H‐donor	3.14
			The triazole‐ring with Val702	Pi‐H	4.05
			The pyrimidine‐ring with Val702	Pi‐H	4.07
**16**	−7.4410	1.5675	N 19 of the hydrazone moiety with Thr766	H‐donor	3.16
			N 17 of the thiadiazole‐ring with Lys721	H‐acceptor	3.32
			The triazine‐ring with Leu694	pi‐H	3.67
			The pyrazole‐ring with Leu694	pi‐H	4.42
			The pyrazole‐ring with Val702	pi‐H	4.35
			The phenyl‐ring with Val702	pi‐H	4.46
**17**	−7.4928	1.2987	O 8 of the ketonic group with Arg817	H‐acceptor	3.08
			N 27 of the nitrile group with Pro853	H‐acceptor	3.36
			The 1st naphthalene‐ring with Gly697	pi‐H	4.06
			The 2nd naphthalene‐ring with Gly697	pi‐H	4.73
**18**	−8.1425	1.3759	One of the naphthalene‐ring with Leu694	pi‐H	3.72
			The thiadiazole‐ring with Arg817	pi‐H	3.78
**5‐Flouro**	−4.3288	1.4418	O 3 of the pyrimidinedione‐ring with Thr830	H‐acceptor	3.06
			O 3 of the pyrimidinedione‐ring with Asp831	H‐acceptor	3.05

## 4 Conclusion

This study used a variety of spectroscopic techniques to successfully synthesize and analyze a novel series of heterocyclic compounds comprising the 1,3,4‐thiadiazole scaffold. The newly synthesized cyanoacetohydrazide derivative demonstrated its synthetic value and uniqueness by acting as a flexible key intermediary for building a variety of structurally different heterocyclic systems. Several of the produced compounds showed notable antioxidant and anticancer properties, according to the biological evaluation. Compounds **17**, **18**, and **13** outperformed the reference medication 5‐fluorouracil in terms of cytotoxic potency against HePG‐2 and MCF‐7 cancer cell lines while still exhibiting respectable selectivity toward normal cells. These substances also demonstrated substantial antioxidant action, indicating their potential as dual‐purpose medicinal agents. Electronic properties, lipophilicity, and structural elements like electron‐donating or electron‐withdrawing substituents and molecular conjugation have a significant impact on biological activity, according to structure–activity relationship (SAR) study. These elements are essential for improving cellular absorption and biological target engagement. Additionally, favorable binding interactions between certain drugs and the EGFR tyrosine kinase domain were revealed by molecular docking studies, which corroborated the experimental results and may account for the compounds' apparent anticancer effect. Overall, the findings show that the 1,3,4‐thiadiazole molecule is a viable scaffold for the creation of novel antioxidant and anticancer drugs. To get these molecules closer to possible therapeutic uses, future research should concentrate on more structural optimization, in vivo assessment, and thorough mechanistic analyses.

## Author Contributions

**Nadiyah M. Alshammari:** data curation, software, validation, writing – review and editing. **Nashwa M. El‐Metwaly:** investigation, writing – original draft, visualization, formal analysis. **Fatimah A. Alotaibi:** writing – review and editing, writing – original draft, formal analysis, resources. **Abdulmajeed F. Alrefaei:** formal analysis, methodology, software, writing – review and editing. **Manal S. Ebaid:** formal analysis, software, validation, writing – review and editing, writing – original draft, investigation. **Hanadi A. Katouah:** supervision, project administration, validation, visualization, writing – review and editing. **Hossa F. Alshareef:** writing – review and editing, writing – original draft, investigation, methodology. **Samar J. Almehmadi:** data curation, resources, formal analysis, validation, methodology.

## Funding

This work was supported by Northern Border University (NBU‐FFR‐2026‐80‐Xx).

## Ethics Statement

The authors have nothing to report.

## Conflicts of Interest

The authors declare no conflicts of interest.

## Supporting information


**Figure S1:** ^1^H‐NMR of compound **1**.
**Figure S2:** ^13^C‐NMR of compound **1**.
**Figure S3:** Mass spectrum of compound **1**.
**Figure S4:** ^1^H‐NMR of compound **2**.
**Figure S5:** ^13^C‐NMR of compound **2**.
**Figure S6:** Mass spectrum of compound **2**.
**Figure S7:** ^1^H‐NMR of compound **5**.
**Figure S8:** ^13^C‐NMR of compound **5**.
**Figure S9:** Mass spectrum of compound **5**.
**Figure S10:** ^1^H‐NMR of compound **7**.
**Figure S11:** ^13^C‐NMR of compound **7**.
**Figure S12:** Mass spectrum of compound **7**.
**Figure S13:** ^1^H‐NMR of compound **9**.
**Figure S14:** ^13^C‐NMR of compound **9**.
**Figure S15:** Mass spectrum of compound **9**.
**Figure S16:** ^1^H‐NMR of compound **10**.
**Figure S17:** ^13^C‐NMR of compound **10**.
**Figure S18:** Mass spectrum of compound **10**.
**Figure S19:** ^1^H‐NMR of compound **11**.
**Figure S20:** ^13^C‐NMR of compound **11**.
**Figure S21:** Mass spectrum of compound **11**.
**Figure S22:** ^1^H‐NMR of compound **12**.
**Figure S23:** ^13^C‐NMR of compound **12**.
**Figure S24:** Mass spectrum of compound **12**.
**Figure S25:** ^1^H‐NMR of compound **13**.
**Figure S26:** ^13^C‐NMR of compound **13**.
**Figure S27:** Mass spectrum of compound **13**.
**Figure S28:** ^1^H‐NMR of compound **15**.
**Figure S29:** ^13^C‐NMR of compound **15**.
**Figure S30:** Mass spectrum of compound **15**.
**Figure S31:** ^1^H‐NMR of compound **16**.
**Figure S32:** ^13^C‐NMR of compound **16**.
**Figure S33:** Mass spectrum of compound **16**.
**Figure S34:** ^1^H‐NMR of compound **17**.
**Figure S35:** ^13^C‐NMR of compound **17**.
**Figure S36:** Mass spectrum of compound **17**.
**Figure S37:** ^1^H‐NMR of compound **19**.
**Figure S38:** ^13^C‐NMR of compound **19**.
**Figure S39:** Mass spectrum of compound **19**.
**Figure S40:** ^1^H‐NMR of compound **21**.
**Figure S41:** ^13^C‐NMR of compound **21**.
**Figure S42:** Mass spectrum of compound **21**.
**Figure S43:** ^1^H‐NMR of compound **23**.
**Figure S44:** ^13^C‐NMR of compound **23**.
**Figure S45:** Mass spectrum of compound **23**.
**Figure S46:** ^1^H‐NMR of compound **18**.
**Figure S47:** ^13^C‐NMR of compound **18**.
**Figure S48:** Mass spectrum of compound **18**.
**Figure S49:** ^1^H‐NMR of compound **20**.
**Figure S50:** ^13^C‐NMR of compound **20**.
**Figure S51:** Mass spectrum of compound **20**.
**Figure S52:** ^1^H‐NMR of compound **22**.
**Figure S53:** ^13^C‐NMR of compound **22**.
**Figure S54:** Mass spectrum of compound **22**.
**Figure S55:** ^1^H‐NMR of compound **24**.
**Figure S56:** ^13^C‐NMR of compound **24**.
**Figure S57:** Mass spectrum of compound **24**.

## Data Availability

The data that support the findings of this study are available on request from the corresponding author. The data are not publicly available due to privacy or ethical restrictions.
