# Supplementary material for: Synthesis, Characterization, Molecular Docking Study and Biological Evaluation of Novel 1,3,4‐Thiadiazole‐Based Heterocycles as Potential Anticancer and Antioxidant Agents
Source: Chem Biol Drug Des. 2026 Jul 15;108(1):e70361. doi: 10.1111/cbdd.70361 (PMC13370520; doi:10.1111/cbdd.70361)
Supplement: Supplementary file 1 — Figure S1: 1H‐NMR of compound 1. Figure S2: 13C‐NMR of compound 1. Figure S3: Mass spectrum of compound 1. Figure S4: 1H‐NMR of compound 2. Figure S5: 13C‐NMR of compound 2. Figure S6: Mass spectrum of compound 2. Figure S7: 1H‐NMR of compound 5. Figure S8: 13C‐NMR of compound 5. Figure S9: Mass spectrum of compound 5. Figure S10: 1H‐NMR of compound 7. Figure S11: 13C‐NMR of compound 7. Figure S12: Mass spectrum of compound 7. Figure S13: 1H‐NMR of compound 9. Figure S14: 13C‐NMR of compound 9. Figure S15: Mass spectrum of compound 9. Figure S16: 1H‐NMR of compound 10. Figure S17: 13C‐NMR of compound 10. Figure S18: Mass spectrum of compound 10. Figure S19: 1H‐NMR of compound 11. Figure S20: 13C‐NMR of compound 11. Figure S21: Mass spectrum of compound 11. Figure S22: 1H‐NMR of compound 12. Figure S23: 13C‐NMR of compound 12. Figure S24: Mass spectrum of compound 12. Figure S25: 1H‐NMR of compound 13. Figure S26: 13C‐NMR of compound 13. Figure S27: Mass spectrum of compound 13. Figure S28: 1H‐NMR of compound 15. Figure S29: 13C‐NMR of compound 15. Figure S30: Mass spectrum of compound 15. Figure S31: 1H‐NMR of compound 16. Figure S32: 13C‐NMR of compound 16. Figure S33: Mass spectrum of compound 16. Figure S34: 1H‐NMR of compound 17. Figure S35: 13C‐NMR of compound 17. Figure S36: Mass spectrum of compound 17. Figure S37: 1H‐NMR of compound 19. Figure S38: 13C‐NMR of compound 19. Figure S39: Mass spectrum of compound 19. Figure S40: 1H‐NMR of compound 21. Figure S41: 13C‐NMR of compound 21. Figure S42: Mass spectrum of compound 21. Figure S43: 1H‐NMR of compound 23. Figure S44: 13C‐NMR of compound 23. Figure S45: Mass spectrum of compound 23. Figure S46: 1H‐NMR of compound 18. Figure S47: 13C‐NMR of compound 18. Figure S48: Mass spectrum of compound 18. Figure S49: 1H‐NMR of compound 20. Figure S50: 13C‐NMR of compound 20. Figure S51: Mass spectrum of compound 20. Figure S52: 1H‐NMR of compound 22. Figure S53: 13C‐NMR of compound 22. Figure S54: Mass spectrum of com [file CBDD-108-e70361-s001.docx]

**Experiment**

**Preparation of 2-Hydrazinyl-1,3,4-thiadiazole**

A solution of 2-amino-1,3,4-thiadiazole (2.02 g, 0.02 mol) in dil. hydrochloric acid (5 mL) was cooled to 0–5 °C. A cold aqueous solution of sodium nitrite (1.38 g, 0.02 mol) was added dropwise with stirring while maintaining the temperature below 5 °C. The reaction mixture was stirred for an additional 60 min to ensure complete formation of the corresponding diazonium salt. A solution of stannous chloride dihydrate (2.26 g, 0.01 mol) in concentrated hydrochloric acid (5 mL) was then added gradually to the freshly prepared diazonium solution under continuous cooling. Stirring was continued at 0–5 °C for 2 h and then at room temperature for 2 h. After completion of the reaction, the mixture was neutralized with aqueous sodium hydroxide solution to pH 7. The precipitated product was collected by filtration, washed with water, dried, and recrystallized from ethanol to afford 2-hydrazinyl-1,3,4-thiadiazole as 70 % yield; m.p. 152-154 °C [Lit. 156-157 °C **[1]**].

**Synthesis of 2-cyano-N'-(1,3,4-thiadiazol-2-yl)acetohydrazide (1)**

A catalytic amount of piperidine was used to reflux 2-hydrazino-1,3,4-thiadiazole and ethyl cyanoacetate (1.16 g, 0.01 mol) and benzylidene malononitrile (1.13 g, 0.01 mol) in DMF (20 mL) for three hours. For the whole night, the reaction mixture was left at room temperature. The solid substance was collected, filtered, dried, and recrystallized after being separated from ethanol.

Yield, 82%; m.p. 176-178 °C; IR (KBr): ν_max_, cm^-1^: 3230 (NH), 2219 (CN), 1675 (CO), 1620 (C=N); ^1^H-NMR (DMSO-*d_6_*) *δ* ppm: 3.10 (s, 2H, CH_2_), 8.37 (s, 1H, NH), 9.20 (s, 1H, thiadiazole-CH), 10.31 (s, 1H, NH) **(Figure S1)**; ^13^C-NMR (DMSO-*d_6_*) *δ* ppm: 26.9, 125.4, 152.9, 170.5, 174.2 **(Figure S2)**. MS (m/z, %): 183 (M^+^, 60) **(Figure S3)**. Anal. Calced for C_5_H_5_N_5_OS (183.19): C, 32.78; H, 2.75; N, 38.23%. Found: C, 32.72; H, 2.70; N, 38.19%.

**Synthesis of 3-(2-(1,3,4-thiadiazol-2-yl)hydrazineyl)-N'-hydroxy-3-oxopropanimidamide (2)**

Compound **1** (0.36 g, 0.002 mol) and hydroxylamine hydrochloride (0.14 g, 0.002 mol) were refluxed in 25 mL of boiling EtOH with three drops of TEA for three hours. Compound **2** was produced by filtering and recrystallizing the solid material that was obtained after cooling from ethanol.

Yield, 76%; m.p. 182-184 °C; IR (KBr): ν_max_, cm^-1^: 3460 (OH), 3420 (NH_2_), 3230 (NH), 3210 (NH), 1680 (CO), 1618 (C=N); ^1^H-NMR (DMSO-*d_6_*) *δ* ppm: 3.19 (s, 2H, CH_2_), 6.59 (s, 2H, NH_2_), 8.31 (s, 1H, NH), 9.10 (s, 1H, OH), 9.30 (s, 1H, thiadiazole-CH), 10.40 (s, 1H, NH) **(Figure S4)**; ^13^C-NMR (DMSO-*d_6_*) *δ* ppm: 44.1, 152.5, 156.8, 170.6, 174.9 **(Figure S5)**. MS (m/z, %): 216 (M^+^, 65) **(Figure S6)**. Anal. Calced for C_5_H_8_N_6_O_2_S (216.22): C, 27.78; H, 3.73; N, 38.87%. Found: C, 27.73; H, 3.68; N, 38.83%.

**Synthesis of 2-((1,3,4-thiadiazol-2-yl)amino)-5-amino-2,4-dihydro-3H-pyrazol-3-one (5)**

Acetic anhydride (25 mL) and compound **2** (0.216 g, 0.001 mol) were heated in a water bath for eight hours. Compound **5** was obtained by filtering the solid substance and crystallizing it from hot ethanol.

Yield, 78%; m.p. 196-198 °C; IR (KBr): ν_max_, cm^-1^: IR: 3430 (NH_2_), 3310 (NH), 1682 (CO), 1622 (C=N), 1600 (C=C); ^1^H-NMR (DMSO-*d_6_*) *δ* ppm: 3.10 (s, 2H, CH_2_), 6.49 (s, 2H, NH_2_), 8.30 (s, 1H, NH), 9.25 (s, 1H, thiadiazole-CH) **(Figure S7)**; ^13^C-NMR (DMSO-*d_6_*) *δ* ppm: 74.8, 152.1, 160.5, 171.4, 174.8 **(Figure S8)**. MS (m/z, %): 198 (M^+^, 59) **(Figure S9)**. Anal. Calced for C_5_H_6_N_6_OS (198.20): C, 30.30; H, 3.05; N, 42.40%. Found: C, 30.24; H, 3.00; N, 42.35%.

**Synthesis of 2-(4-oxo-4,5-dihydrothiazol-2-yl)-N'-(1,3,4-thiadiazol-2-yl)acetohydrazide (7)**

Compound **1** (0.18 g, 0.001 mol) and thioglycolic acid (0.09 mL, 0.001 mol) in glacial acetic acid (20 mL) were refluxed for six hours. Compound **7** was produced by crystallizing heated ethanol and cooling it to room temperature.

Yield, 72%; m.p. 201-203 °C; IR (KBr): ν_max_, cm^-1^: IR: 3220 (NH), 1710 (C=O), 1682 (amidic CO), 1615 (C=N); ^1^H-NMR (DMSO-*d_6_*) *δ* ppm: 2.95 (s, 2H, CH_2_), 4.20 (s, 2H, thiazole-CH_2_), 8.46 (s, 1H, NH), 9.39 (s, 1H, thiadiazole-CH), 10.46 (s, 1H, NH) **(Figure S10)**; ^13^C-NMR (DMSO-*d_6_*) *δ* ppm: 38.6, 42.7, 152.9, 164.5, 170.0, 174.0, 177.8 **(Figure S11)**. MS (m/z, %): 257 (M^+^, 60) **(Figure S12)**. Anal. Calced for C_7_H_7_N_5_O_2_S_2_ (257.29): C, 32.68; H, 2.74; N, 27.22%. Found: C, 32.61; H, 2.69; N, 27.15%.

**Synthesis of 2-cyano-3-(methylthio)-3-(phenylamino)-N'-(1,3,4-thiadiazol-2-yl)acrylohydrazide (9)**

Phenyl isothiocyanate (0.28 mL, 0.002 mol) was added to a solution of **1** (0.36 g, 0.002 mol) in DMF (25 mL) and KOH (0.011 g, 0.002 mol), and the reaction mixture was agitated for six hours to give the intermediate **8**. After adding dimethyl sulfate (0.18 mL, 0.002 mol) to this salt and stirring for an additional three hours, the mixture was put to ice-cold water. Compound **9** was produced by filtering and recrystallizing the resultant product from EtOH.

Yield, 70%; m.p. 190-192 °C; IR (KBr): ν_max_, cm^-1^: 3300 (NH), 2219 (CN), 1675 (CO), 1623 (C=N); ^1^H-NMR (DMSO-*d_6_*) *δ* ppm: 2.30 (s, 3H, S-CH_3_), 6.98-7.15 (m, 5H, Ar-H), 8.21 (s, 1H, NH), 9.26 (s, 1H, NH), 9.60 (s, 1H, thiadiazole-CH), 10.81 (s, 1H, NH) **(Figure S13)**; ^13^C-NMR (DMSO-*d_6_*) *δ* ppm: 17.4, 69.6, 114.6, 122.0, 125.1 (2C), 129.5 (2C), 136.7, 152.4, 155.6, 172.9, 178.4 **(Figure S14)**. MS (m/z, %): 332 (M^+^, 52) **(Figure S15)**. Anal. Calced for C_13_H_12_N_6_OS_2_ (332.40): C, 46.97; H, 3.64; N, 25.28%. Found: C, 46.91; H, 3.60; N, 25.23%.

**Synthesis of 7-amino-5-(phenylamino)-N'-(1,3,4-thiadiazol-2-yl)-[1,2,4]triazolo[1,5-a]pyrimidine-6-carbohydrazide (10)**

Compound **9** (0.33 g, 0.001 mol) and 3-amino-1H-1,2,4-triazole (0.08 g, 0.001 mol) were dissolved in 25 mL of pyridine, refluxed for three hours, and then allowed to cool. A few drops of HCl were added to the reaction mixture after it was cooled in ice-cold water. To obtain **10**, the separated solid material was recrystallized from EtOH.

Yield, 76%; m.p. 215-217 °C; IR (KBr): ν_max_, cm^-1^: 3425 (NH_2_), 3315 (NH), 1681 (CO), 1617 (C=N), 1600 (C=C); ^1^H-NMR (DMSO-*d_6_*) *δ* ppm: 7.27-7.49 (m, 7H, Ar-H + NH_2_), 8.30 (s, 1H, NH), 8.39 (s, 1H, triazole-CH), 8.51 (s, 1H, NH), 9.16 (s, 1H, thiadiazole-CH), 10.49 (s, 1H, NH) **(Figure S16)**; ^13^C-NMR (DMSO-*d_6_*) *δ* ppm: 98.5, 116.8 (2C), 122.6, 129.7 (2C), 140.3, 152.1, 154.1, 156.0, 163.0, 171.4, 172.9, 175.2 **(Figure S17)**. MS (m/z, %): 368 (M^+^, 64) **(Figure S18)**. Anal. Calced for C_14_H_12_N_10_OS (368.38): C, 45.65; H, 3.28; N, 38.02%. Found: C, 45.59; H, 3.22; N, 37.95%.

**Synthesis of 2-cyano-2-(imidazolidin-2-ylidene)-N'-(1,3,4-thiadiazol-2-yl)acetohydrazide (11)**

In a ethanolic solution (25 mL), an equimolar mixture of compound **9** (0.33 g, 0.001 mol) and ethylenediamine (0.06 g, 0.001 mol) was heated for three hours before being allowed to cool. Filtration was used to separate the resulting precipitate, which then recrystallized from EtOH to produce **11**.

Yield, 73%; m.p. 186-188 °C; IR (KBr): ν_max_, cm^-1^: 3235 (NH), 2220 (CN), 1683 (CO), 1625 (C=N), 1605 (C=C); ^1^H-NMR (DMSO-*d_6_*) *δ* ppm: 3.75 (s, 4H, 2CH_2_), 6.39 (s, 2H, 2NH), 8.19 (s, 1H, NH), 9.11 (s, 1H, NH), 9.22 (s, 1H, thiadiazole-CH) **(Figure S19)**; ^13^C-NMR (DMSO-*d_6_*) *δ* ppm: 43.0 (2C), 57.4, 116.5, 152.9, 165.6, 173.1, 185.7 **(Figure S20)**. MS (m/z, %): 251 (M^+^, 55) **(Figure S21)**. Anal. Calced for C_8_H_9_N_7_OS (251.27): C, 38.24; H, 3.61; N, 39.02%. Found: C, 38.19; H, 3.55; N, 38.96%.

**Synthesis of 5-amino-3-(phenylamino)-N'-(1,3,4-thiadiazol-2-yl)-1H-pyrazole-4-carbohydrazide (12)**

An ethanolic (25 mL) solution of **9** (0.33 g, 0.001 mol) was mixed with hydrazine hydrate (0.05 mL, 0.001 mol), refluxed for three hours, and then allowed to cool. Compound **12** was obtained by filtering out and recrystallizing the solid precipitate that resulted from EtOH.

Yield, 70%; m.p. 210-212 °C; IR (KBr): ν_max_, cm^-1^: 3445 (NH_2_), 3310 (NH), 1673 (CO), 1625 (C=N), 1600 (C=C); ^1^H-NMR (DMSO-*d_6_*) *δ* ppm: 6.40 (s, 2H, NH_2_), 7.05-7.17 (m, 5H, Ar-H), 8.35 (s, 1H, NH), 9.29 (s, 1H, thiadiazole-CH), 10.19 (s, 1H, NH), 10.50 (s, 1H, Ph-NH), 12.59 (s, 1H, pyrazole-NH) **(Figure S22)**; ^13^C-NMR (DMSO-*d_6_*) *δ* ppm: 84.7, 117.7 (2C), 122.5, 129.5 (2C), 140.8, 151.0, 151.9, 152.9, 165.0, 173.7 **(Figure S23)**. MS (m/z, %): 316 (M^+^, 68) **(Figure S24)**. Anal. Calced for C_12_H_12_N_8_OS (316.34): C, 45.56; H, 3.82; N, 35.42%. Found: C, 45.50; H, 3.77; N, 35.36%.

**Synthesis of 5,7-dimethyl-2-(phenylamino)-N'-(1,3,4-thiadiazol-2-yl)pyrazolo[1,5-a]pyrimidine-3-carbohydrazide (13)**

The reaction mixture was added dropwise to ice-cold water after an equimolar amounts of **12** (0.31 g, 0.001 mol) and acetylacetone (0.10 mL, 0.001 mol) was added to a (25 mL) glacial acetic acid solution and heated for three hours. Filtration was used to separate the resulting solid substance, which was then refined by recrystallization from EtOH/benzene to yield **13**.

Yield, 73%; m.p. 221-223 °C; IR (KBr): ν_max_, cm^-1^: 3235 (NH), 1686 (CO), 1626 (C=N), 1595 (C=C); ^1^H-NMR (DMSO-*d_6_*) *δ* ppm: 2.39 (s, 3H, CH_3_), 2.81 (s, 3H, CH_3_), 6.96-7.19 (m, 6H, Ar-H + pyrimidine-CH), 8.41 (s, 1H, NH), 9.18 (s, 1H, thiadiazole-CH), 10.39 (s, 1H, NH), 10.73 (s, 1H, NH) **(Figure S25)**; ^13^C-NMR (DMSO-*d_6_*) *δ* ppm: 17.2, 24.5, 97.0, 108.6, 117.6 (2C), 122.1, 129.5 (2C), 140.5, 145.5, 146.8, 153.7 (2C), 164.9 (2C), 172.8 **(Figure S26)**. MS (m/z, %): 380 (M^+^, 50) **(Figure S27)**. Anal. Calced for C_17_H_16_N_8_OS (380.43): C, 53.67; H, 4.24; N, 29.46%. Found: C, 53.61; H, 4.20; N, 29.41%.

**Synthesis of N-(4-(2-(1,3,4-thiadiazol-2-yl)hydrazine-1-carbonyl)-3-(phenylamino)-1H-pyrazol-5-yl)carbonohydrazonoyl dicyanide (15)**

1. Preparation of diazonium salt:

To an ice-cold solution of compound **12** (0.31 g, 0.001 mol) in a mixture of acetic acid and concentrated HCl [(8:2) 10 mL (1/4) Vol.] was added to a solution of sodium nitrite (0.069 g, 0.001 mol, in 2 mL water) dropwise.

1. Coupling reaction:

Over the course of 25 minutes, a cold solution of malononitrile (0.066 g, 0.001 mol) in pyridine (10 mL) was added dropwise to the above-formed diazonium chloride solution while being continuously stirred. The reaction mixture was refrigerated for the entire night. Compounds **15** were obtained by filtering and recrystallizing the distinct solid material from EtOH.

Yield, 76%; m.p. 231-233 °C; IR (KBr): ν_max_, cm^-1^: 3315 (NH), 2220, 2219 (2 CN), 1675 (CO), 1622 (C=N), 1605 (C=C); ^1^H-NMR (DMSO-*d_6_*) *δ* ppm: 7.25-7.40 (m, 5H, Ar-H), 8.25 (s, 1H, NH), 9.20 (s, 1H, thiadiazole-CH), 10.19 (s, 1H, NH), 10.32 (s, 1H, hydrazo-NH), 10.44 (s, 1H, Ph-NH), 12.49 (s, 1H, pyrazole-NH) **(Figure S28)**; ^13^C-NMR (DMSO-*d_6_*) *δ* ppm: 84.6 (2C), 112.6 (2C), 117.6 (2C), 122.2, 129.5 (2C), 140.7, 145.2, 150.2, 153.4, 164.7, 173.1 **(Figure S29)**. MS (m/z, %): 393 (M^+^, 53) **(Figure S30)**. Anal. Calced for C_15_H_11_N_11_OS (393.39): C, 45.80; H, 2.82; N, 39.17%. Found: C, 45.74; H, 2.77; N, 39.11%.

**Synthesis of 4-amino-3-cyano-7-(phenylamino)-N'-(1,3,4-thiadiazol-2-yl)pyrazolo[5,1-c][1,2,4]triazine-8-carbohydrazide (16)**

The reaction mixture was added dropwise to ice-cold water after an equimolar amounts of **15** (0.40 g, 0.0008 mol) was added to a (20 mL) glacial acetic acid solution and heated for three hours. To obtain pyrazolo[5,1-c][1,2,4]triazine **16**, the solid material was separated by filtration and refined by recrystallization from EtOH/DMF.

Yield, 78%; m.p. 241-243 °C; IR (KBr): ν_max_, cm^-1^: 3430 (NH_2_), 3310 (NH), 2218 (CN), 1623 (C=N), 1600 (C=C), 1560 (N=N); ^1^H-NMR (DMSO-*d_6_*) *δ* ppm: 6.29 (s, 2H, NH_2_), 7.26-7.67 (m, 5H, Ar-H), 8.24 (s, 1H, NH), 9.38 (s, 1H, thiadiazole-CH), 10.34 (s, 1H, NH), 10.70 (s, 1H, Ph-NH) **(Figure S31)**; ^13^C-NMR (DMSO-*d_6_*) *δ* ppm: 55.5, 97.5, 113.2, 117.9 (2C), 122.5, 129.4 (2C), 140.7, 145.0, 149.7, 152.5 (2C), 164.6, 173.7 **(Figure S32)**. MS (m/z, %): 393 (M^+^, 68) **(Figure S33)**. Anal. Calced for C_15_H_11_N_11_OS (393.39): C, 45.80; H, 2.82; N, 39.17%. Found: C, 45.76; H, 2.75; N, 39.13%.

**The acyclic intermediates 17, 19, 21, and 23 are synthesized**

***General procedure***

Compound **8** (0.001 mol) equimolecular quantities of naphthoyl acetyl bromide, chloroacetone, chloroacetonitrile, and/or ethyl bromoacetate were reacted in ethanol at room temperature for six hours. After that, they were left for a whole day at that temperature. Compounds **17**, **19**, **21**, and **23** were obtained by filtering off the reaction mixture, drying it, rinsing it with water, and then crystallizing it from ethanol.

**2-Cyano-3-((2-(naphthalen-2-yl)-2-oxoethyl)thio)-3-(phenylamino)-N'-(1,3,4-thiadiazol-2-yl)acrylohydrazide (17)**

Yield, 73%; m.p. 217-219 °C; IR (KBr): ν_max_, cm^-1^: 3225 (NH), 2220 (CN), 1720 (CO), 1680 (amidic CO), 1621 (C=N), 1603 (C=C); ^1^H-NMR (DMSO-*d_6_*) *δ* ppm: 4.79 (s, 2H, CH_2_), 7.17-7.50 (m, 11H, Ar-H), 8.40 (s, 1H, NH), 8.70 (s, 1H, Ar-H), 9.28 (s, 1H, NH), 9.60 (s, 1H, Thiadiazole-CH), 10.63 (s, 1H, NH) **(Figure S34)**; ^13^C-NMR (DMSO-*d_6_*) *δ* ppm: 38.5, 69.6, 112.8, 122.1, 123.0, 124.7 (2C), 125.7, 127.6, 128.4 (2C), 130.0 (4C), 130.8 (2C), 137.9 (2C), 152.8, 165.5, 173.5, 179.0, 194.9 **(Figure S35)**. MS (m/z, %): 486 (M^+^, 66) **(Figure S36)**. Anal. Calced for C_24_H_18_N_6_O_2_S_2_ (486.57): C, 59.24; H, 3.73; N, 17.27%. Found: C, 59.20; H, 3.70; N, 17.22%.

**2-Cyano-3-((2-oxopropyl)thio)-3-(phenylamino)-N'-(1,3,4-thiadiazol-2-yl)acrylohydrazide (19)**

Yield, 73%; m.p. 197-199 °C; IR (KBr): ν_max_, cm^-1^: 3235 (NH), 2219 (CN), 1710 (C=O), 1677 (amidic CO), 1625 (C=N), 1600 (C=C); ^1^H-NMR (DMSO-*d_6_*) *δ* ppm: 2.29 (s, 3H, CH_3_), 3.99 (s, 2H, CH_2_), 7.10-7.39 (m, 5H, Ar-H), 8.20 (s, 1H, NH), 9.21 (s, 1H, NH), 9.39 (s, 1H, thiadiazole-CH), 10.65 (s, 1H, NH) **(Figure S37)**; ^13^C-NMR (DMSO-*d_6_*) *δ* ppm: 26.8, 40.6, 69.2, 114.9, 122.9, 124.8 (2C), 128.9 (2C), 136.8, 152.7, 165.1, 172.9, 178.5, 194.0 **(Figure S38)**. MS (m/z, %): 374 (M^+^, 63) **(Figure S39)**. Anal. Calced for C_15_H_14_N_6_O_2_S_2_ (374.44): C, 48.12; H, 3.77; N, 22.44%. Found: C, 48.07; H, 3.70; N, 22.39%.

**2-Cyano-3-((cyanomethyl)thio)-3-(phenylamino)-N'-(1,3,4-thiadiazol-2-yl)acrylohydrazide (21)**

Yield, 77%; m.p. 205-207 °C; IR (KBr): ν_max_, cm^-1^: 3315 (NH), 2220, 2218 (2CN), 1682 (CO), 1622 (C=N), 1602 (C=C); ^1^H-NMR (DMSO-*d_6_*) *δ* ppm: 4.31 (s, 2H, CH_2_), 6.85-7.20 (m, 5H, Ar-H), 8.40 (s, 1H, NH), 9.29 (s, 1H, NH), 9.60 (s, 1H, thiadiazole-CH), 10.55 (s, 1H, NH) **(Figure S40)**; ^13^C-NMR (DMSO-*d_6_*) *δ* ppm: 16.6, 69.7, 114.8, 118.5, 123.7, 126.6 (2C), 129.9 (2C), 136.8, 152.5, 165.7, 173.0, 178.9 **(Figure S41)**. MS (m/z, %): 357 (M^+^, 60) **(Figure S42)**. Anal. Calced for C_14_H_11_N_7_OS_2_ (357.41): C, 47.05; H, 3.10; N, 27.43%. Found: C, 47.00; H, 3.04; N, 27.38%.

**Ethyl 2-((3-(2-(1,3,4-thiadiazol-2-yl)hydrazineyl)-2-cyano-3-oxo-1-(phenylamino)prop-1-en-1-yl)thio)acetate (23)**

Yield, 71%; m.p. 223-225 °C; IR (KBr): ν_max_, cm^-1^: 3310 (NH), 2219 (CN), 1720 (CO), 1682 (amidic CO), 1615 (C=N), 1603 (C=C); ^1^H-NMR (DMSO-*d_6_*) *δ* ppm: 1.49 (t, 3H, CH_3_), 3.89 (s, 2H, CH_2_), 4.30 (q, 2H, CH_2_), 7.19-7.33 (m, 5H, Ar-H), 8.29 (s, 1H, NH), 9.20 (s, 1H, NH), 9.61 (s, 1H, thiadiazole-CH), 10.29 (s, 1H, NH) **(Figure S43)**; ^13^C-NMR (DMSO-*d_6_*) *δ* ppm: 14.7, 33.5, 60.7, 69.4, 114.9, 123.0, 126.0 (2C), 128.6 (2C), 136.8, 152.8, 165.1, 169.8, 173.7, 179.8 **(Figure S44)**. MS (m/z, %): 404 (M^+^, 63) **(Figure S45)**. Anal. Calced for C_16_H_16_N_6_O_3_S_2_ (404.46): C, 47.51; H, 3.99; N, 20.78%. Found: C, 47.45; H, 3.93; N, 20.70%.

**Synthesis of thiophene derivatives** **(18, 20, 22 and 24)**

**Method A:** In 20 mL of DMF, equimolecular amounts of **8** (0.001 mol) and α-halo compounds (0.001 mol) were mixed and refluxed for six hours. The reaction mixture produced the corresponding thiophene derivatives **18**, **20**, **22**, and **24** after cooling, filtration, and recrystallization from ethanol.

**Method B:** The corresponding substituted thiophene derivatives, **18**, **20**, **22**, and **24**, were obtained by refluxing the acyclic intermediates **17**, **19**, **21**, and **23** for four hours in ethanol (20 mL) containing a catalytic quantity of TEA (4 drops).

**5-(2-Naphthoyl)-4-amino-2-(phenylamino)-N'-(1,3,4-thiadiazol-2-yl)thiophene-3-carbohydrazide (18)**

Yield, 70%; m.p. 248-250 °C; IR (KBr): ν_max_, cm^-1^: 3410 (NH_2_), 3320 (NH), 1715 (CO), 1676 (amidic CO), 1622 (C=N), 1603 (C=C); ^1^H-NMR (DMSO-*d_6_*) *δ* ppm: 6.11 (s, 2H, NH_2_), 7.09-7.44 (m, 11H, Ar-H), 8.39 (s, 1H, NH), 8.76 (s, 1H, Ar-H), 9.44 (s, 1H, thiadiazole-CH), 10.30 (s, 1H, NH), 10.71 (s, 1H, NH) **(Figure S46)**; ^13^C-NMR (DMSO-*d_6_*) *δ* ppm: 94.1, 104.5, 118.7 (2C), 122.9, 123.5, 126.0, 126.8, 128.0 (2C), 128.7 (3C), 131.1, 132.0, 133.5 (2C), 143.4 (2C), 153.7, 165.3, 169.6, 174.0, 185.4 **(Figure S47)**. MS (m/z, %): 486 (M^+^, 54) **(Figure S48)**. Anal. Calced for C_24_H_18_N_6_O_2_S_2_ (486.57): C, 59.24; H, 3.73; N, 17.27%. Found: C, 59.18; H, 3.68; N, 17.23%.

**5-Acetyl-4-amino-2-(phenylamino)-N'-(1,3,4-thiadiazol-2-yl)thiophene-3-carbohydrazide (20)**

Yield, 73%; m.p. 218-220 °C; IR (KBr): ν_max_, cm^-1^: 3410 (NH_2_), 3225 (NH), 1725 (CO), 1677 (amidic CO), 1621 (C=N), 1600 (C=C); ^1^H-NMR (DMSO-*d_6_*) *δ* ppm: 2.25 (s, 3H, CH_3_), 5.64 (s, 2H, NH_2_), 6.94-7.19 (m, 1H, Ar-H), 7.57 (s, 4H, Ar-H), 8.32 (s, 1H, NH), 9.31 (s, 1H, thiadiazole-CH), 10.39 (s, 1H, NH), 10.82 (s, 1H, NH) **(Figure S49)**; ^13^C-NMR (DMSO-*d_6_*) *δ* ppm: 26.1, 103.3, 117.2 (2C), 121.9, 126.0, 129.6 (2C), 140.6, 146.7, 152.3, 164.6, 169.7, 173.5, 190.7 **(Figure S50)**. MS (m/z, %): 374 (M^+^, 67) **(Figure S51)**. Anal. Calced for C_15_H_14_N_6_O_2_S_2_ (374.44): C, 48.12; H, 3.77; N, 22.44%. Found: C, 48.05; H, 3.73; N, 22.37%.

**4-Amino-5-cyano-2-(phenylamino)-N'-(1,3,4-thiadiazol-2-yl)thiophene-3-carbohydrazide (22)**

Yield, 71%; m.p. 233-235 °C; IR (KBr): ν_max_, cm^-1^: 3415 (NH_2_), 3240 (NH), 2218 (CN), 1682 (amidic CO), 1623 (C=N), 1600 (C=C); ^1^H-NMR (DMSO-*d_6_*) *δ* ppm: 5.34 (s, 2H, NH_2_), 6.90-7.07 (m, 5H, Ar-H), 8.39 (s, 1H, NH), 9.30 (s, 1H, thiadiazole-CH), 10.29 (s, 1H, NH), 10.68 (s, 1H, NH) **(Figure S52)**; ^13^C-NMR (DMSO-*d_6_*) *δ* ppm: 84.8, 103.4, 113.1, 117.7 (2C), 122.8, 129.2 (2C), 132.4, 140.1, 152.8, 164.8, 167.8, 174.8 **(Figure S53)**. MS (m/z, %): 357 (M^+^, 66) **(Figure S54)**. Anal. Calced for C_14_H_11_N_7_OS_2_ (357.41): C, 47.05; H, 3.10; N, 27.43%. Found: C, 46.98; H, 3.02; N, 27.35%.

**Ethyl 4-(2-(1,3,4-thiadiazol-2-yl)hydrazine-1-carbonyl)-3-amino-5-(phenylamino)thiophene-2-carboxylate (24)**

Yield, 78%; m.p. 257-259 °C; IR (KBr): ν_max_, cm^-1^: 3440 (NH_2_), 3235 (NH), 1715 (CO), 1680 (amidic CO), 1622 (C=N), 1605 (C=C); ^1^H-NMR (DMSO-*d_6_*) *δ* ppm: 1.29 (t, 3H, CH_3_), 4.28 (q, 2H, CH_2_), 5.61 (s, 2H, NH_2_), 6.91-7.41 (m, 5H, Ar-H), 8.41 (s, 1H, NH), 9.50 (s, 1H, thiadiazole-CH), 10.29 (s, 1H, NH), 10.77 (s, 1H, NH) **(Figure S55)**; ^13^C-NMR (DMSO-*d_6_*) *δ* ppm: 14.9, 60.4, 103.1, 117.5 (2C), 120.8, 122.1, 128.9, 130.1 (2C), 140.5, 152.4, 160.8, 165.0, 168.7, 173.5 **(Figure S56)**. MS (m/z, %): 404 (M^+^, 55) **(Figure S57)**. Anal. Calced for C_16_H_16_N_6_O_3_S_2_ (404.46): C, 47.51; H, 3.99; N, 20.78%. Found: C, 47.47; H, 3.91; N, 20.72%.

**References**

1. Grant, A. M., S. V. Krees, A. B. Mauger, W. J. Rzeszotarski, and F. W. Wolff. "Hypotensive thiadiazoles." *Journal of Medicinal Chemistry* 15, no. 10 (1972): 1082-1084.


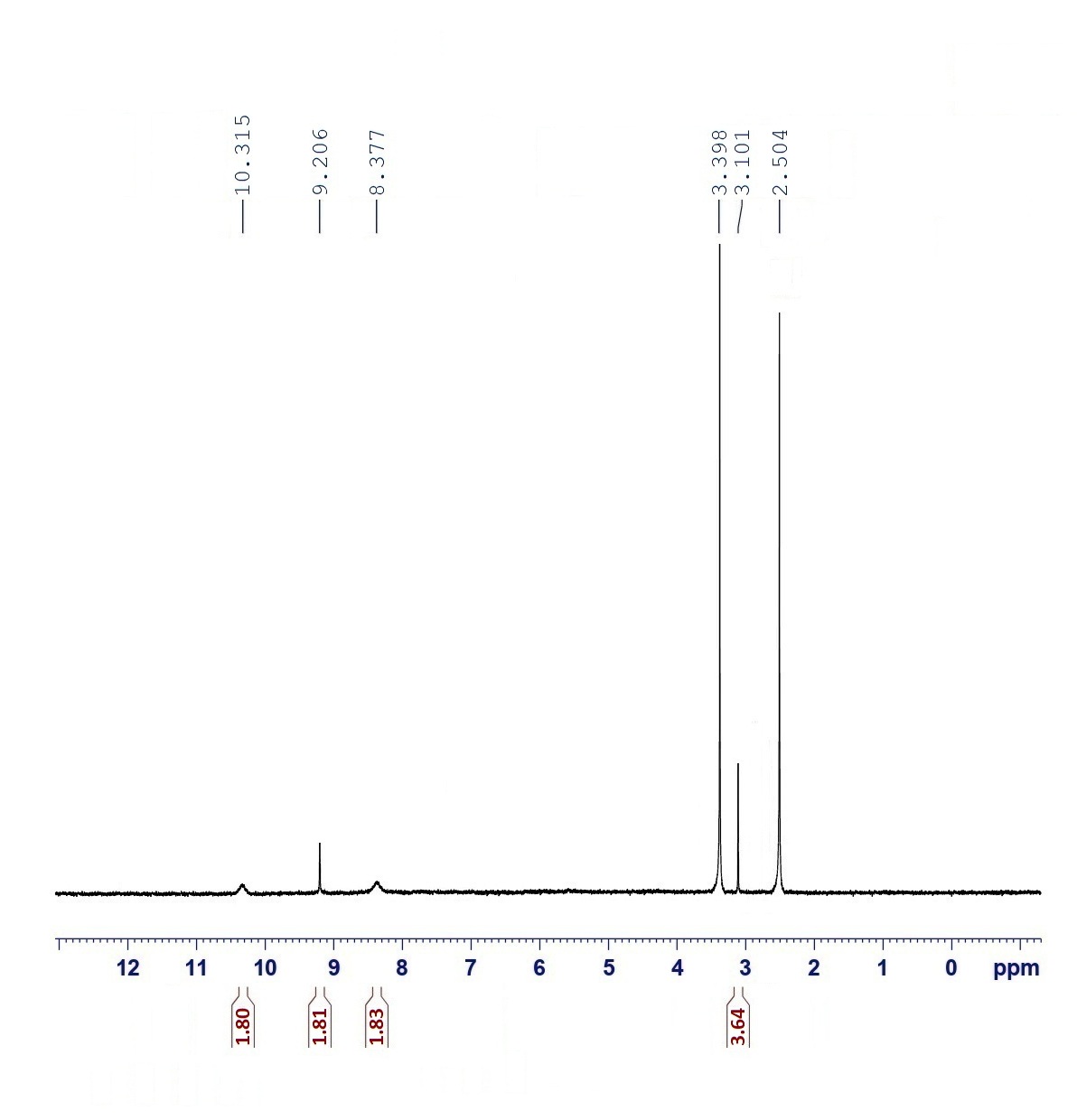

**Figure S1.** ^1^H-NMR of compound **1**


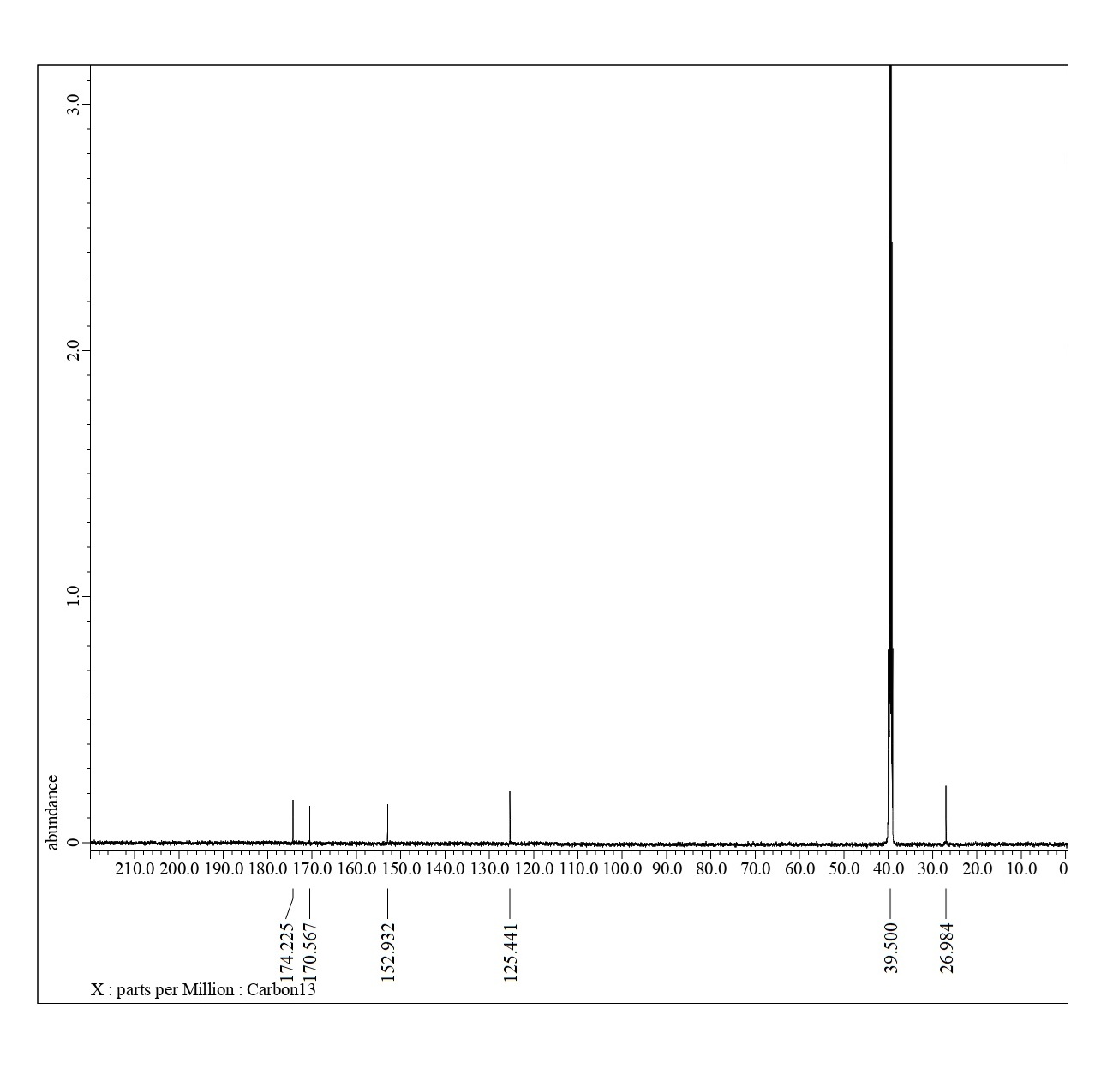

**Figure S2.** ^13^C-NMR of compound **1**

**
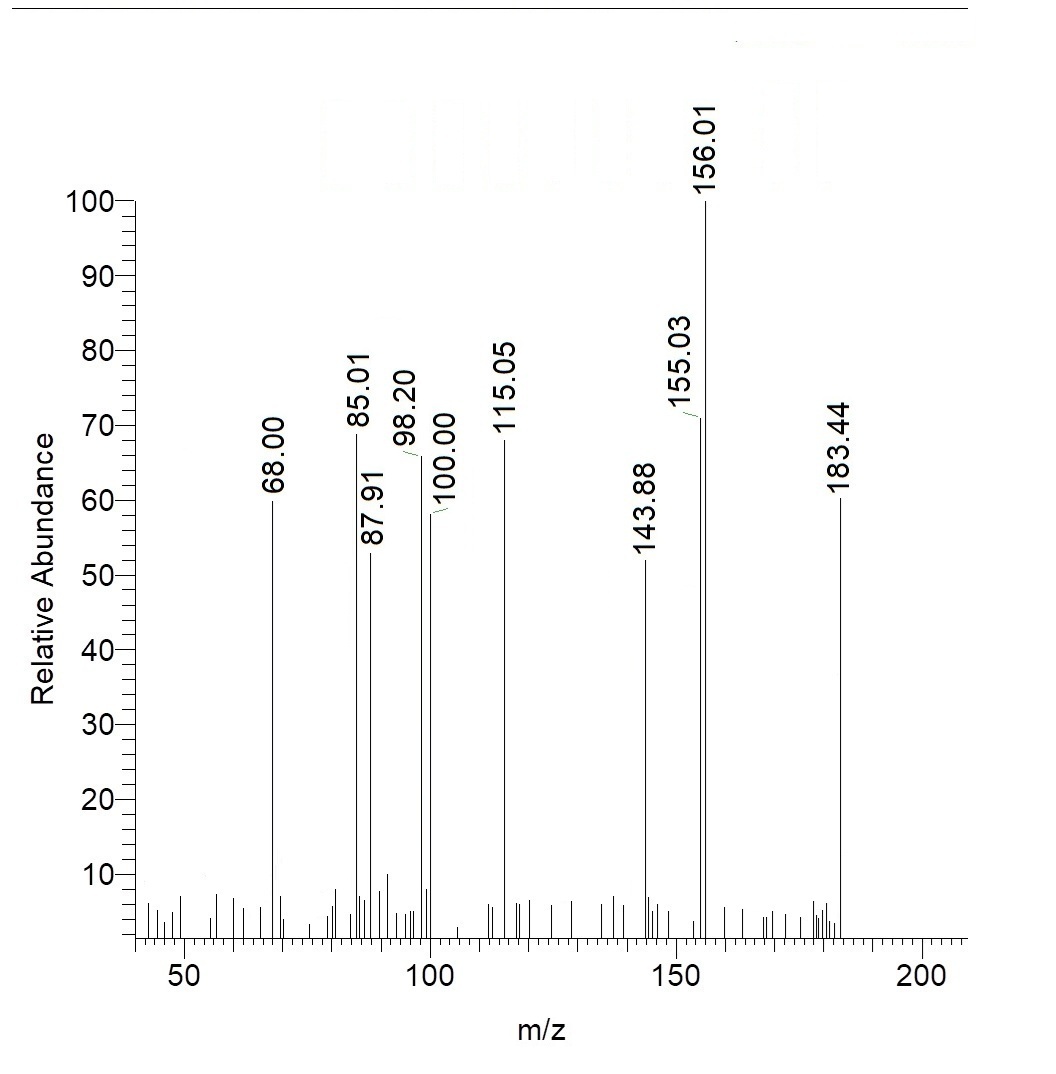
**

**Figure S3.** Mass spectrum of compound **1**

**
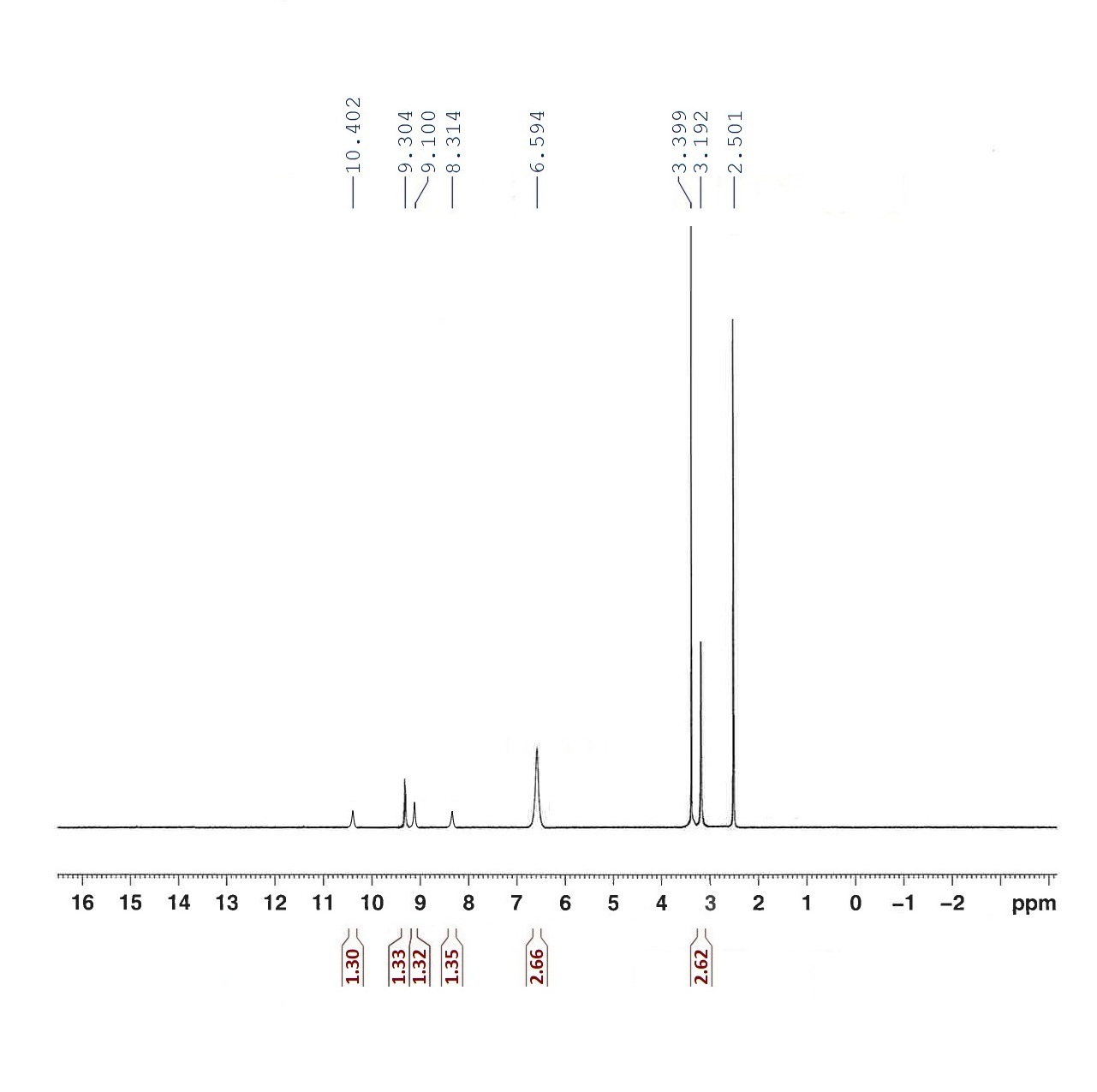
**

**Figure S4.** ^1^H-NMR of compound **2**


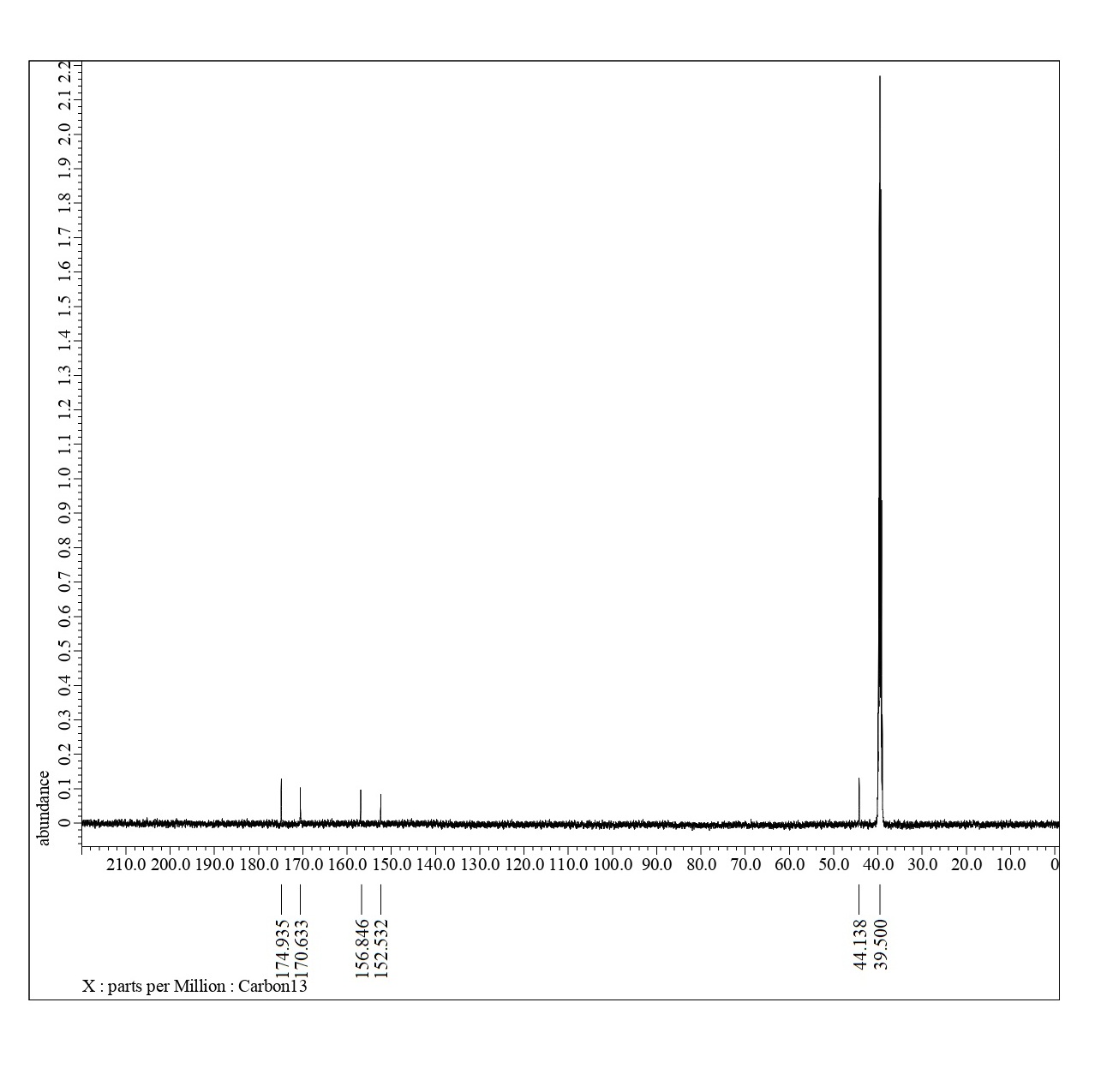

**Figure S5.** ^13^C-NMR of compound **2**

**
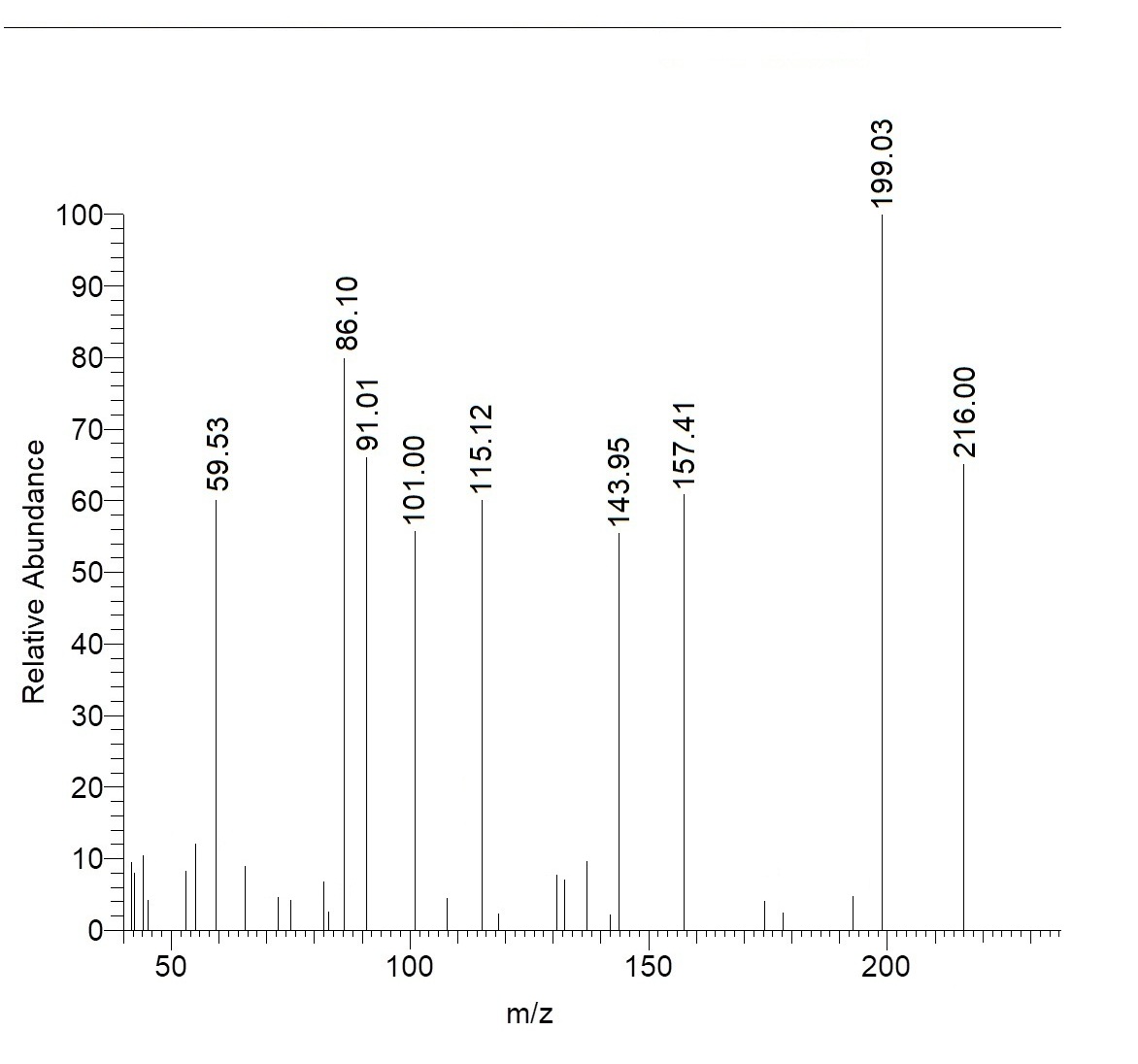
**

**Figure S6.** Mass spectrum of compound **2**

**
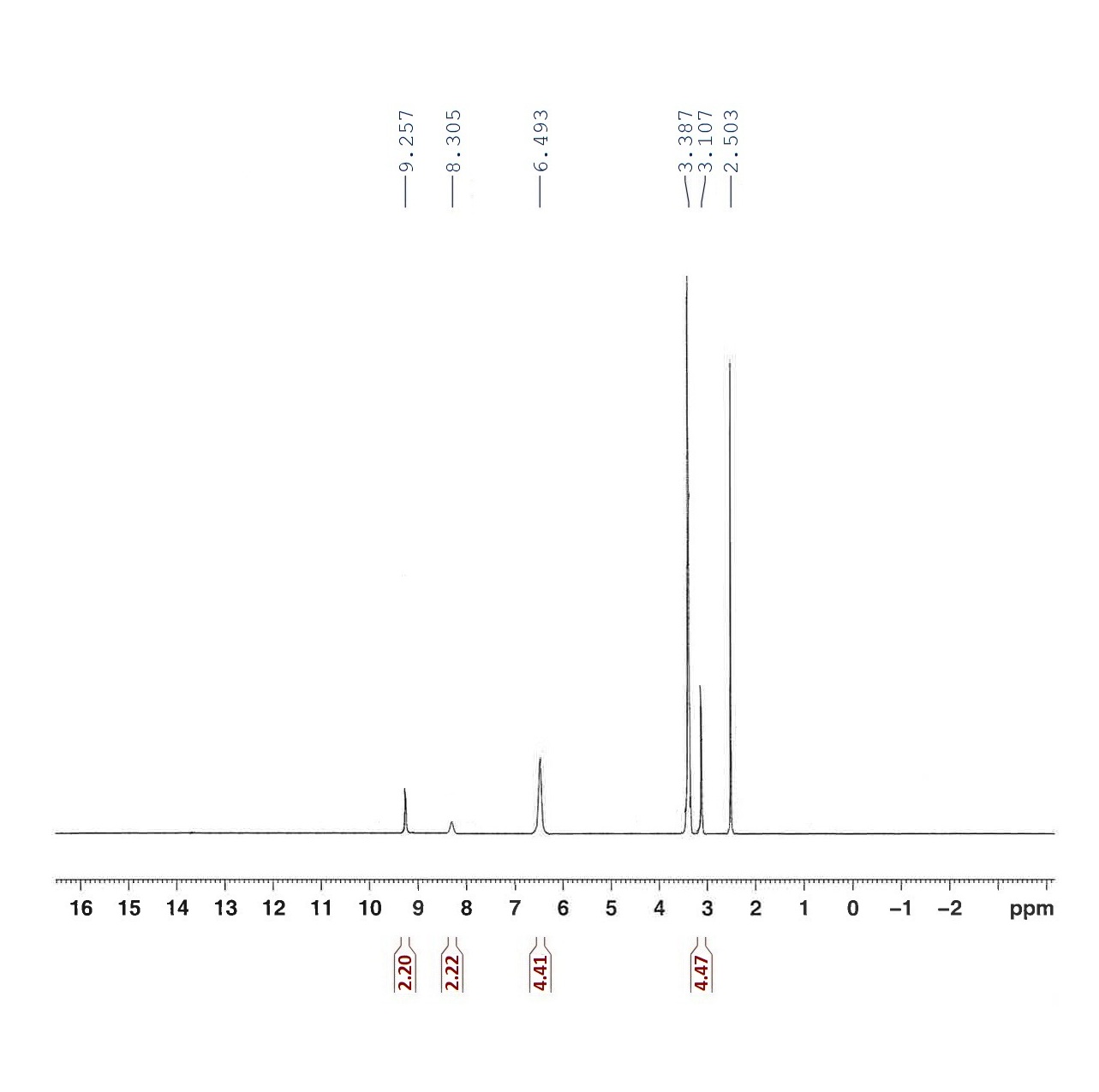
**

**Figure S7.** ^1^H-NMR of compound **5**


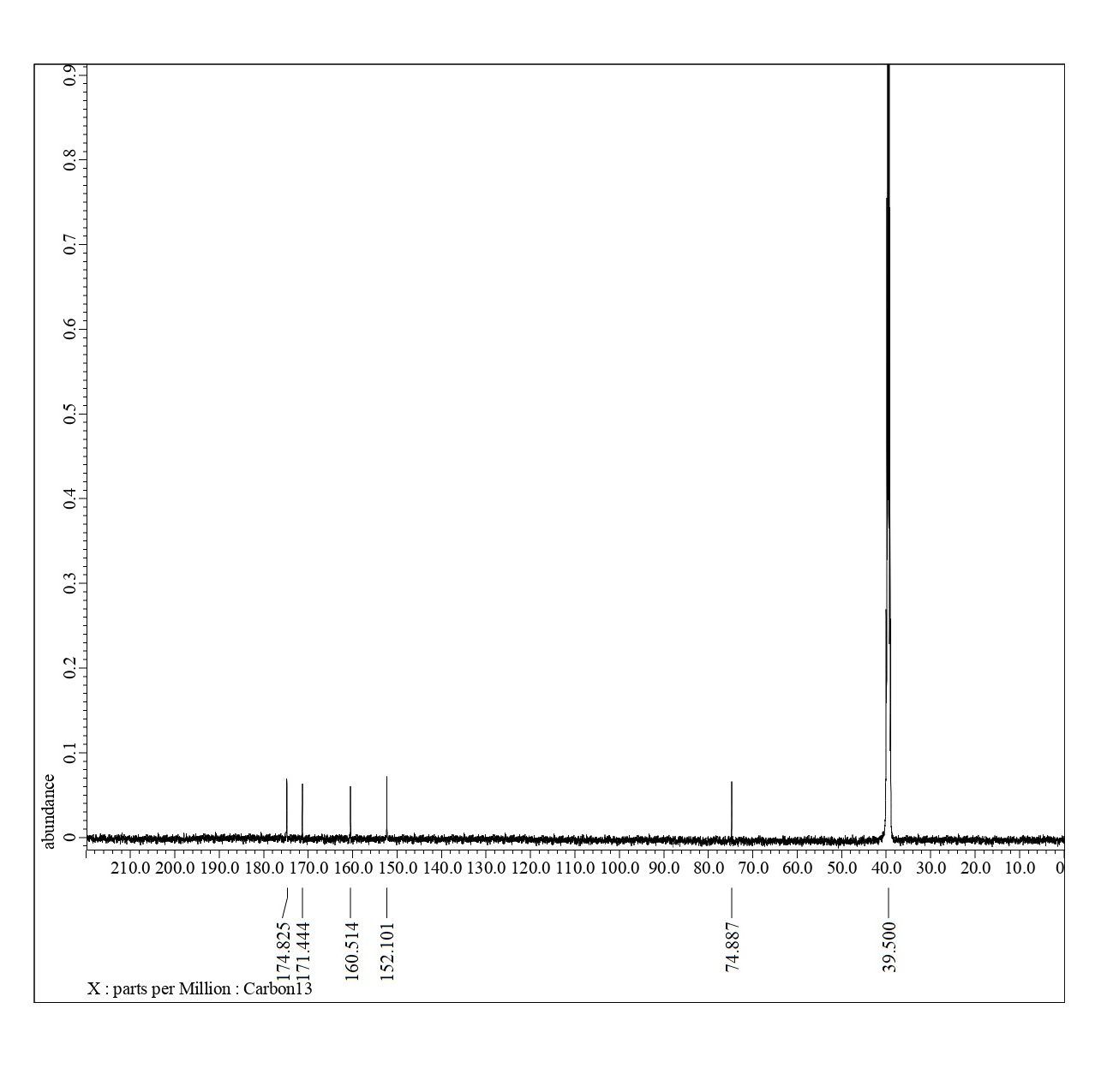

**Figure S8.** ^13^C-NMR of compound **5**

**
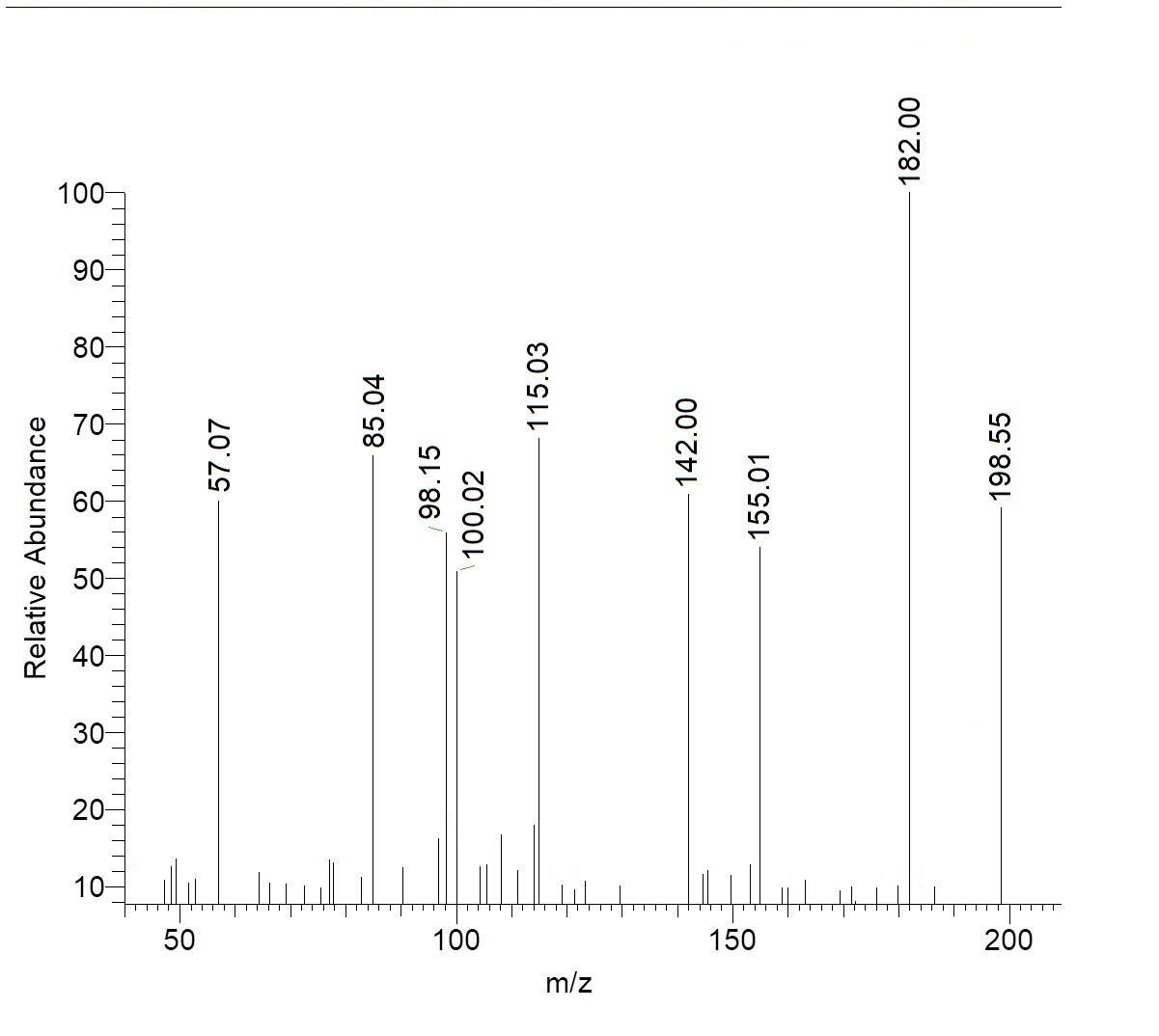
**

**Figure S9.** Mass spectrum of compound **5**

**
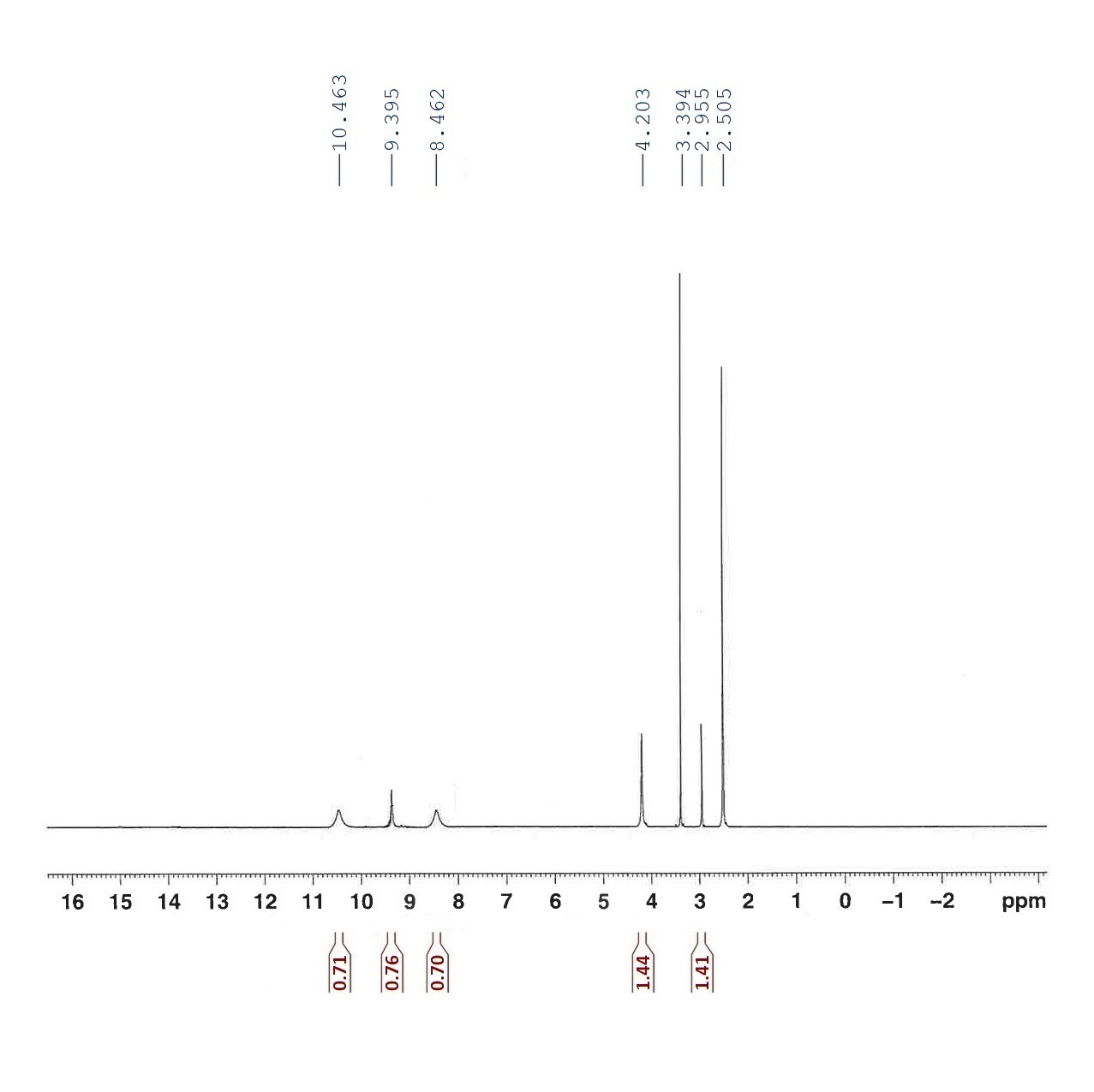
**

**Figure S10.** ^1^H-NMR of compound **7**


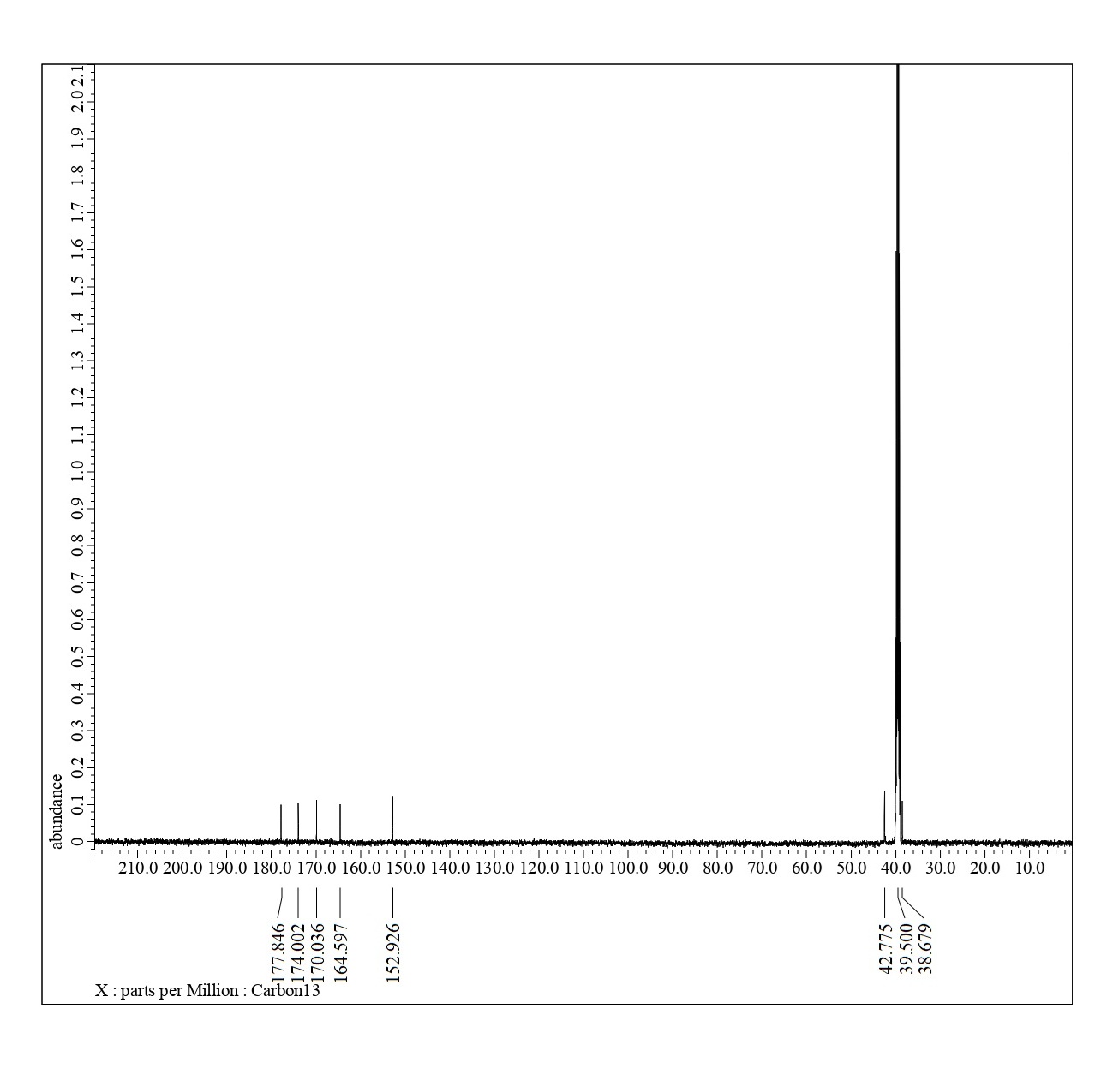

**Figure S11.** ^13^C-NMR of compound **7**

**
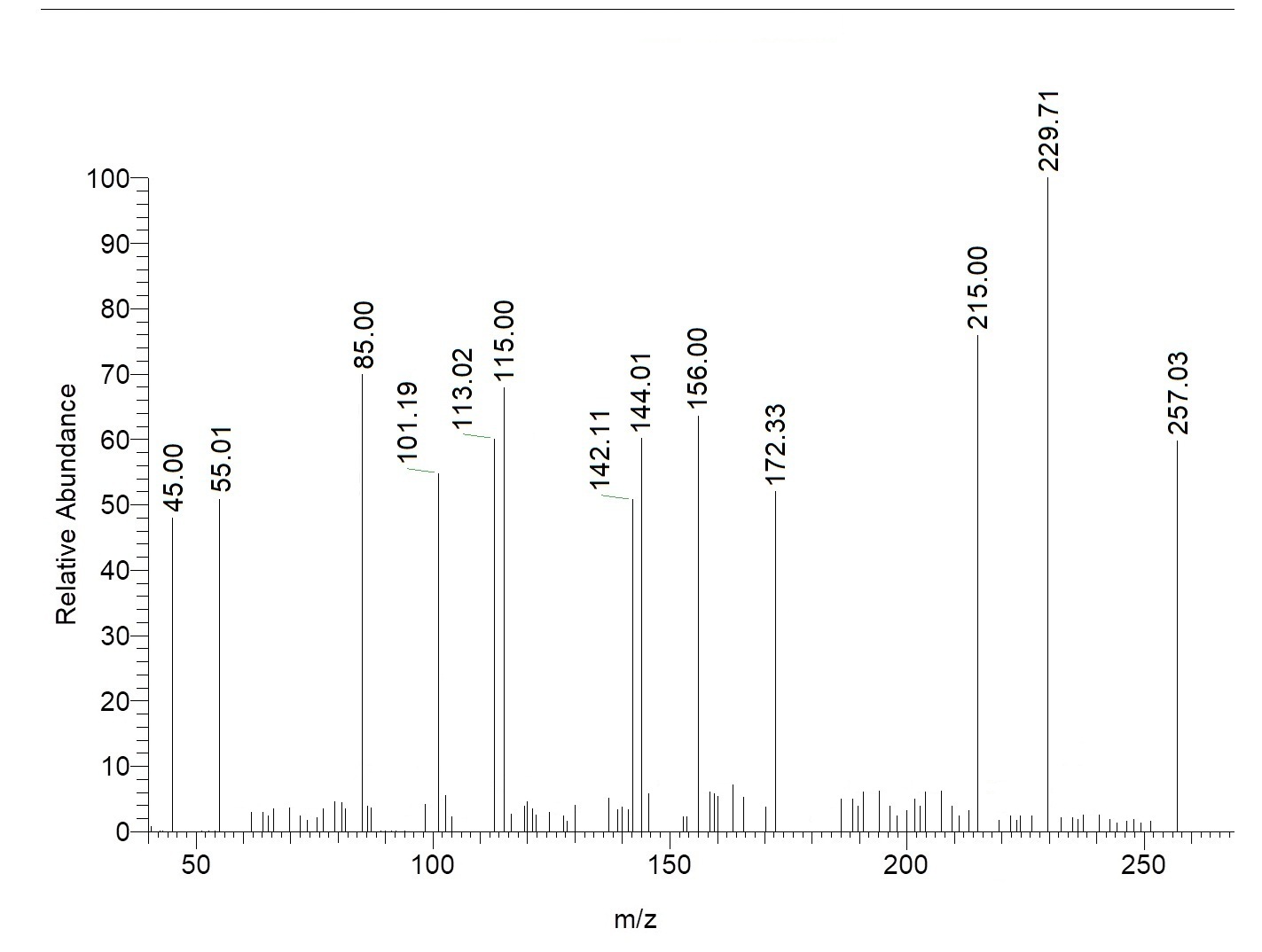
**

**Figure S12.** Mass spectrum of compound **7**

**
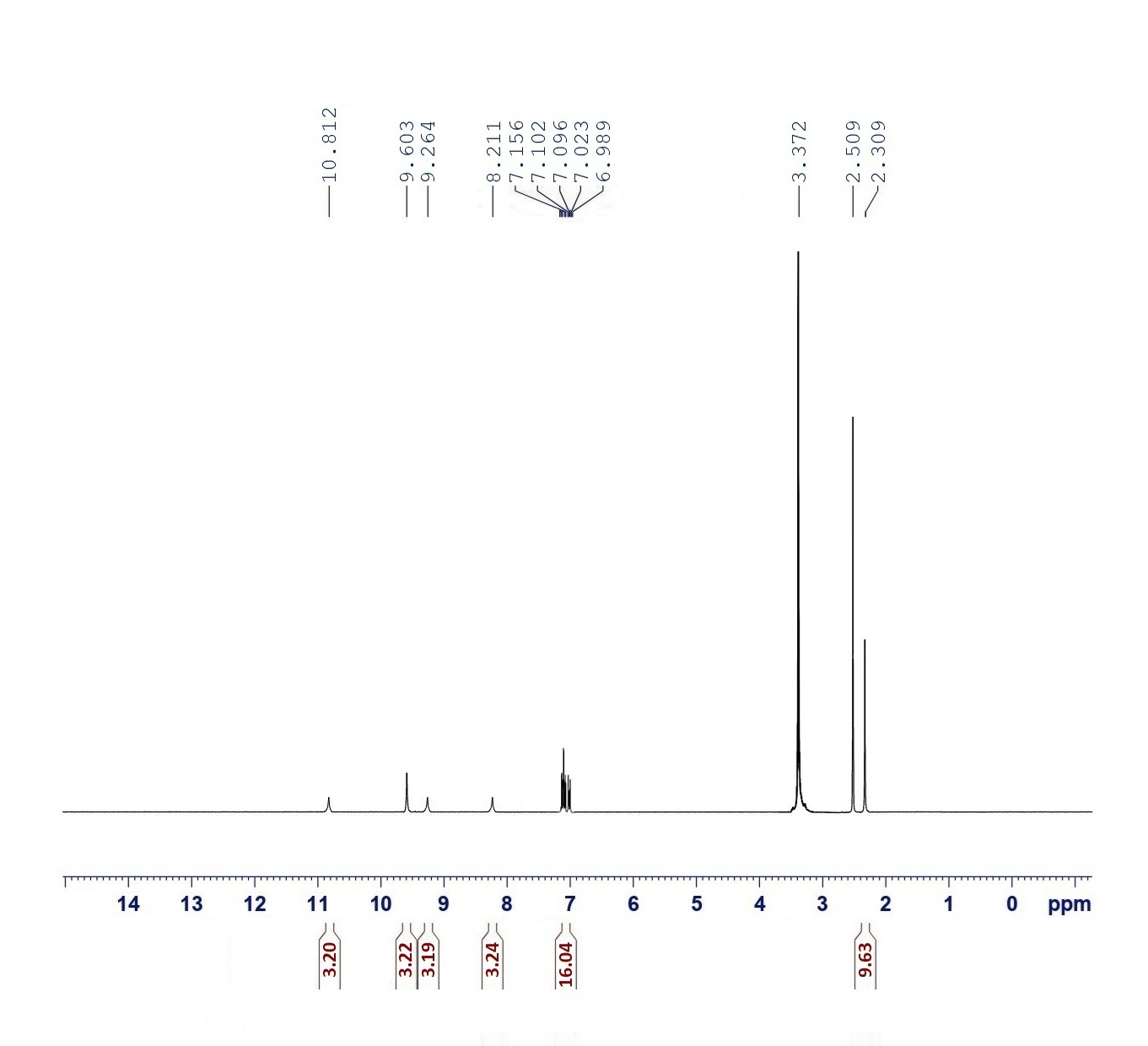
**

**Figure S13.** ^1^H-NMR of compound **9**


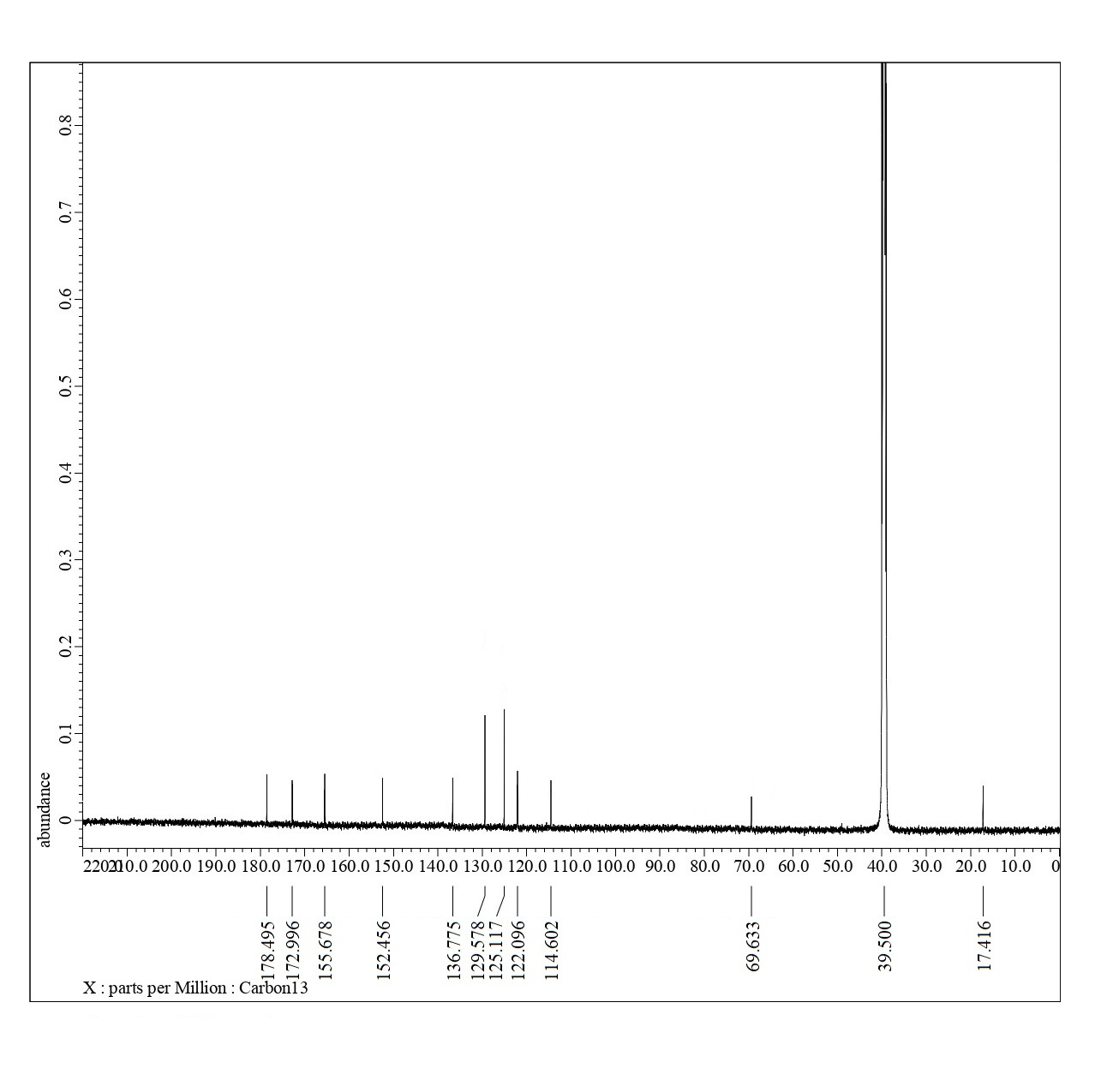

**Figure S14.** ^13^C-NMR of compound **9**

**
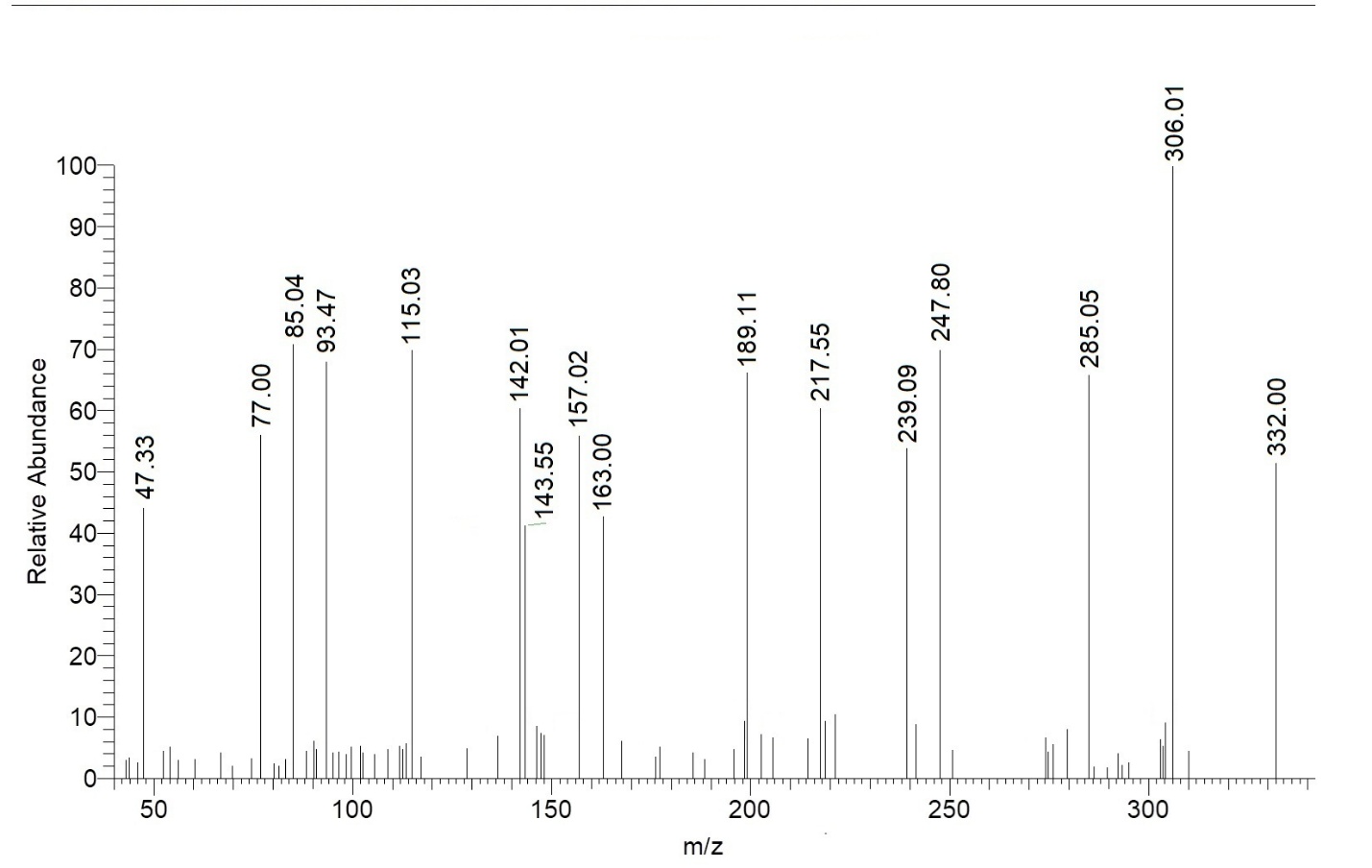
**

**Figure S15.** Mass spectrum of compound **9**

**
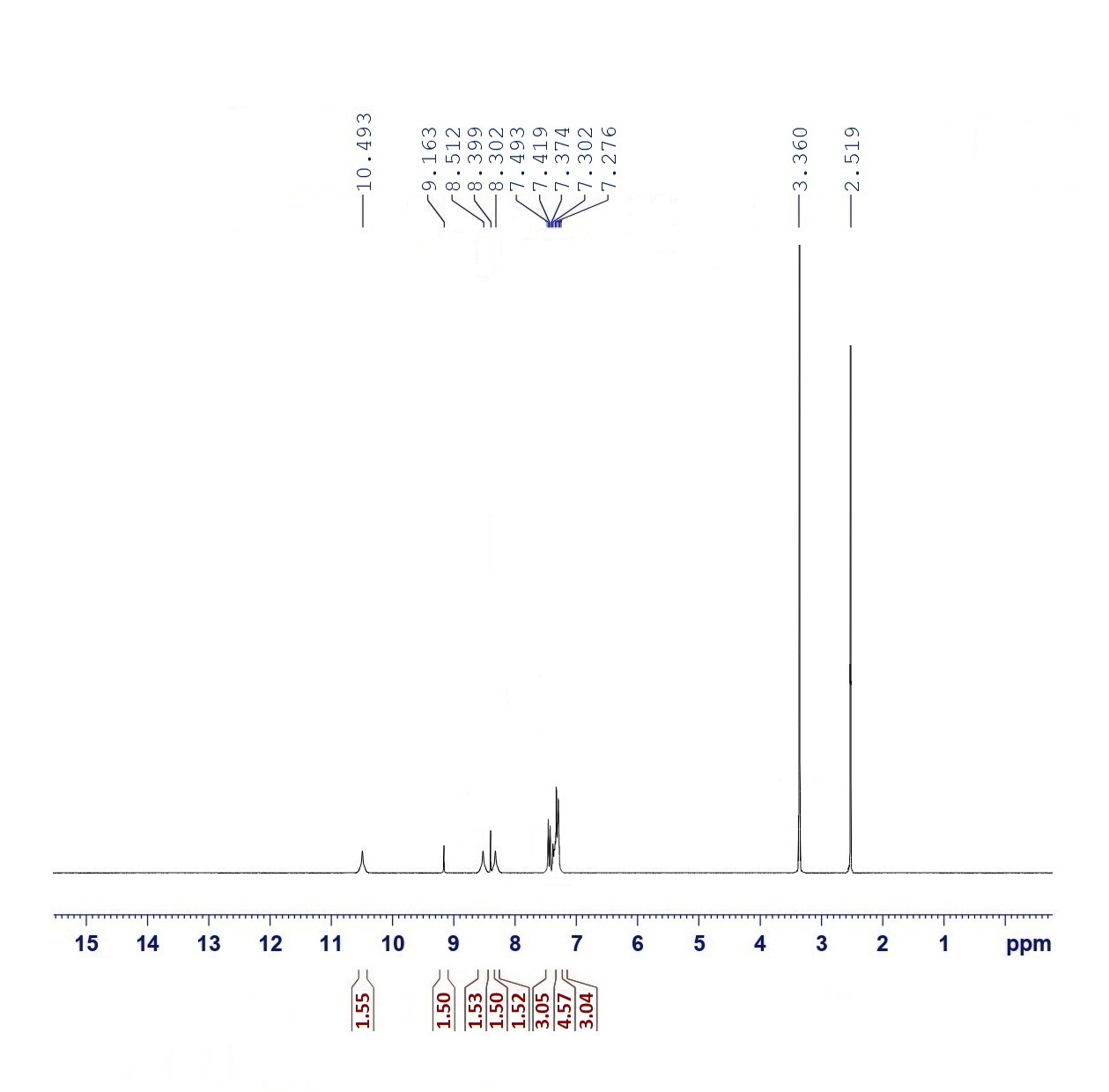
**

**Figure S16.** ^1^H-NMR of compound **10**


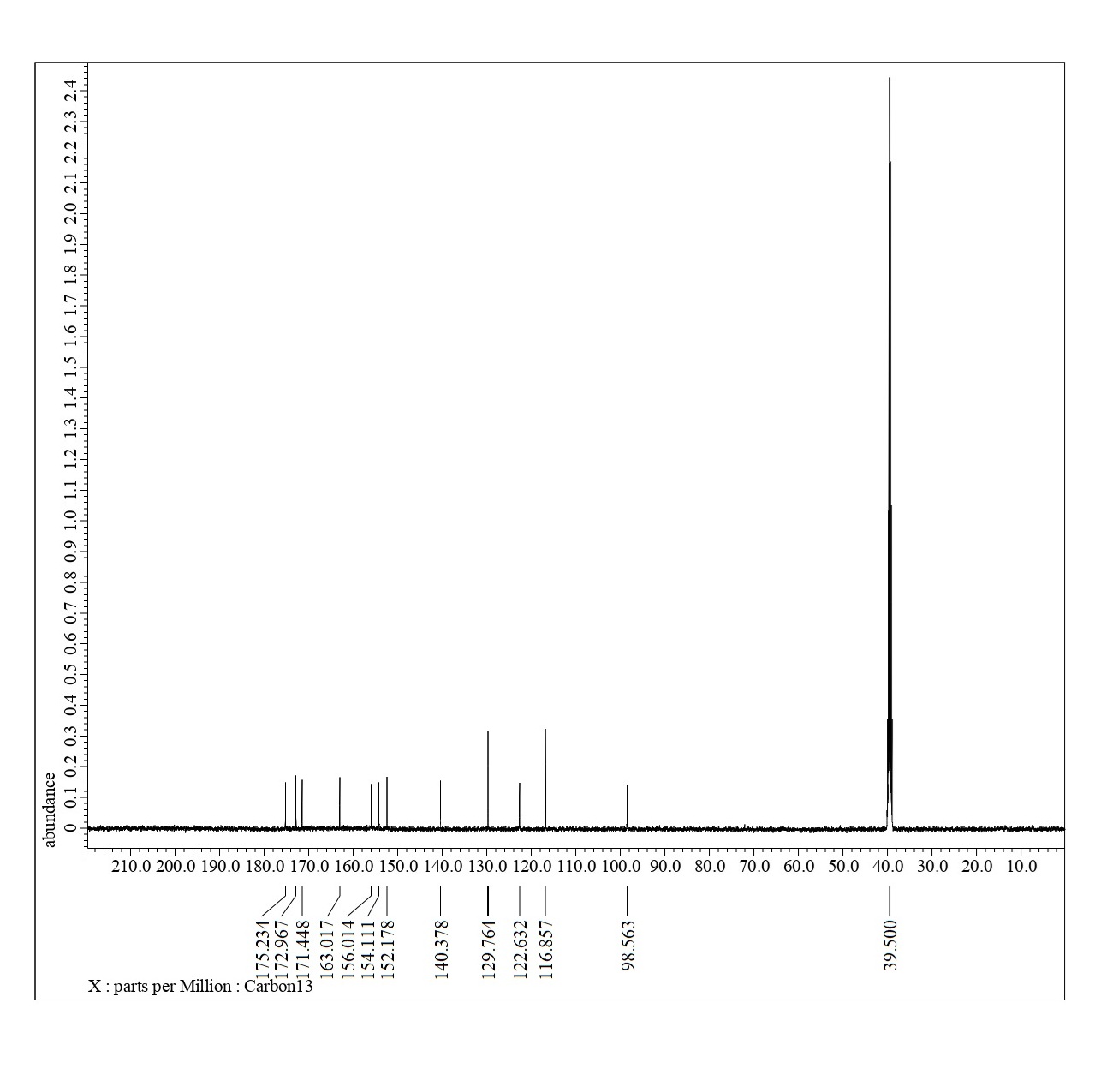

**Figure S17.** ^13^C-NMR of compound **10**

**
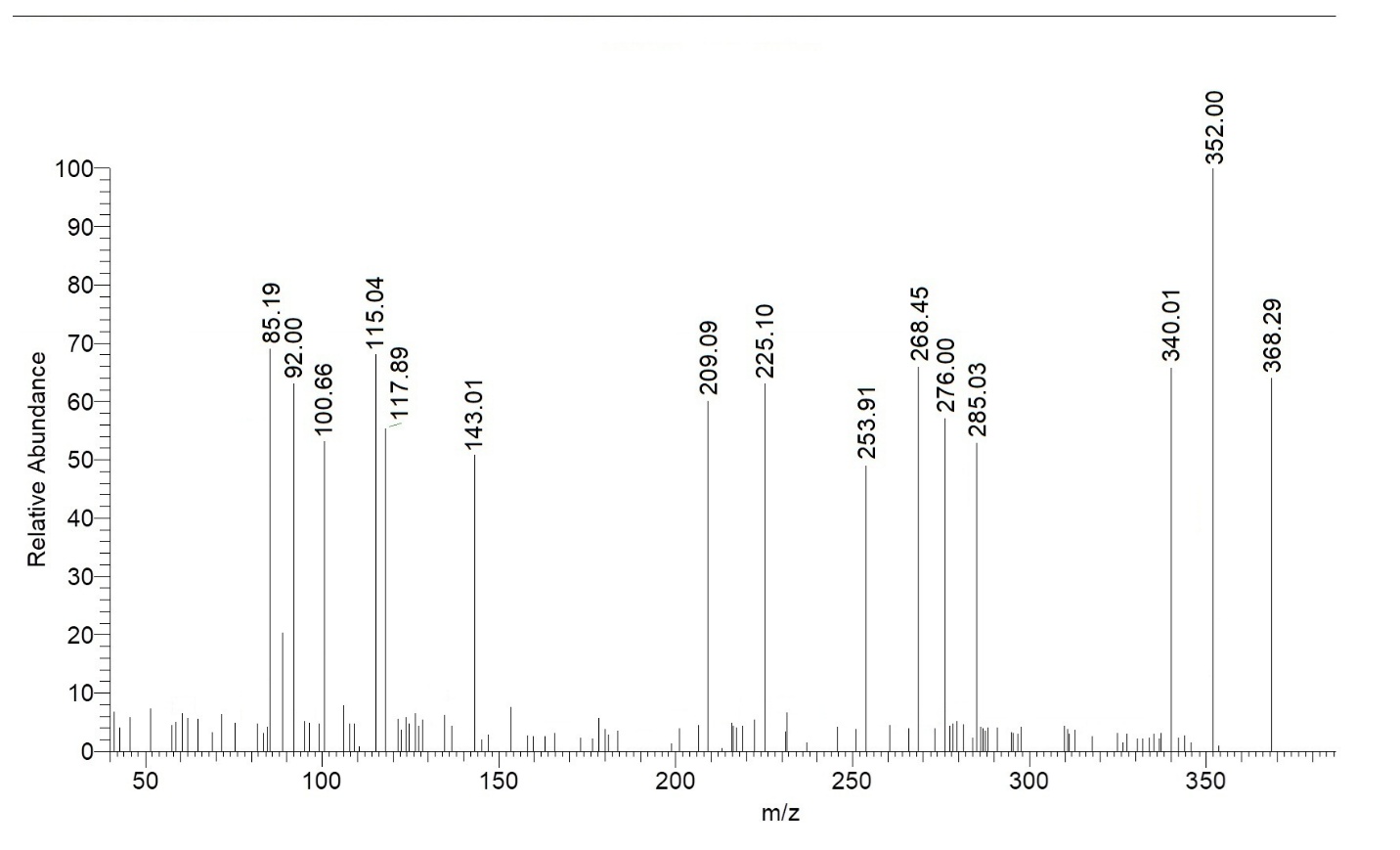
**

**Figure S18.** Mass spectrum of compound **10**

**
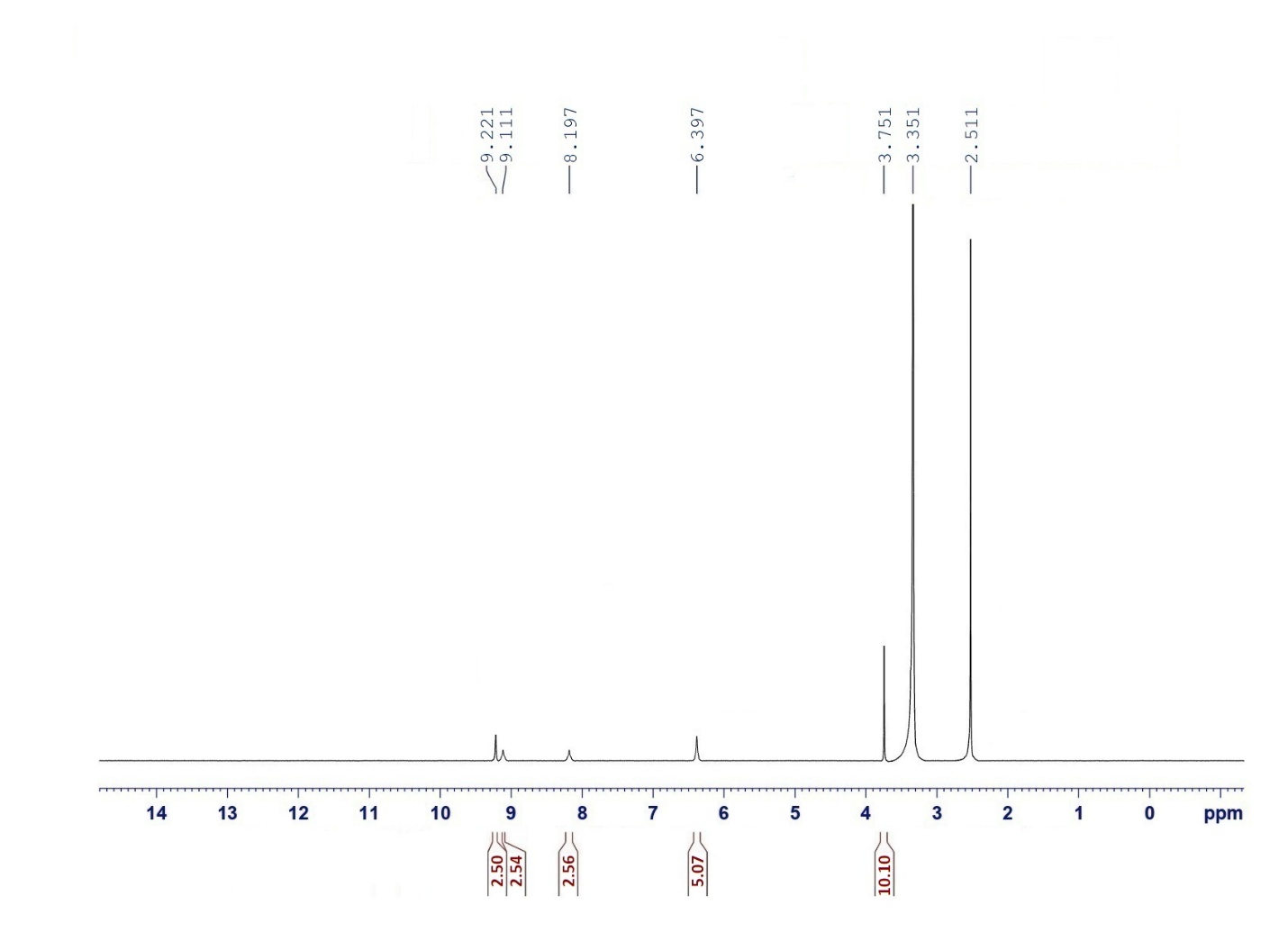
**

**Figure S19.** ^1^H-NMR of compound **11**


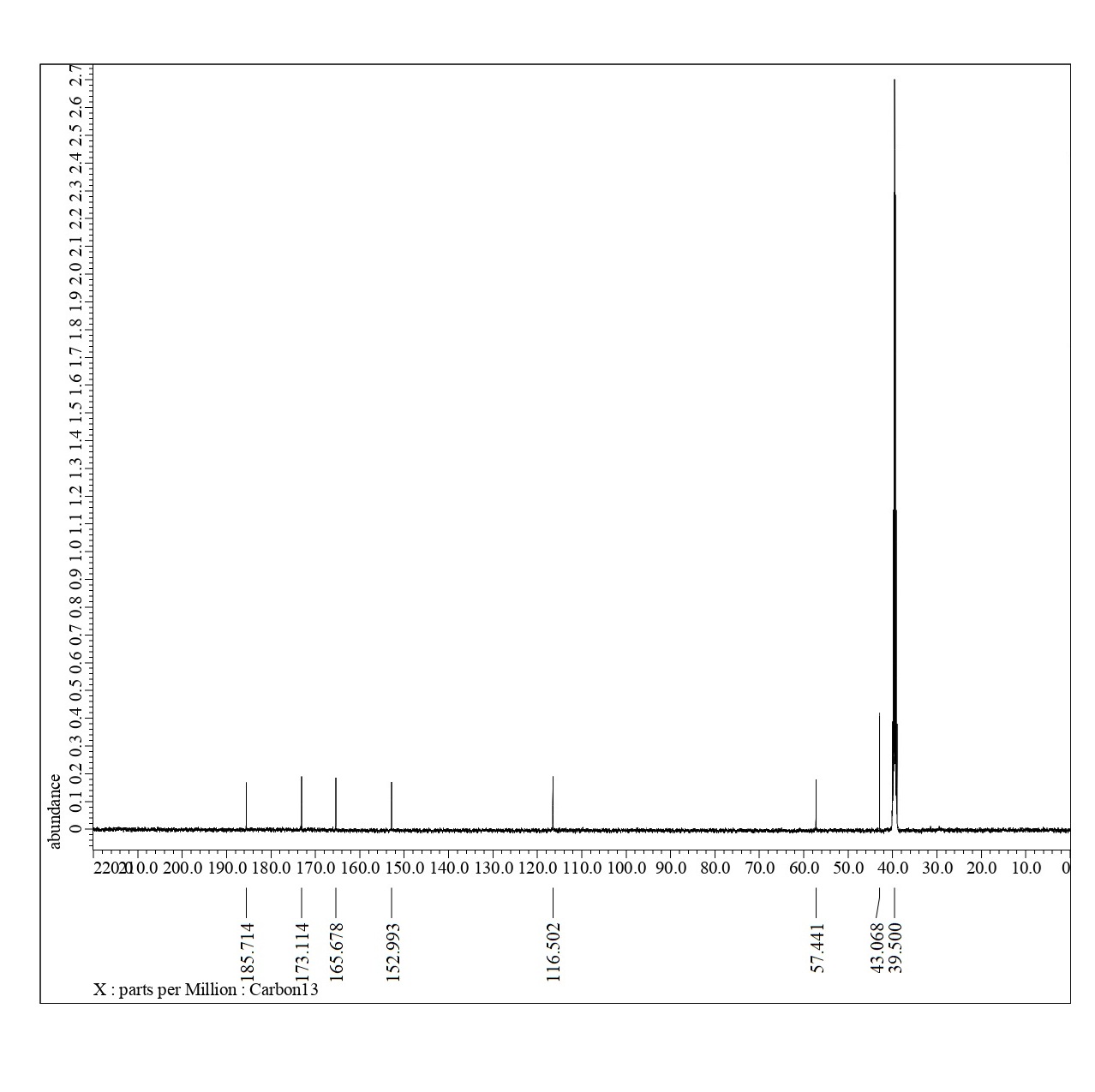

**Figure S20.** ^13^C-NMR of compound **11**

**
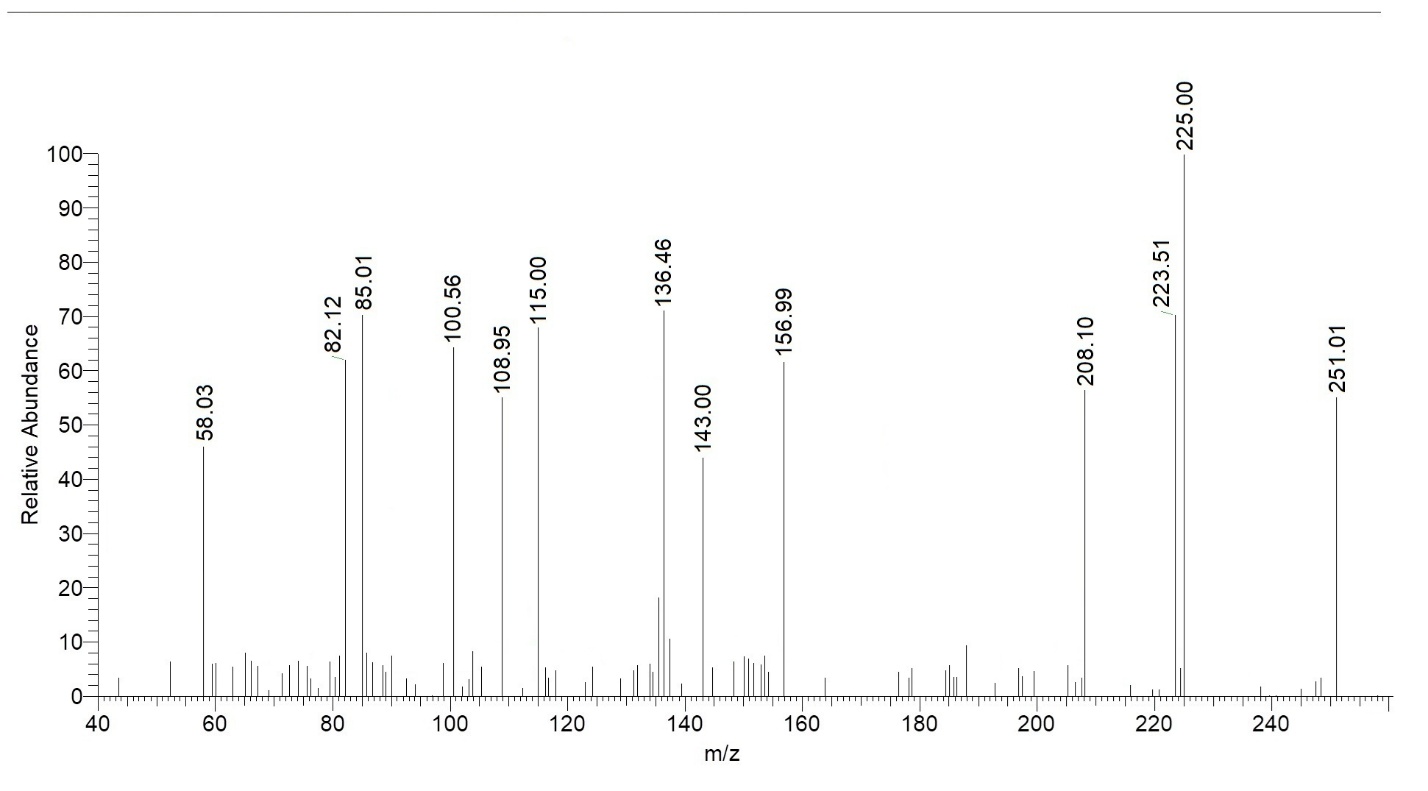
**

**Figure S21.** Mass spectrum of compound **11**

**
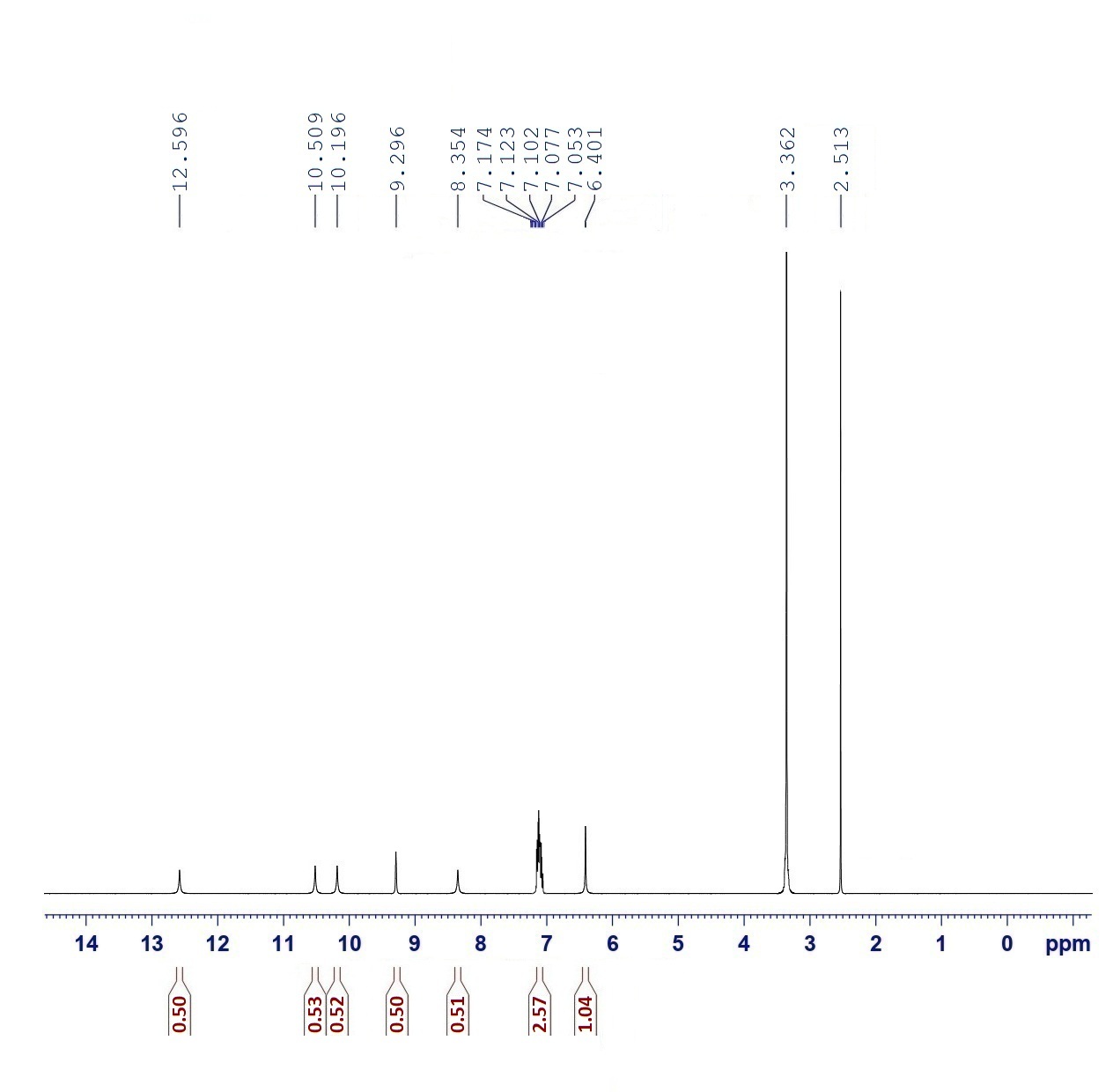
**

**Figure S22.** ^1^H-NMR of compound **12**


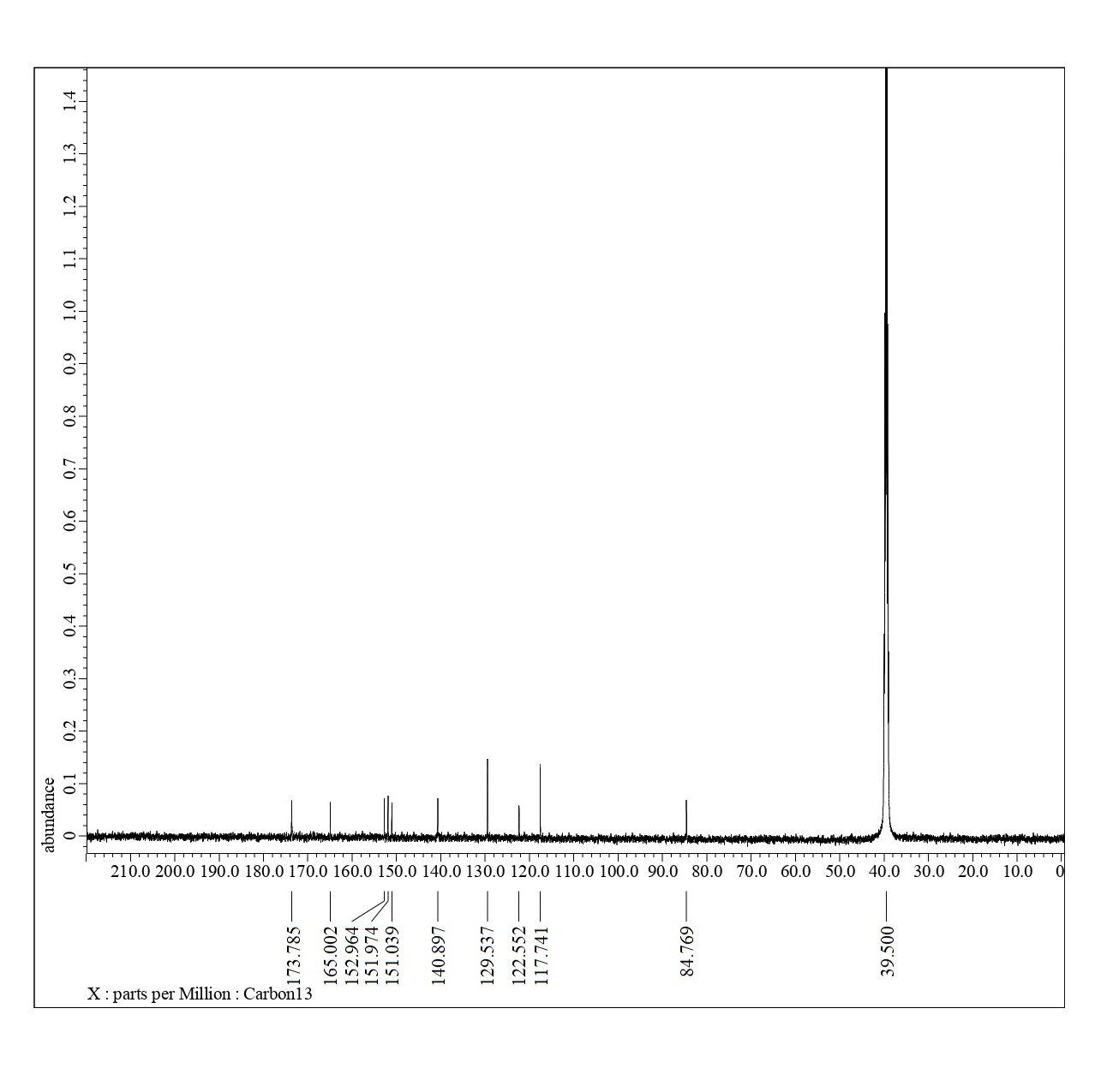

**Figure S23.** ^13^C-NMR of compound **12**

**
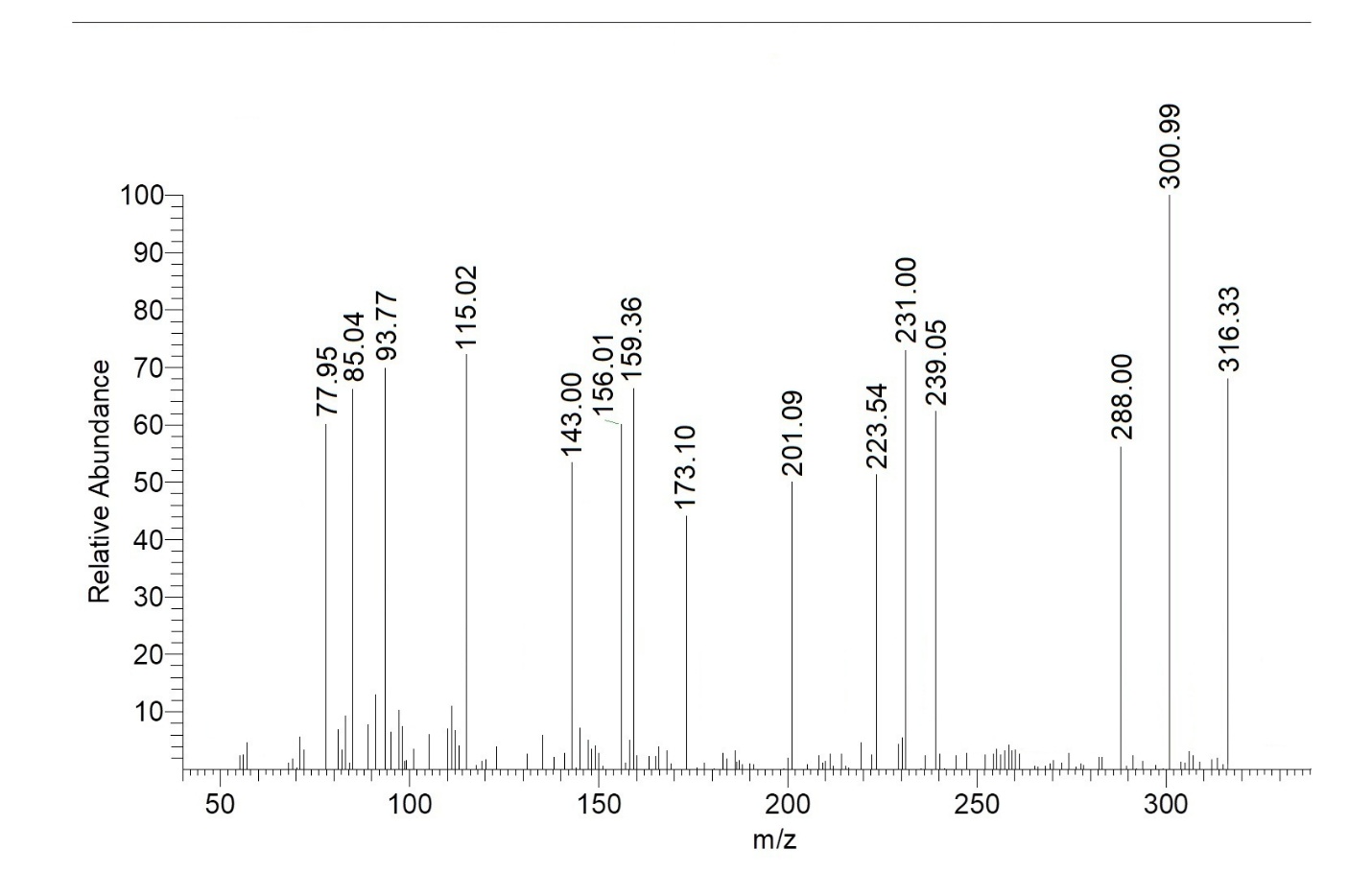
**

**Figure S24.** Mass spectrum of compound **12**

**
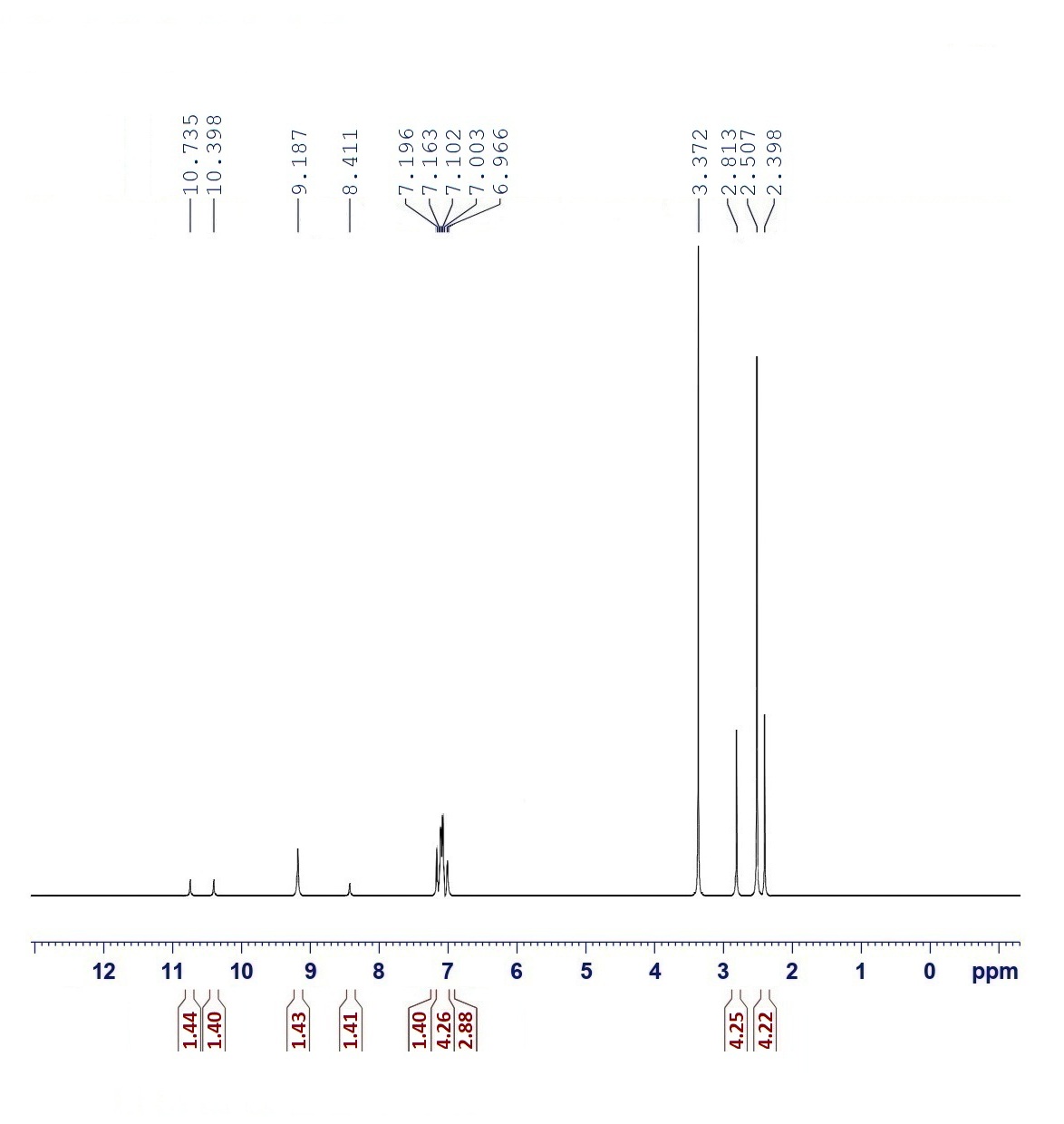
**

**Figure S25.** ^1^H-NMR of compound **13**


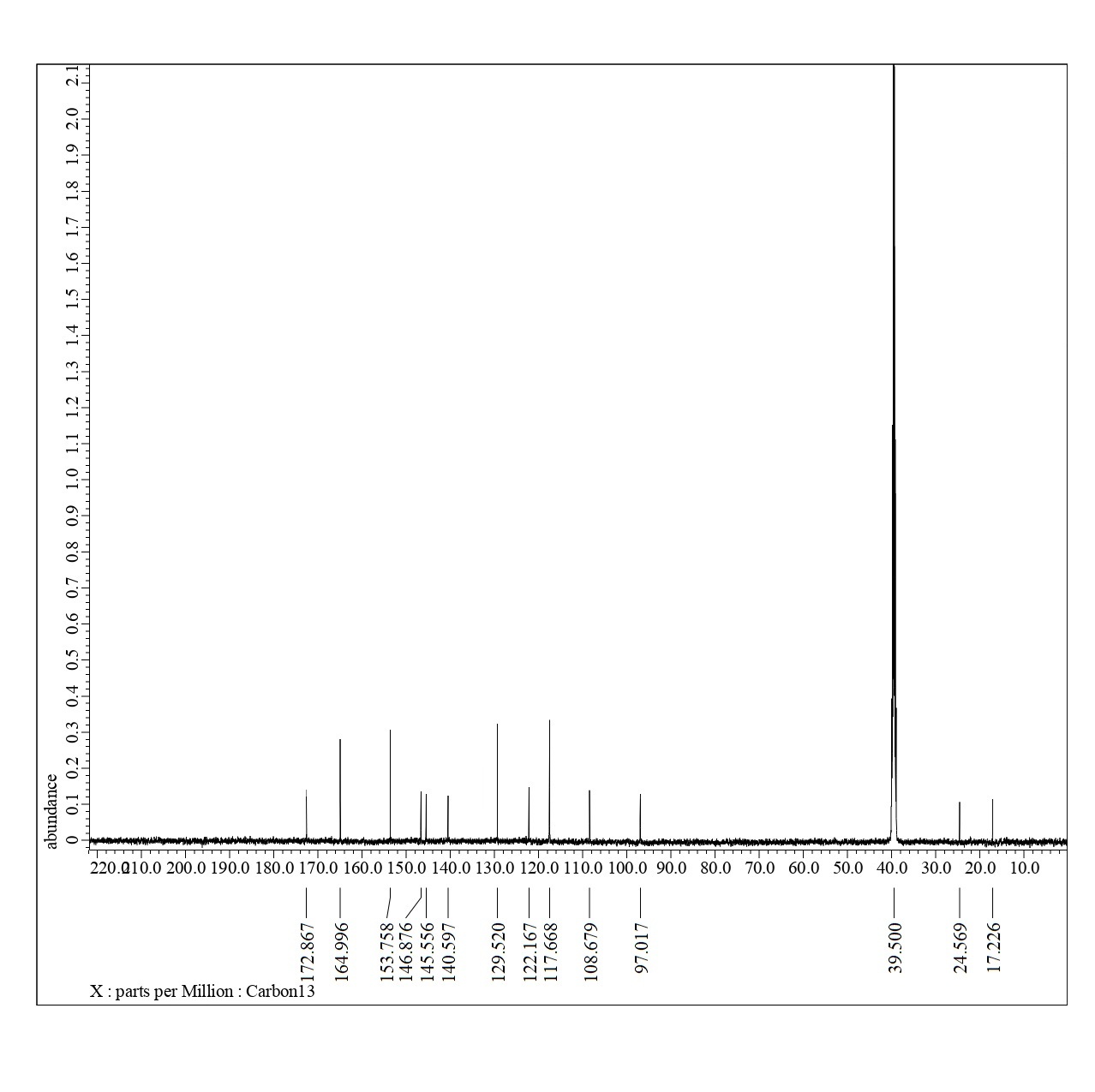

**Figure S26.** ^13^C-NMR of compound **13**

**
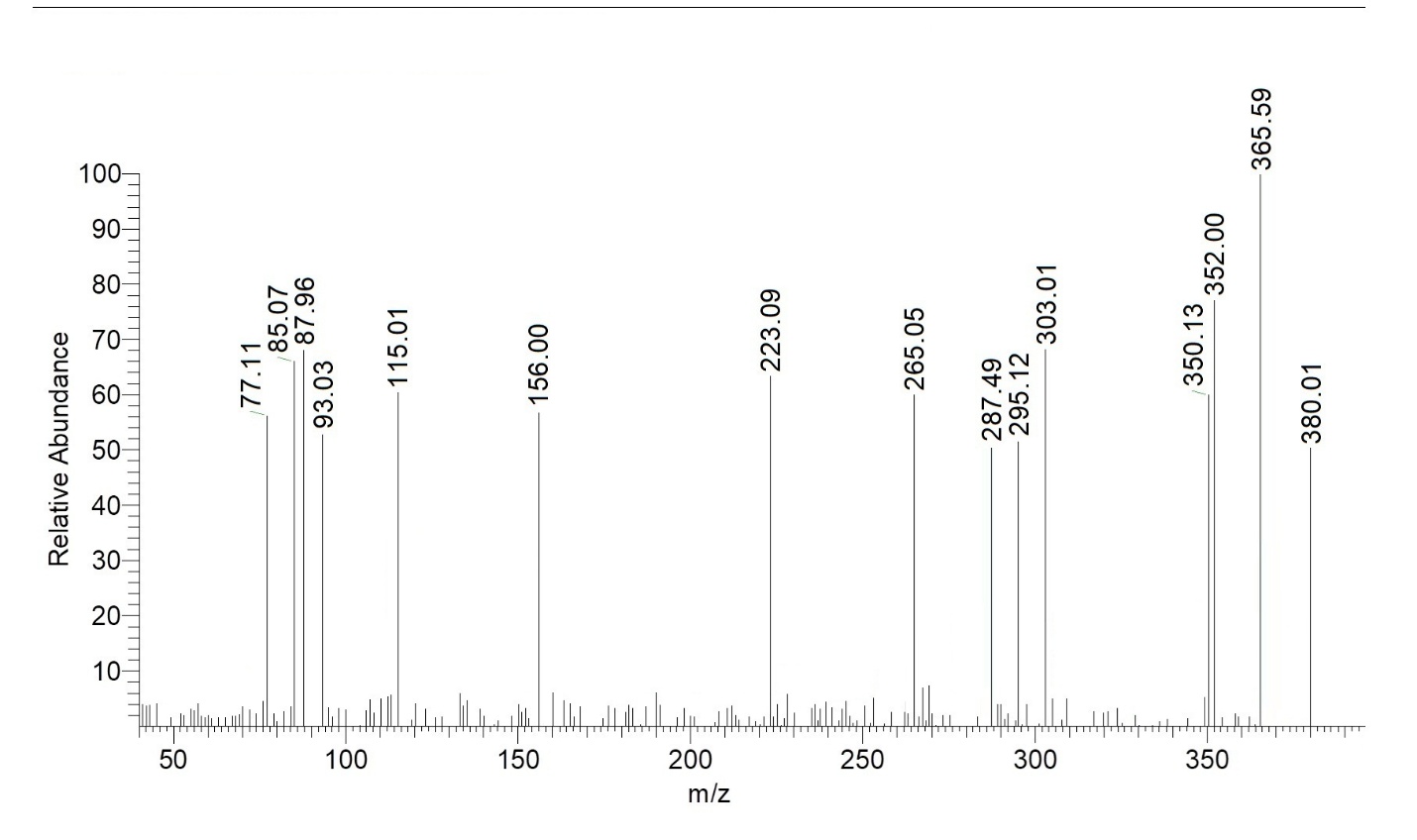
**

**Figure S27.** Mass spectrum of compound **13**

**
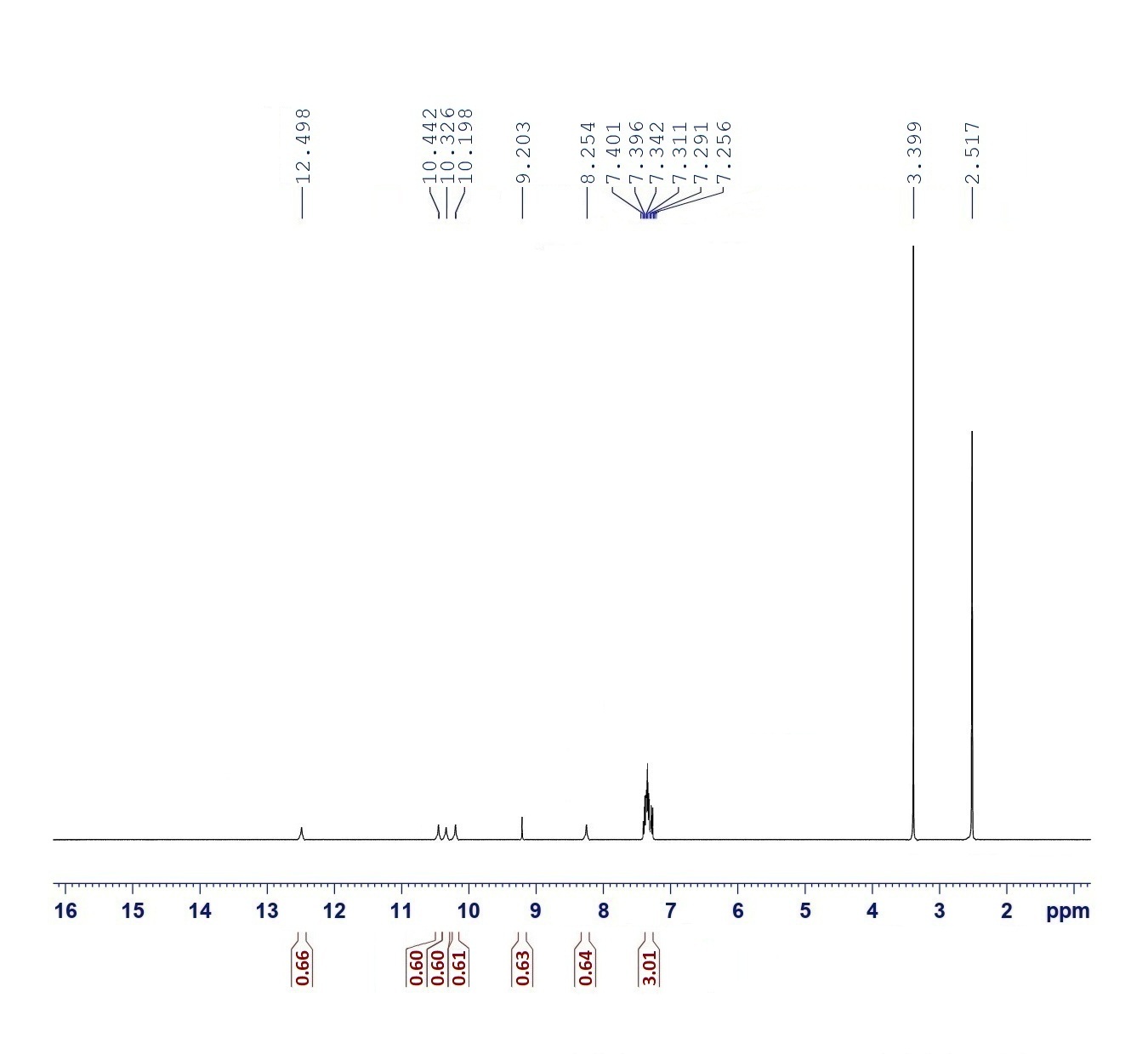
**

**Figure S28.** ^1^H-NMR of compound **15**


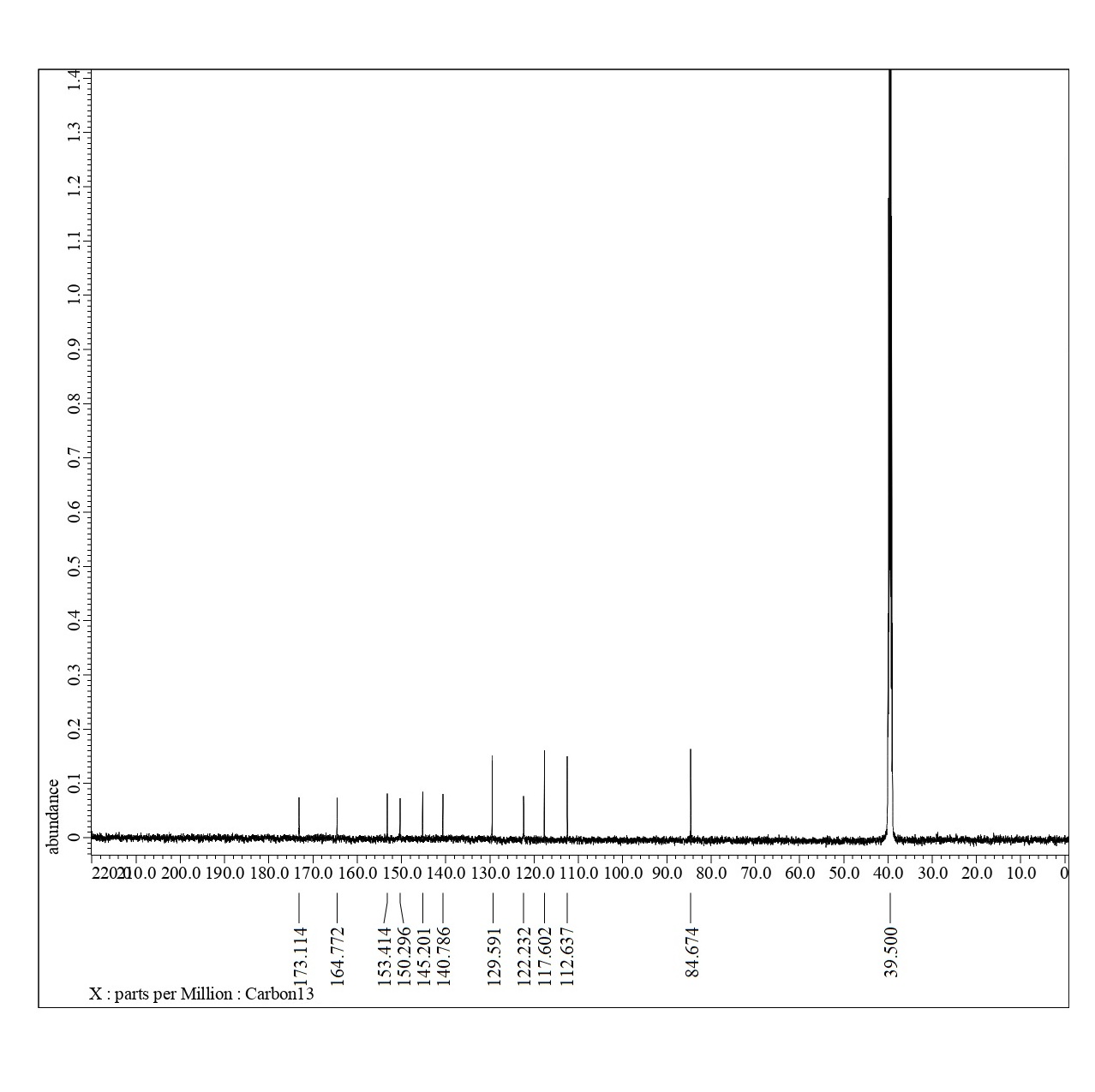

**Figure S29.** ^13^C-NMR of compound **15**

**
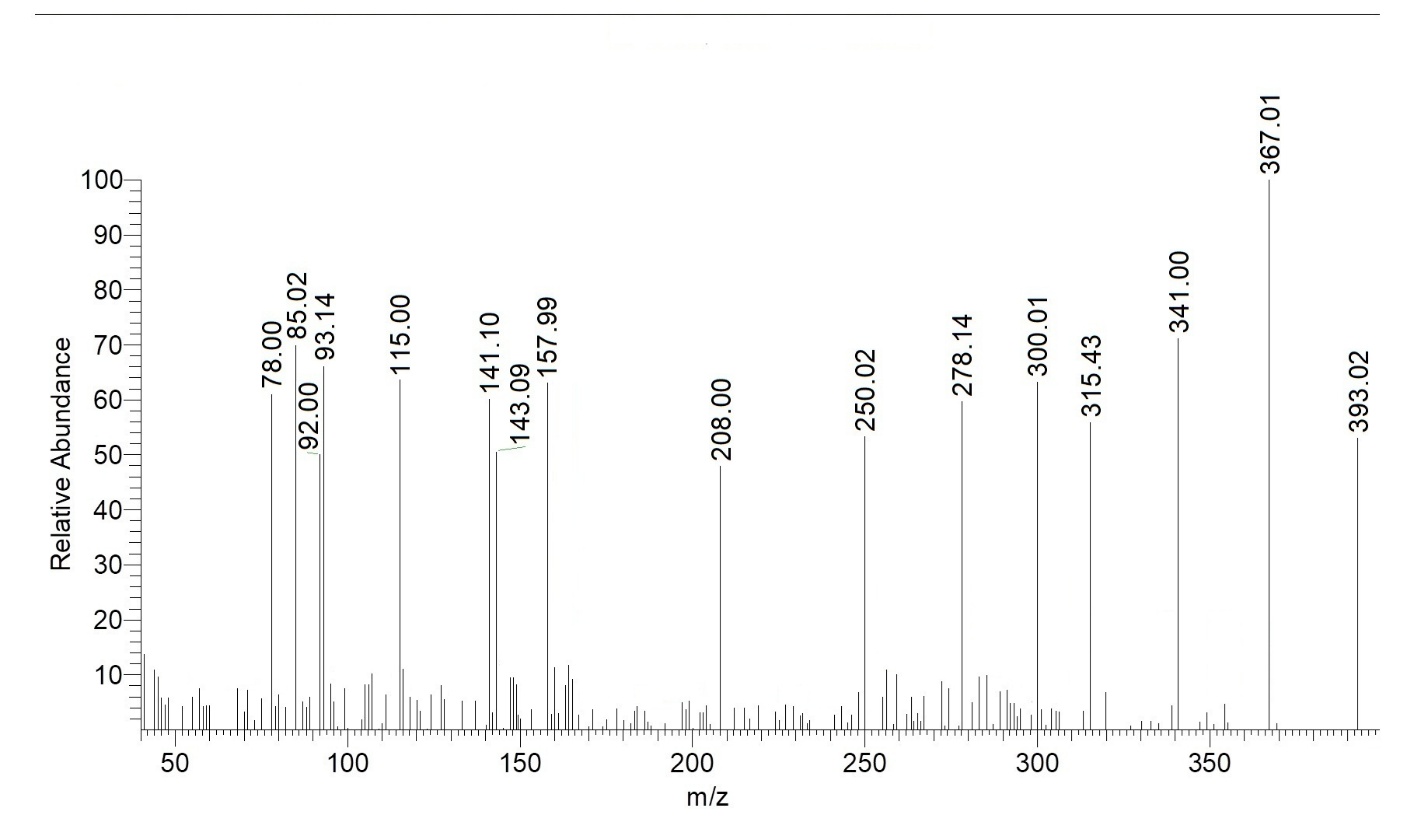
**

**Figure S30.** Mass spectrum of compound **15**

**
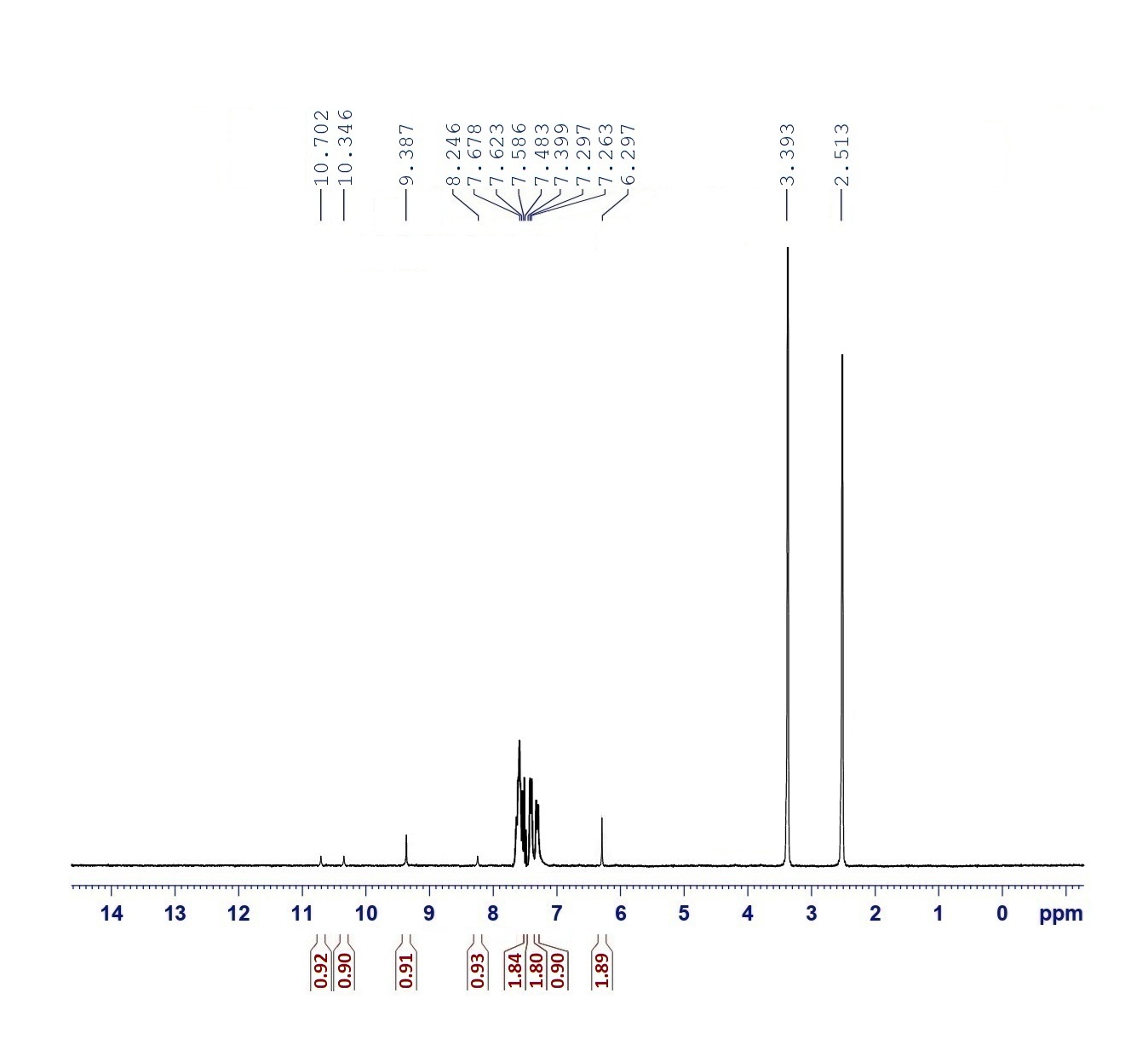
**

**Figure S31.** ^1^H-NMR of compound **16**


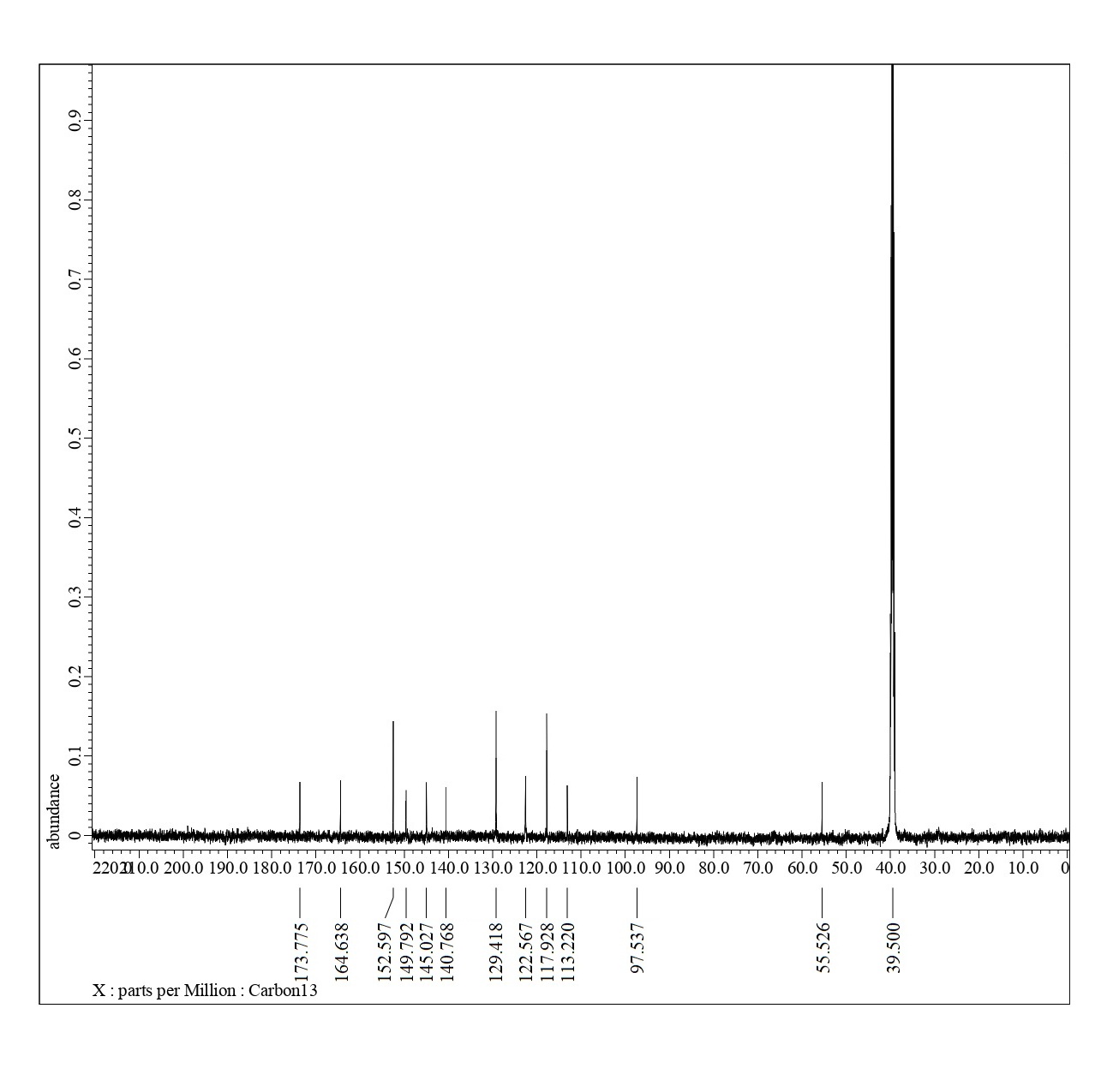

**Figure S32.** ^13^C-NMR of compound **16**

**
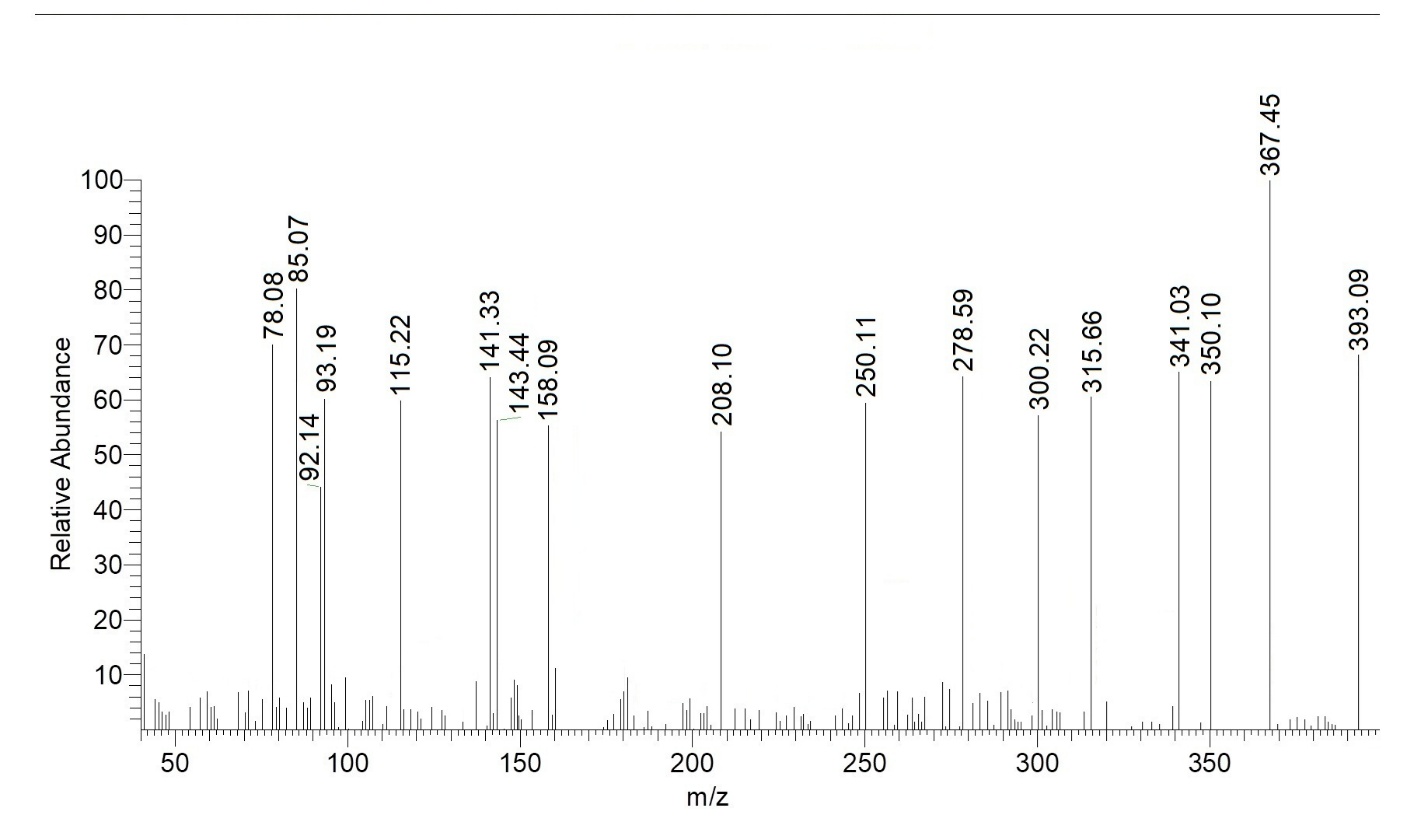
**

**Figure S33.** Mass spectrum of compound **16**

**
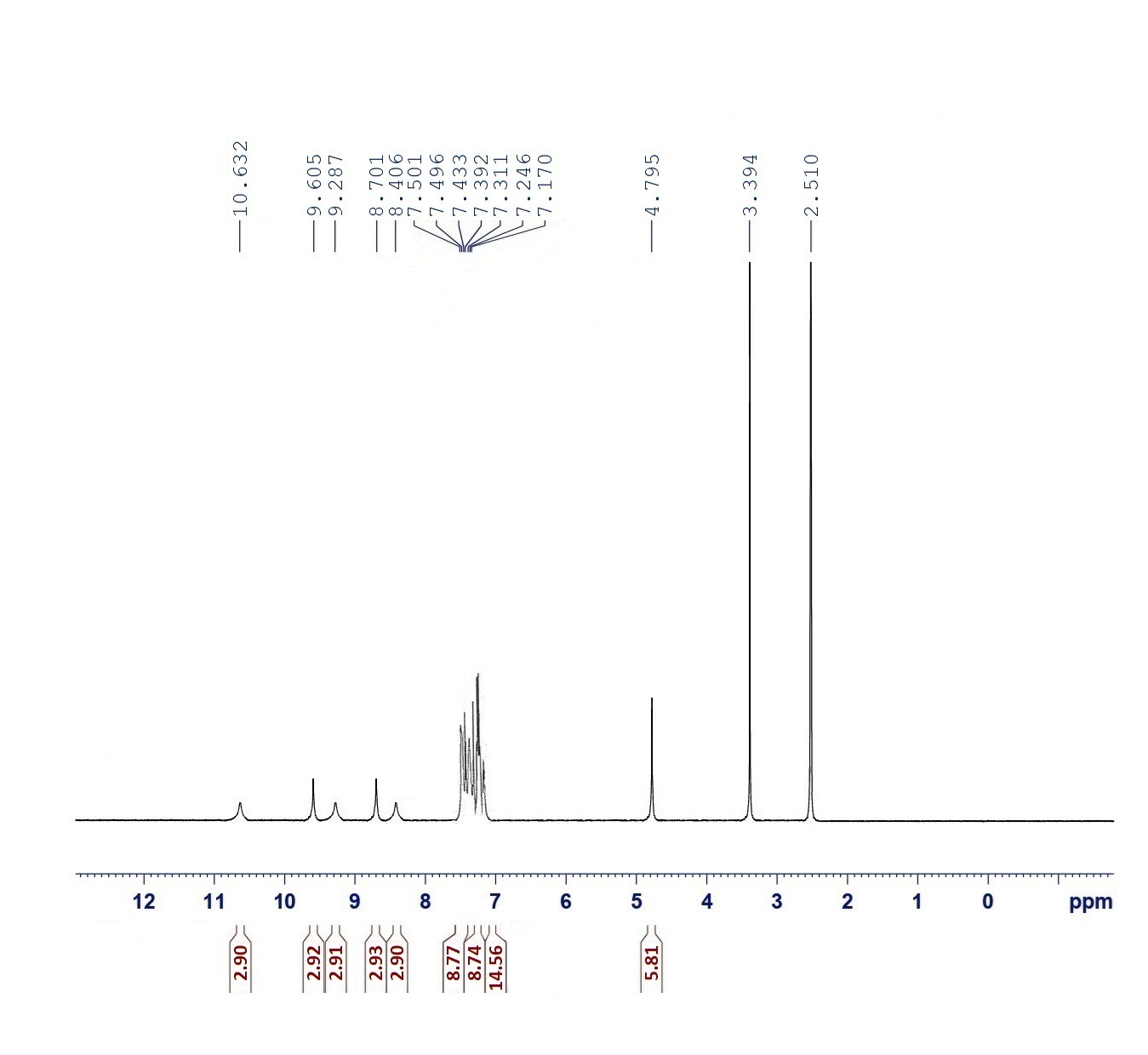
**

**Figure S34.** ^1^H-NMR of compound **17**


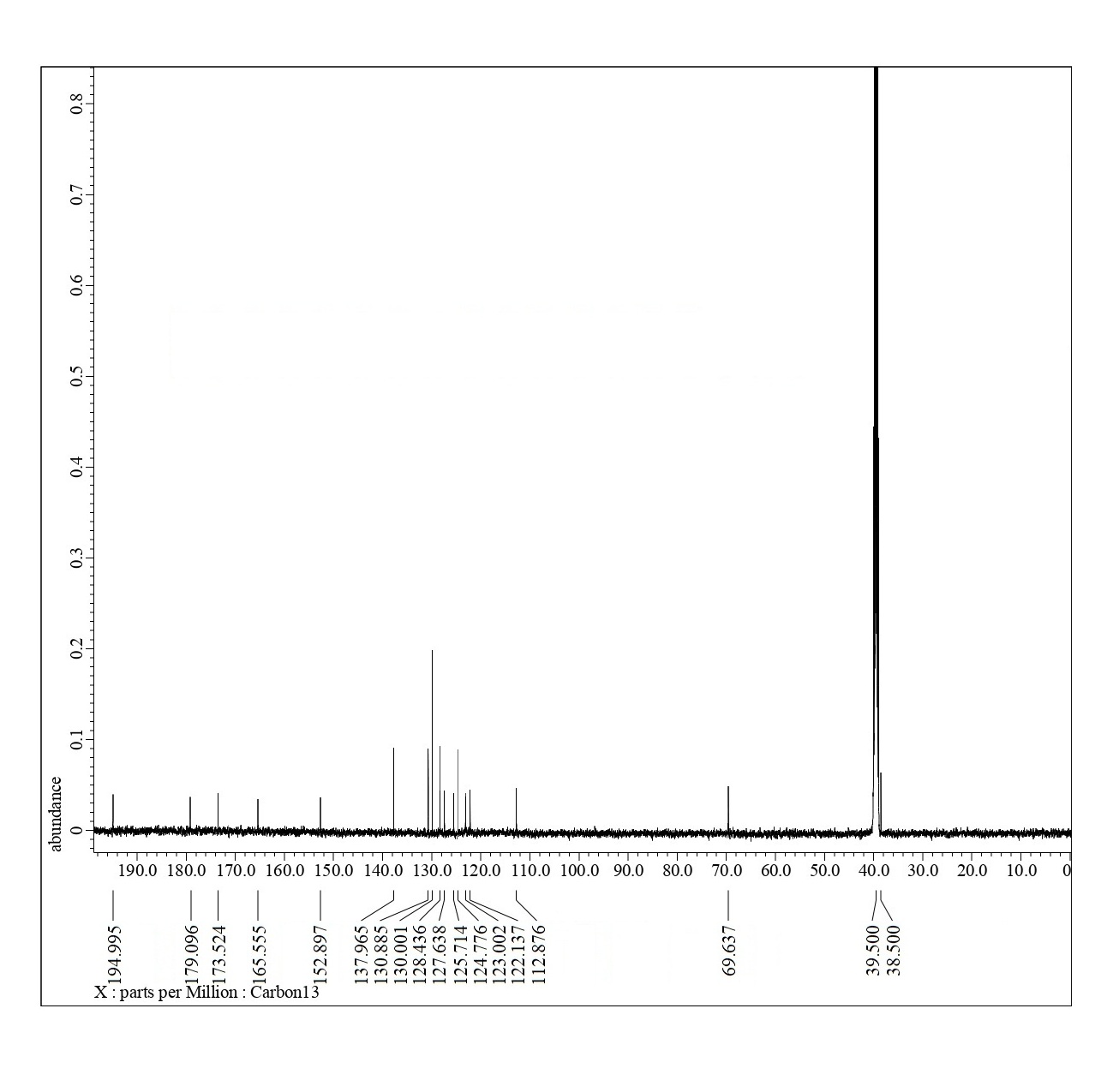

**Figure S35.** ^13^C-NMR of compound **17**

**
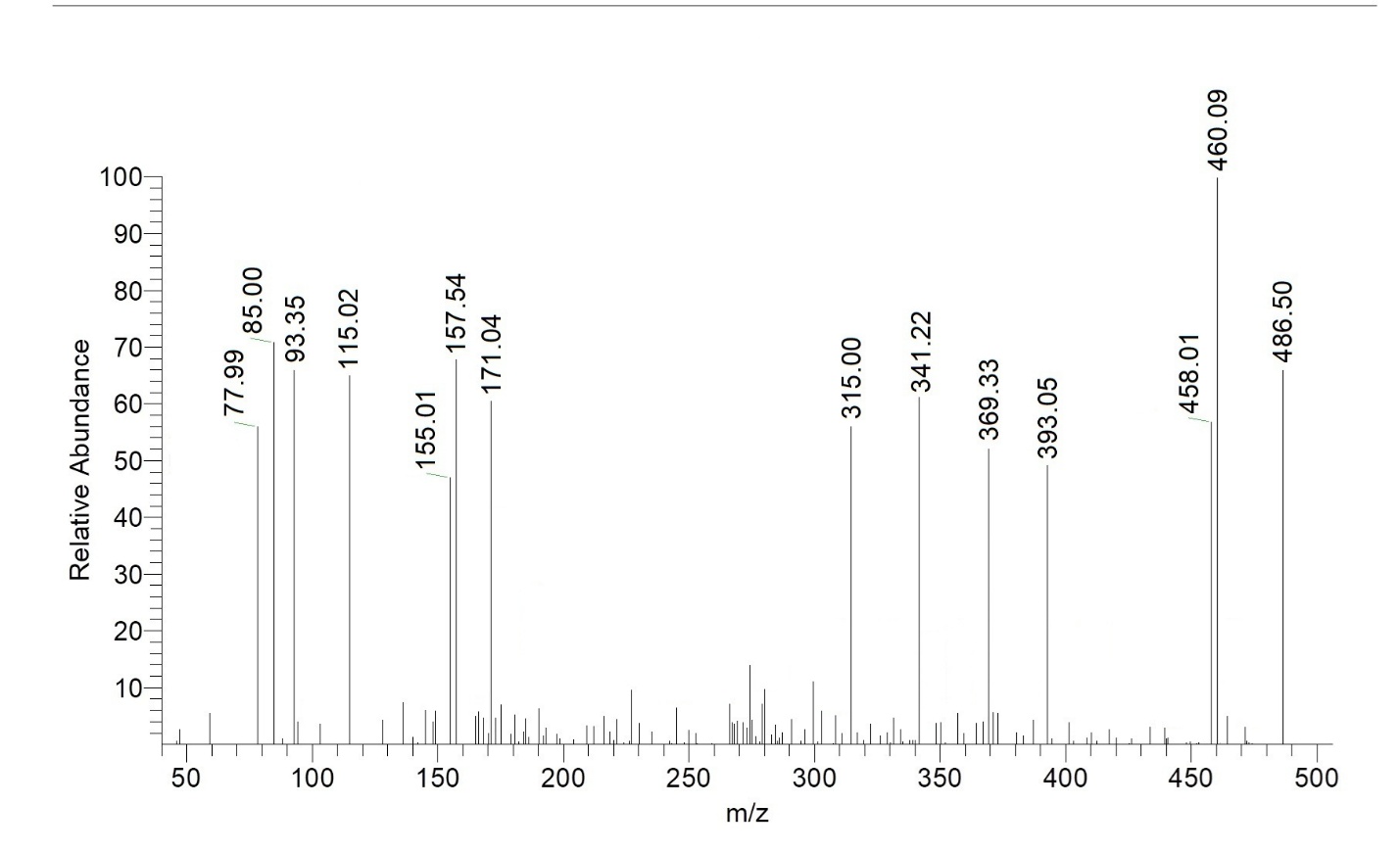
**

**Figure S36.** Mass spectrum of compound **17**

**
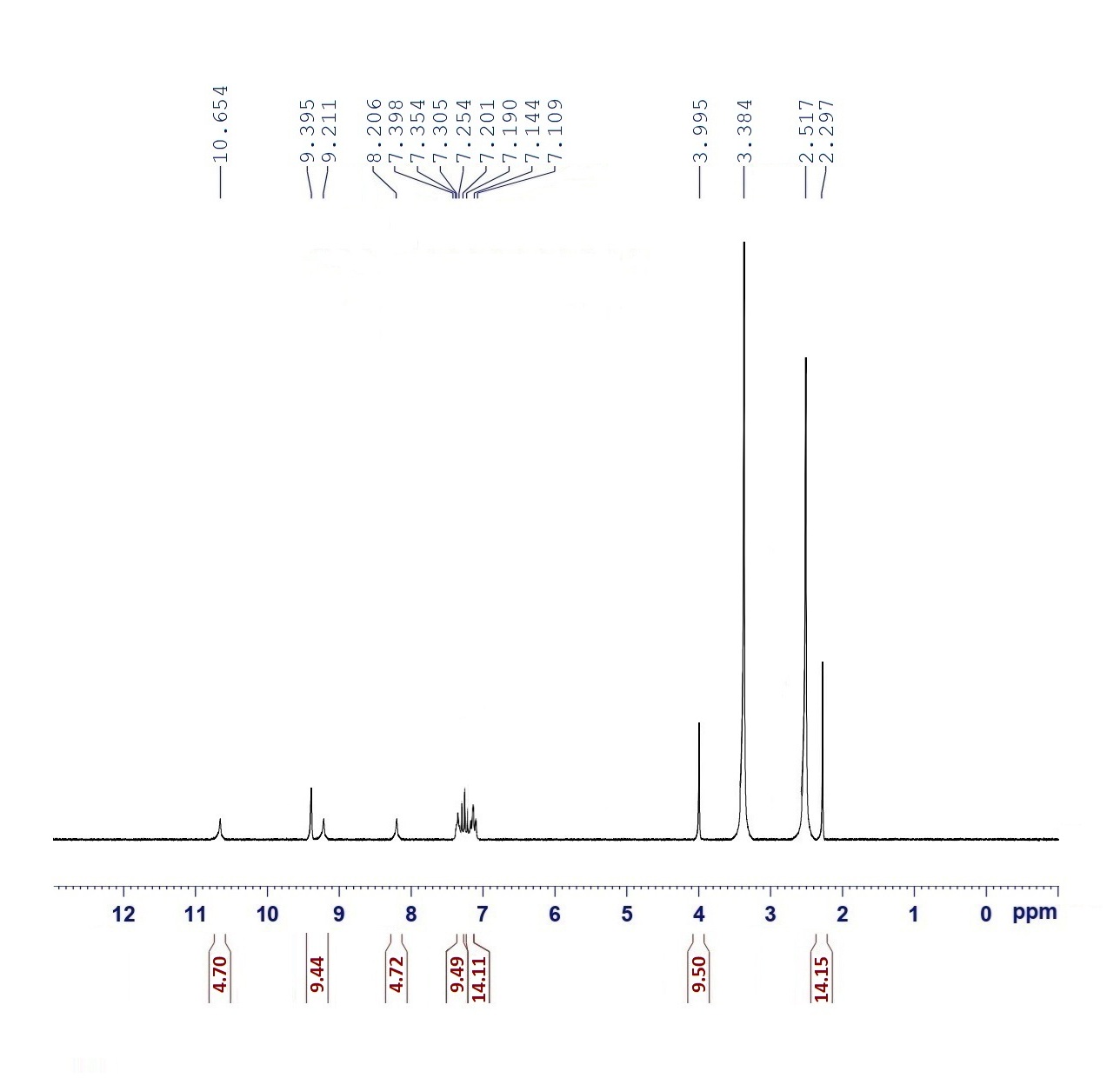
**

**Figure S37.** ^1^H-NMR of compound **19**


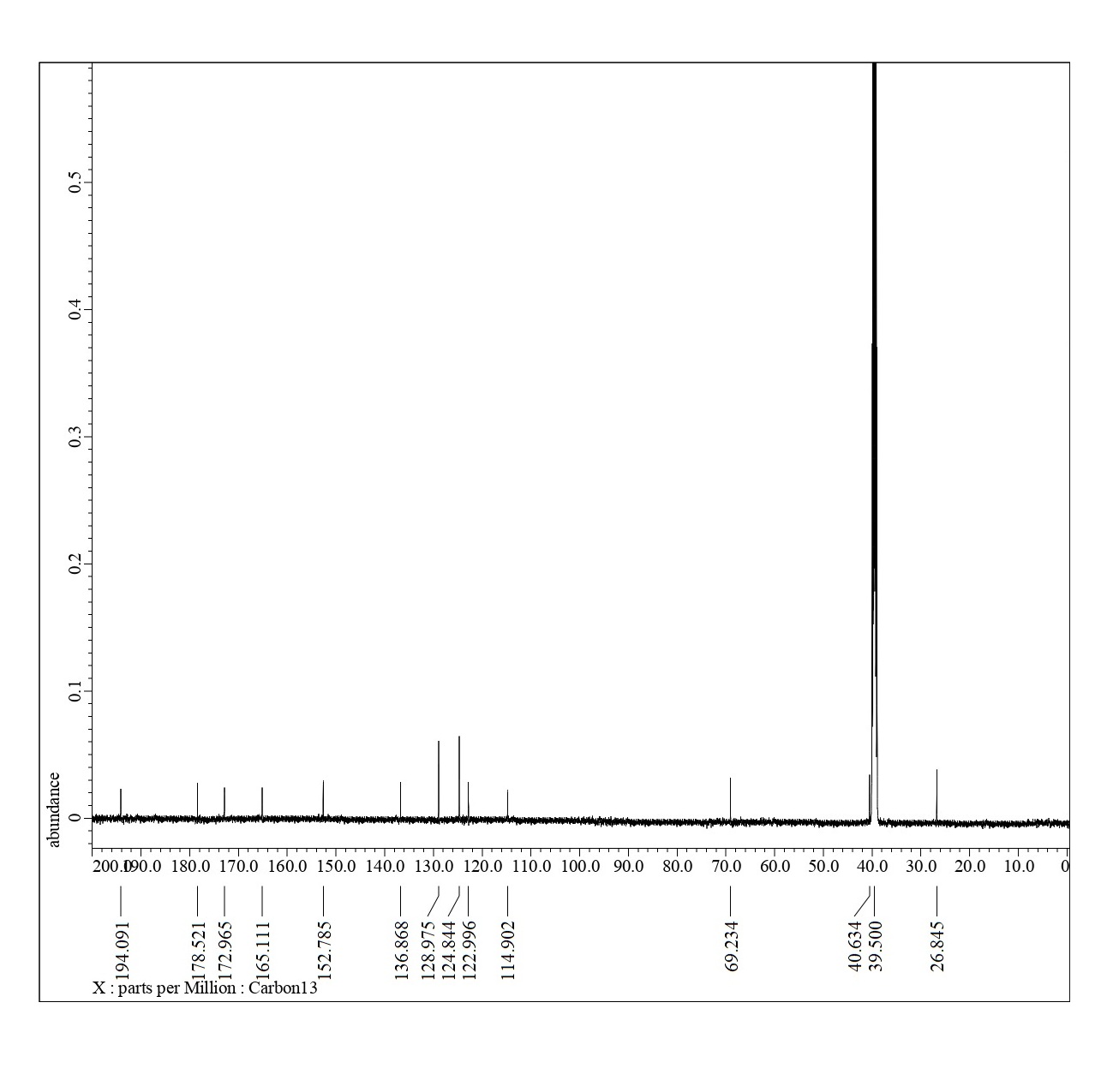

**Figure S38.** ^13^C-NMR of compound **19**

**
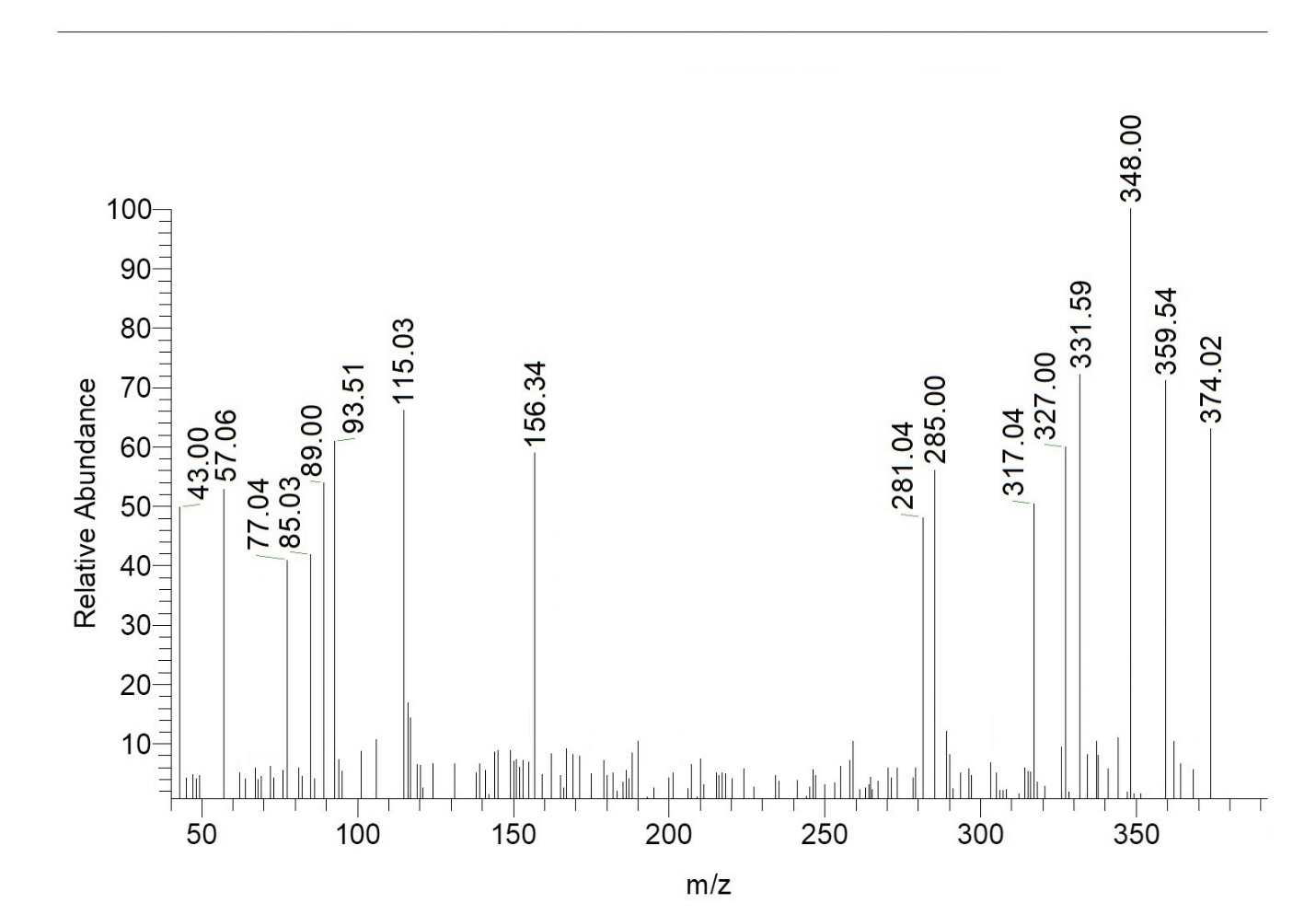
**

**Figure S39.** Mass spectrum of compound **19**

**
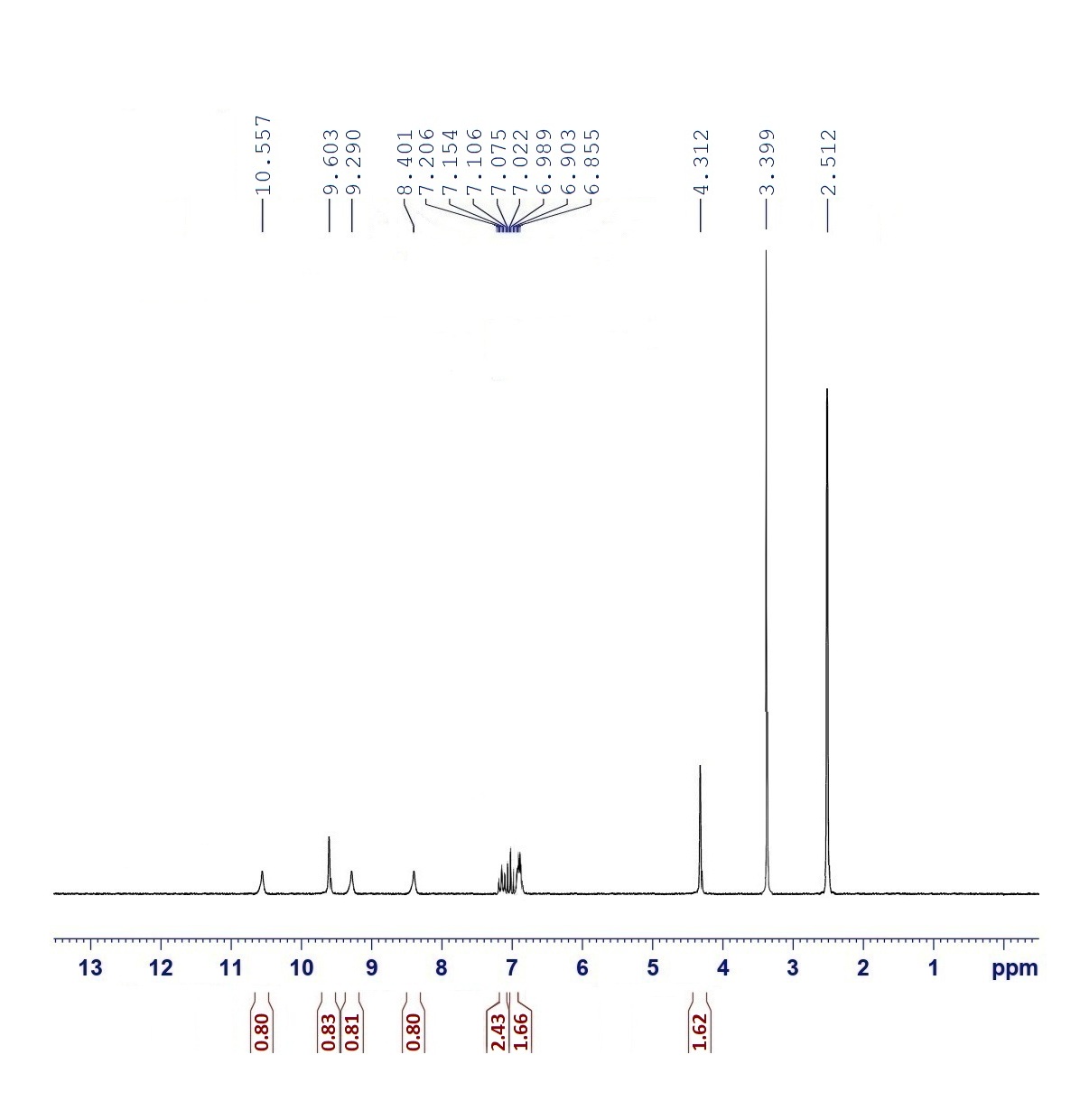
**

**Figure S40.** ^1^H-NMR of compound **21**


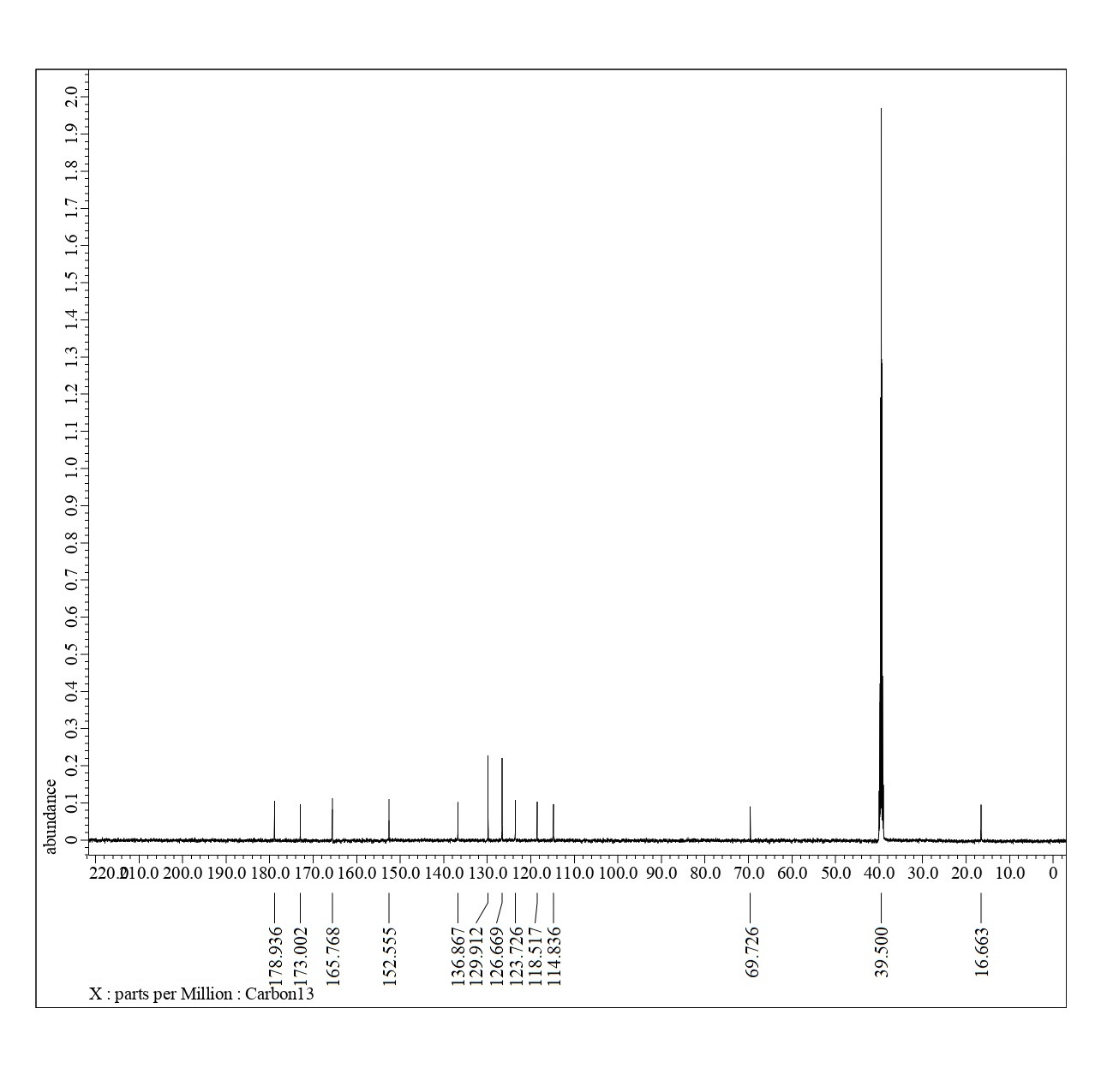

**Figure S41.** ^13^C-NMR of compound **21**

**
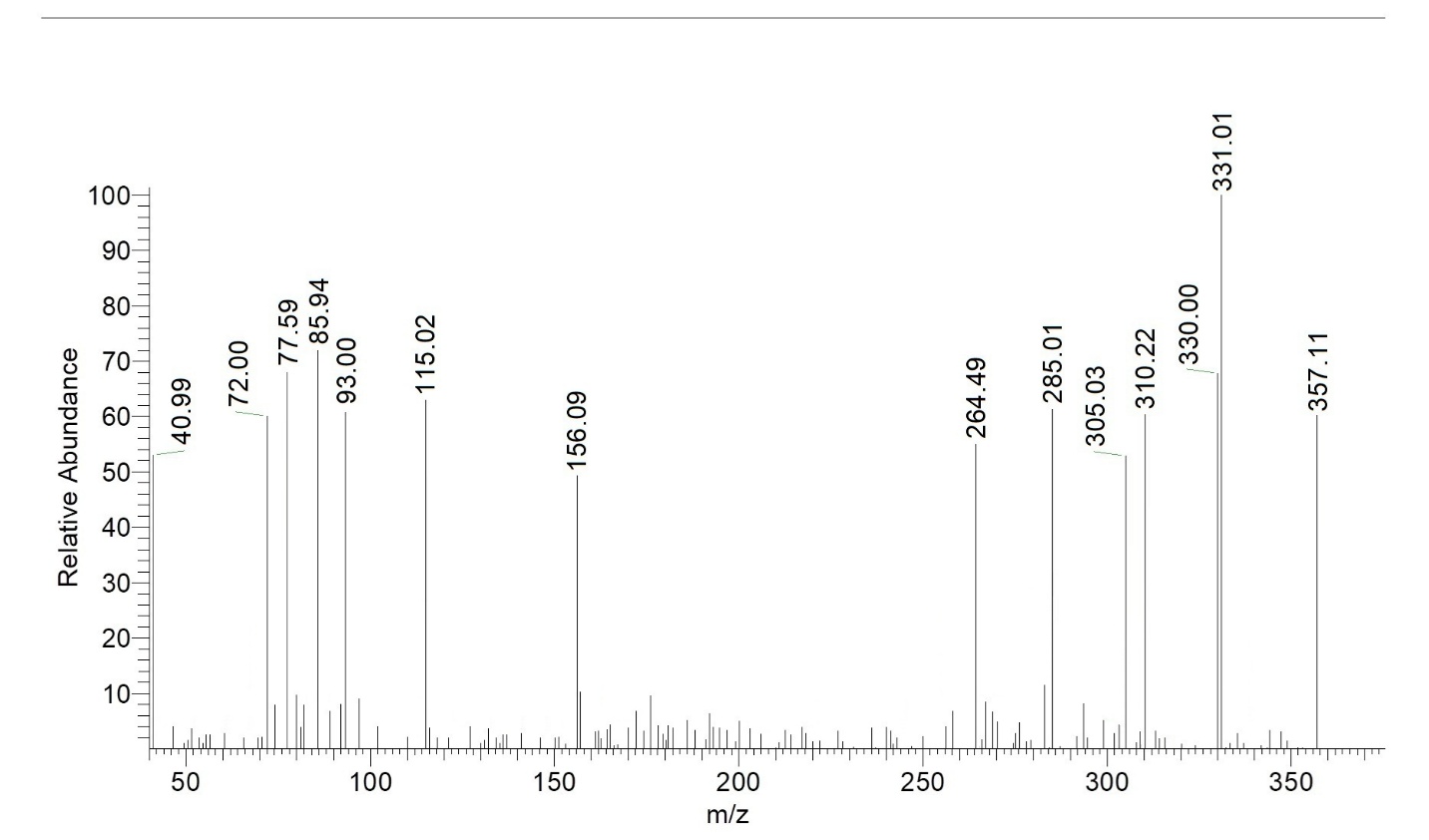
**

**Figure S42.** Mass spectrum of compound **21**

**
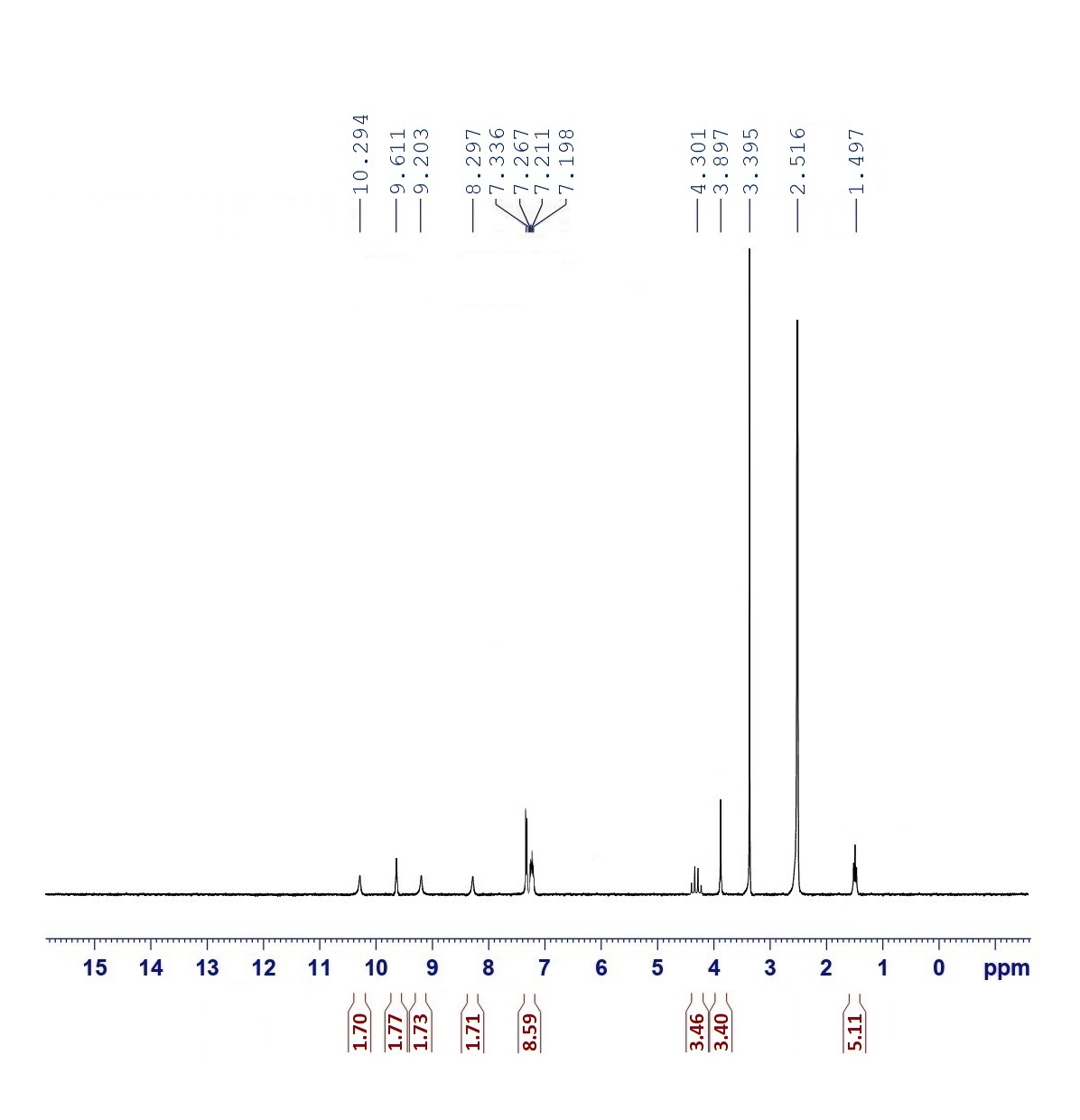
**

**Figure S43.** ^1^H-NMR of compound **23**


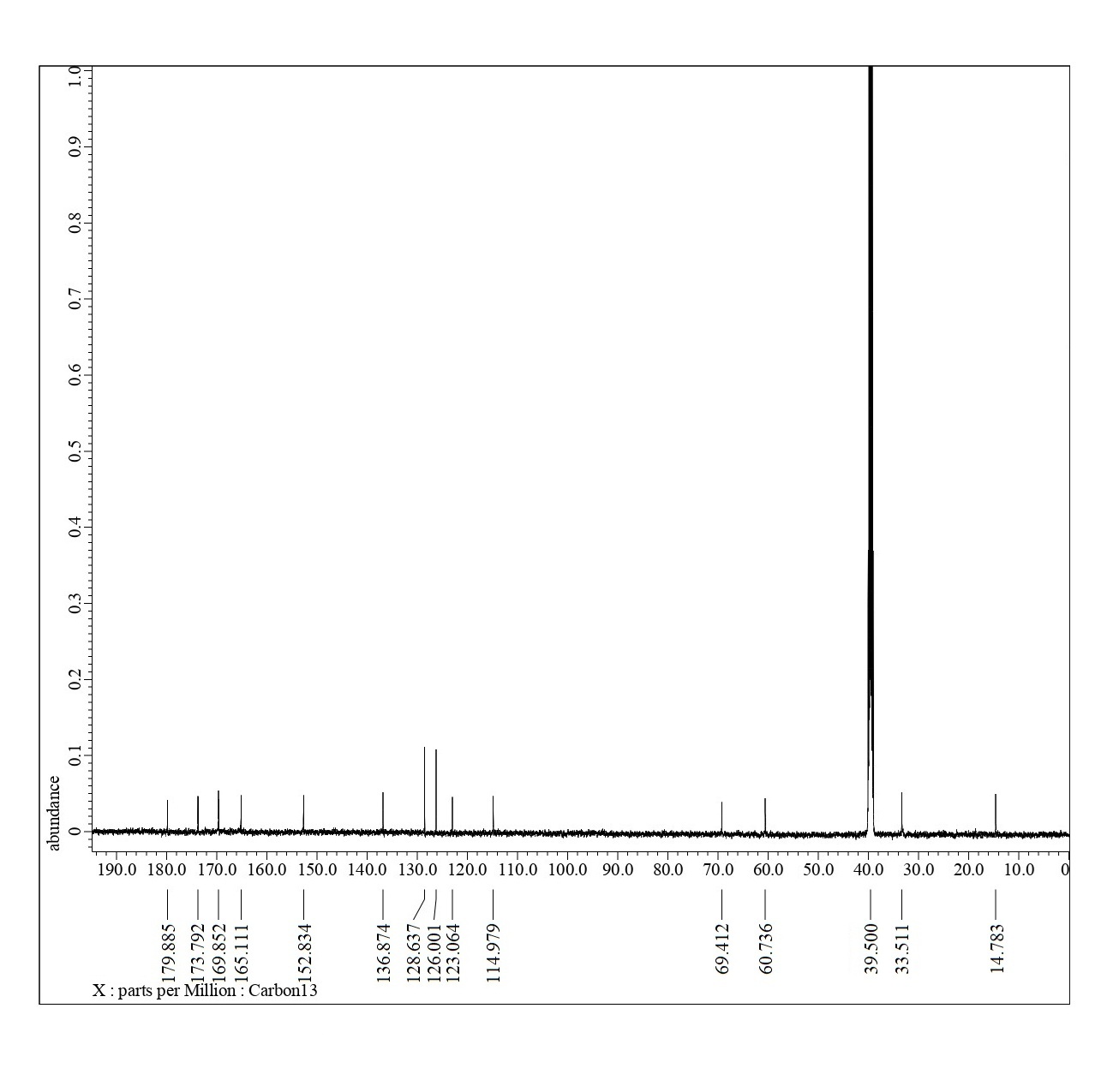

**Figure S44.** ^13^C-NMR of compound **23**

**
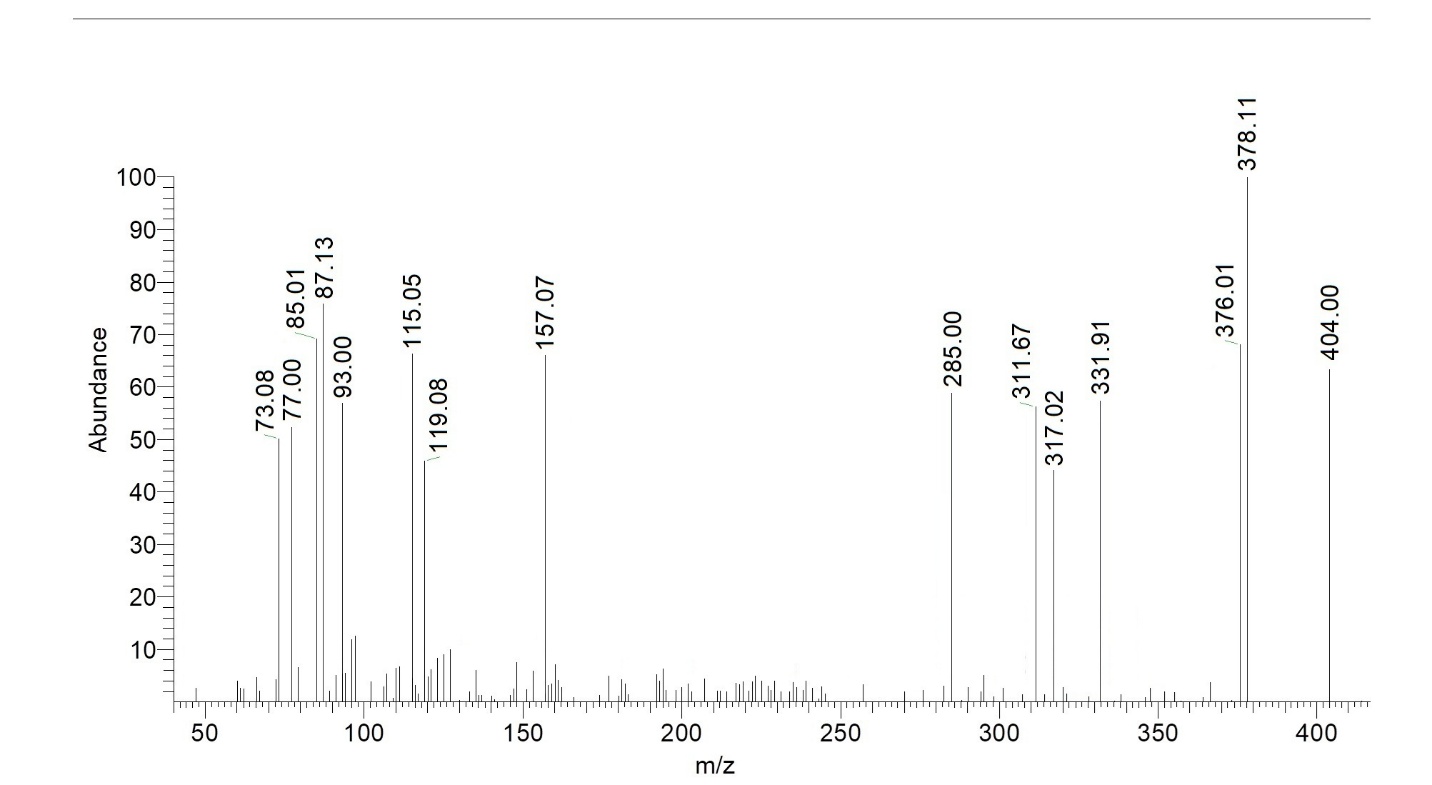
**

**Figure S45.** Mass spectrum of compound **23**


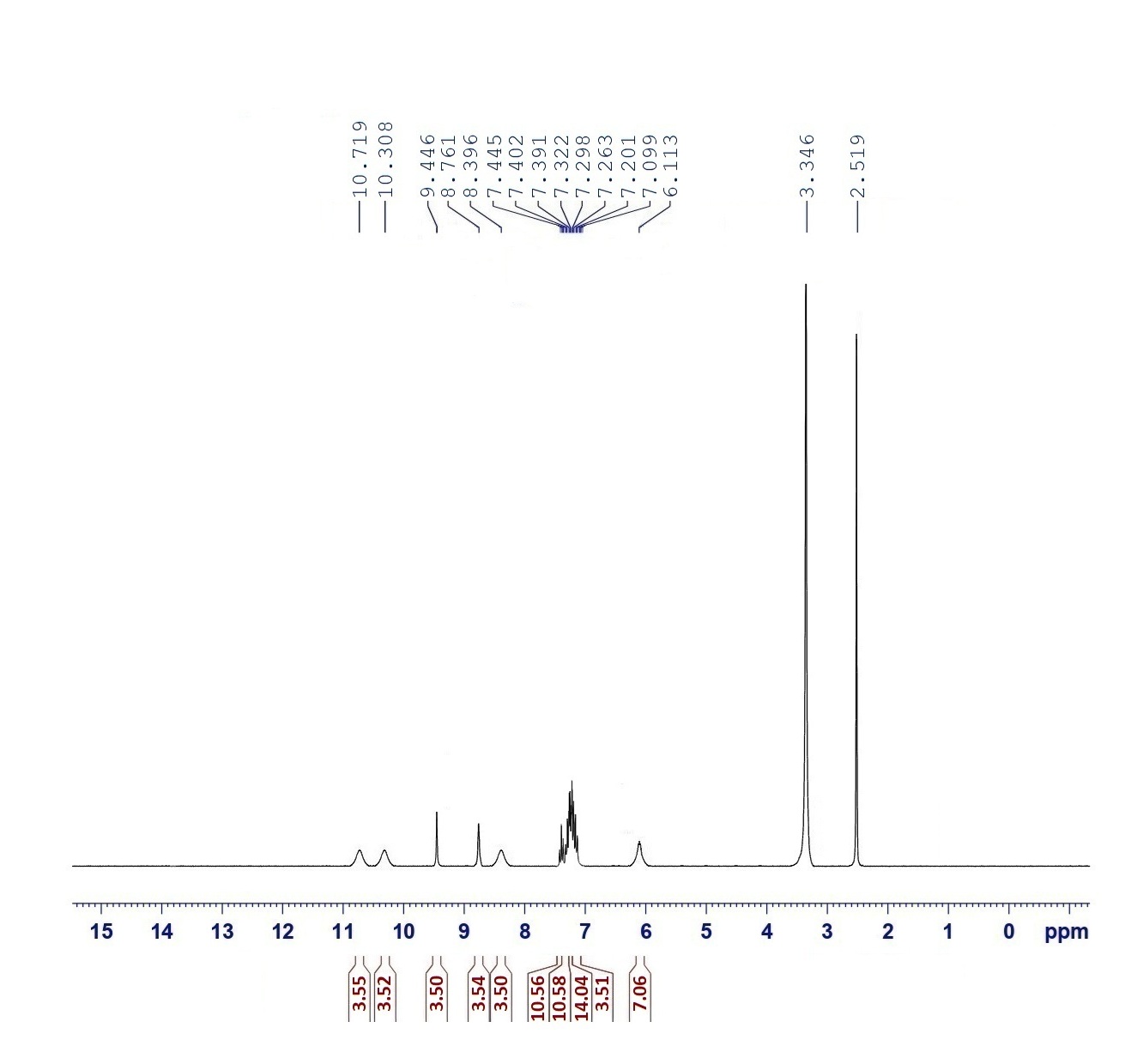

**Figure S46.** ^1^H-NMR of compound **18**

**
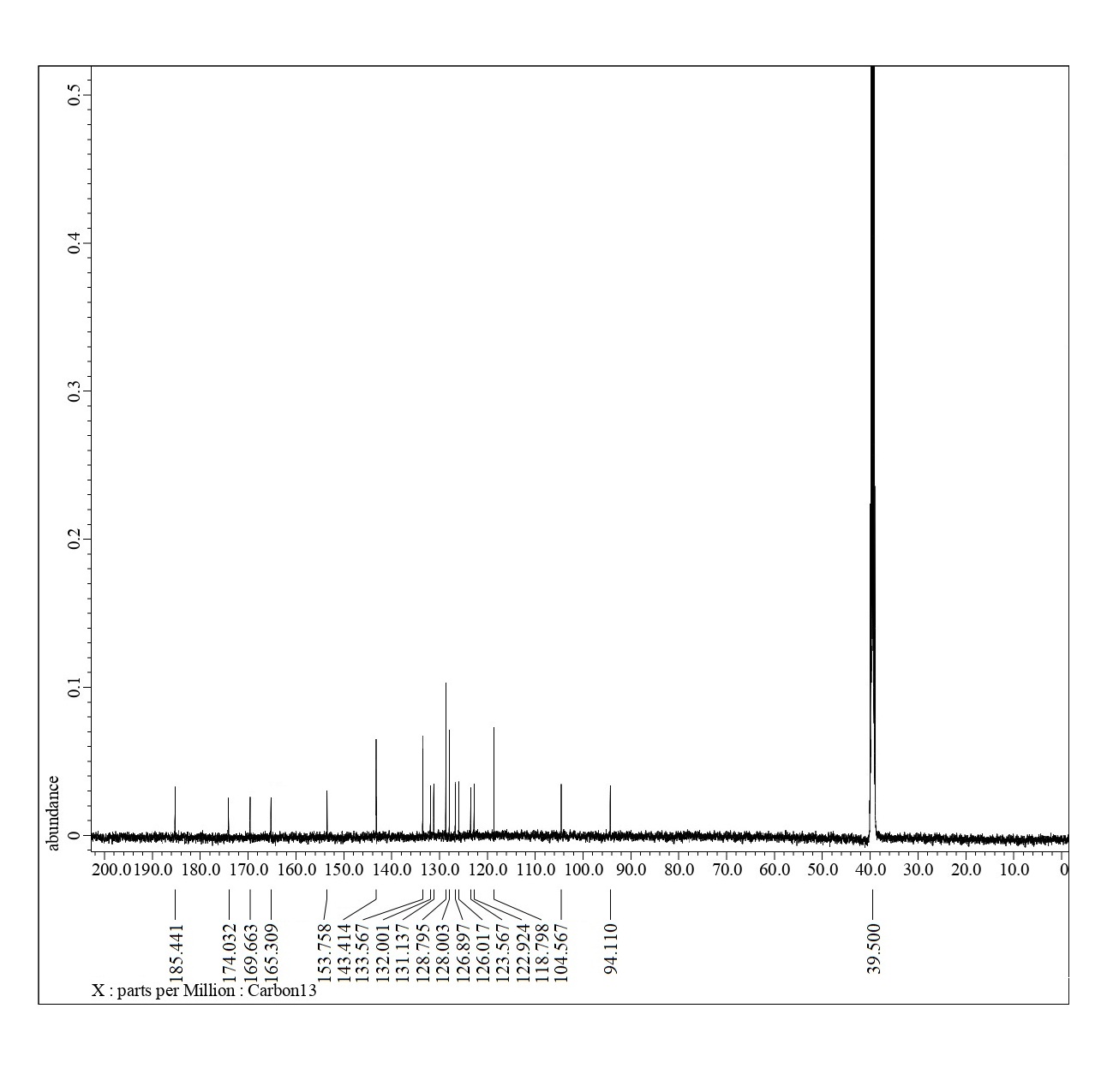
**

**Figure S47.** ^13^C-NMR of compound **18**

**
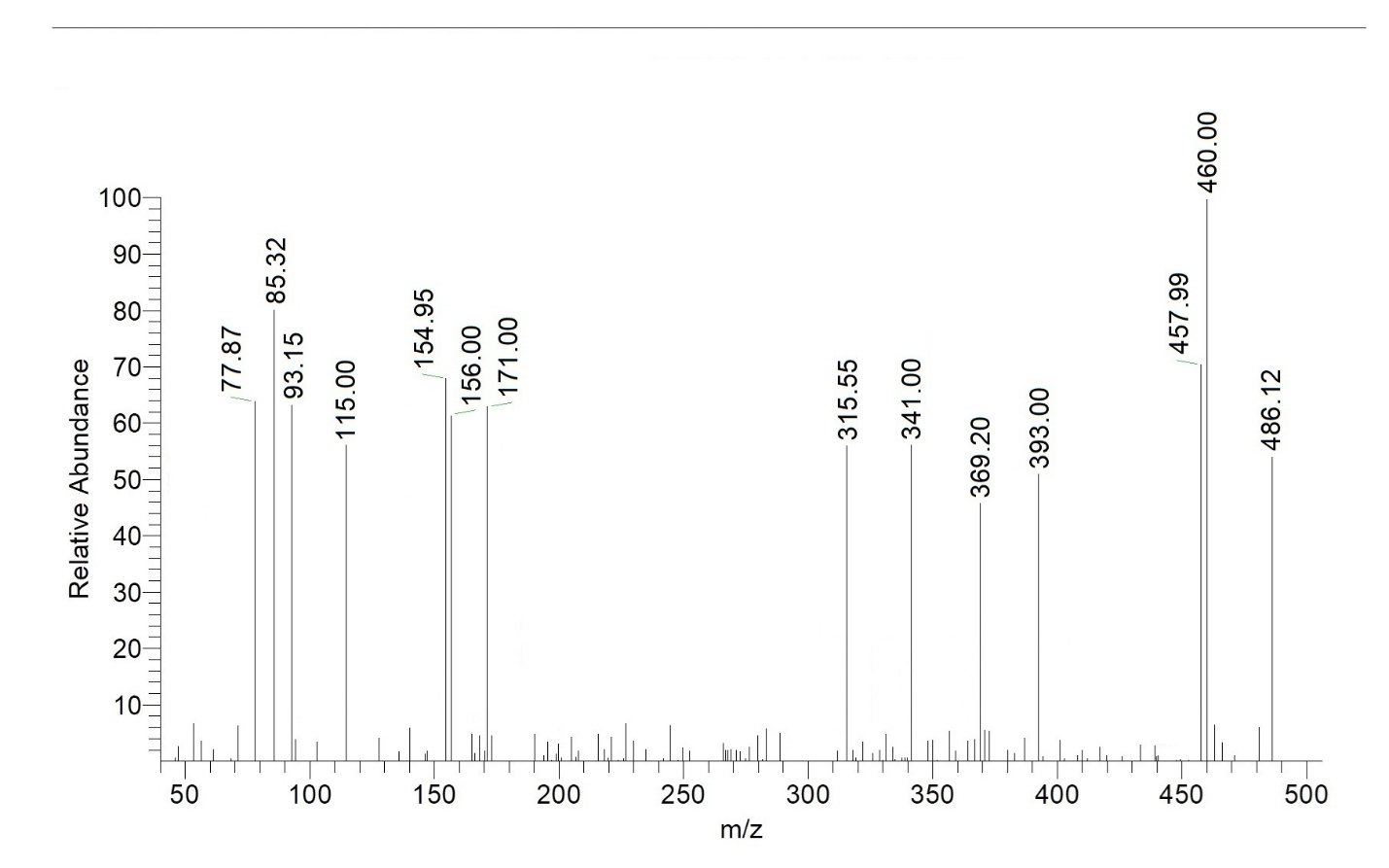
**

**Figure S48.** Mass spectrum of compound **18**

**
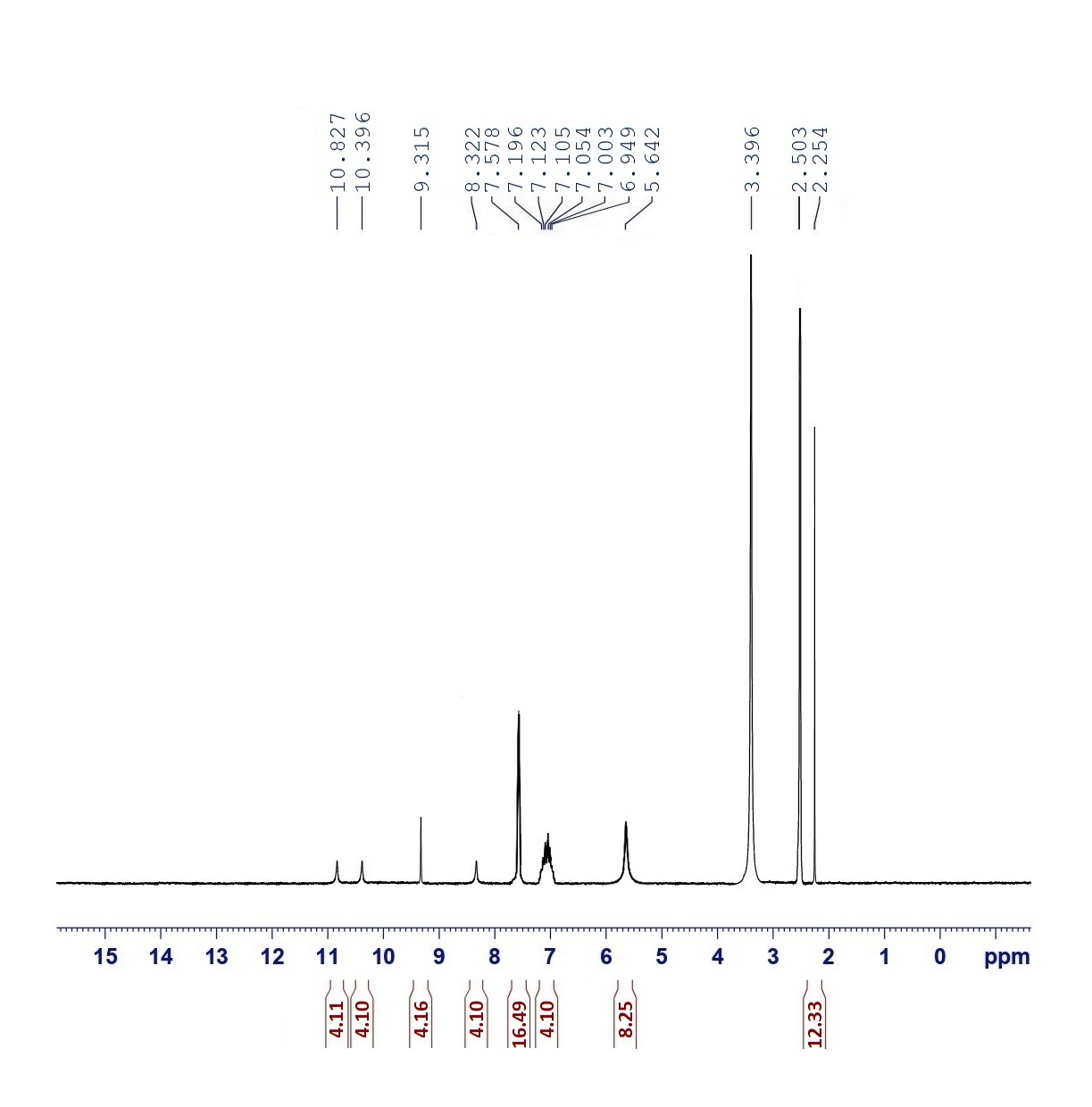
**

**Figure S49.** ^1^H-NMR of compound **20**

**
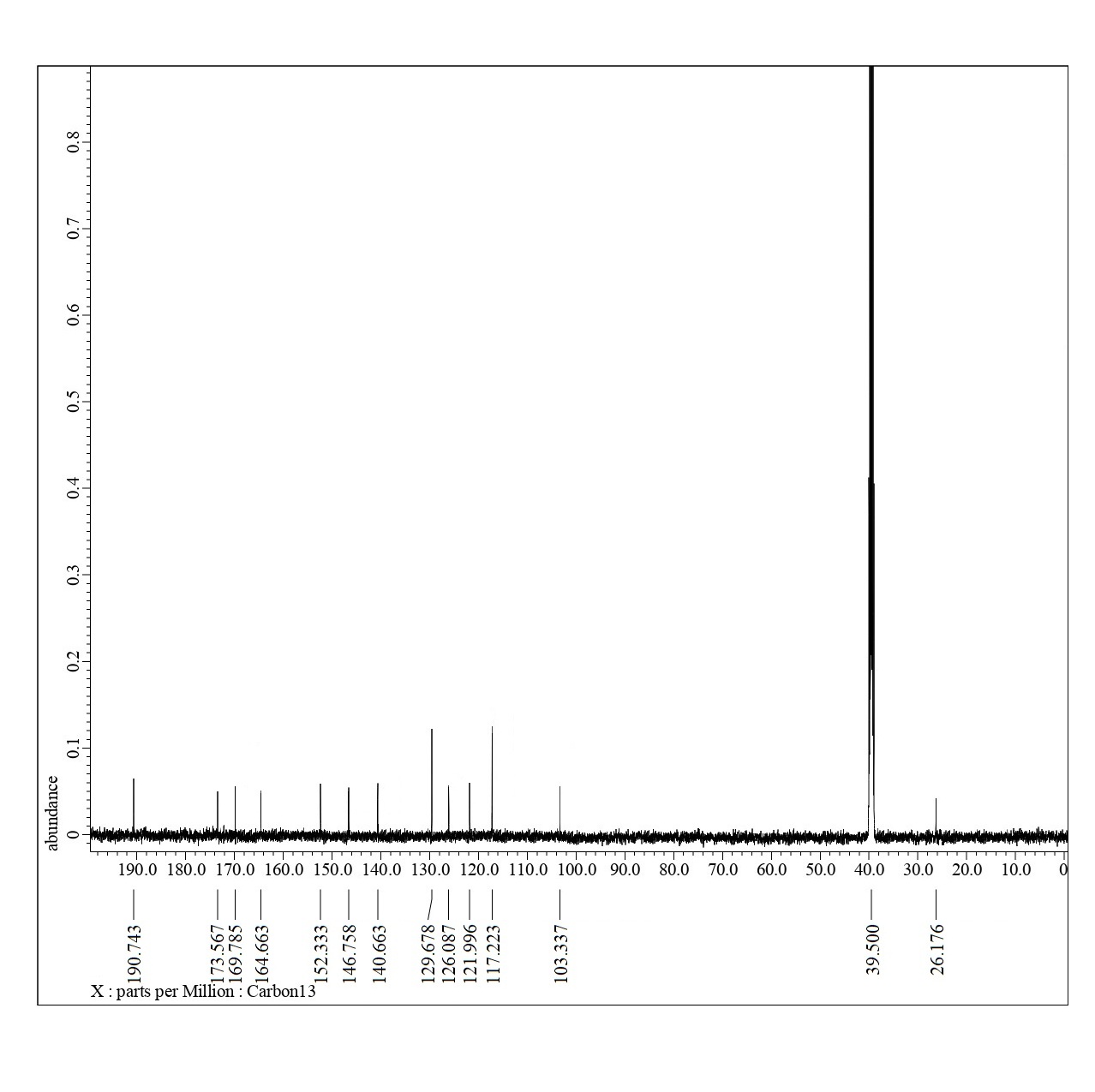
**

**Figure S50.** ^13^C-NMR of compound **20**

**
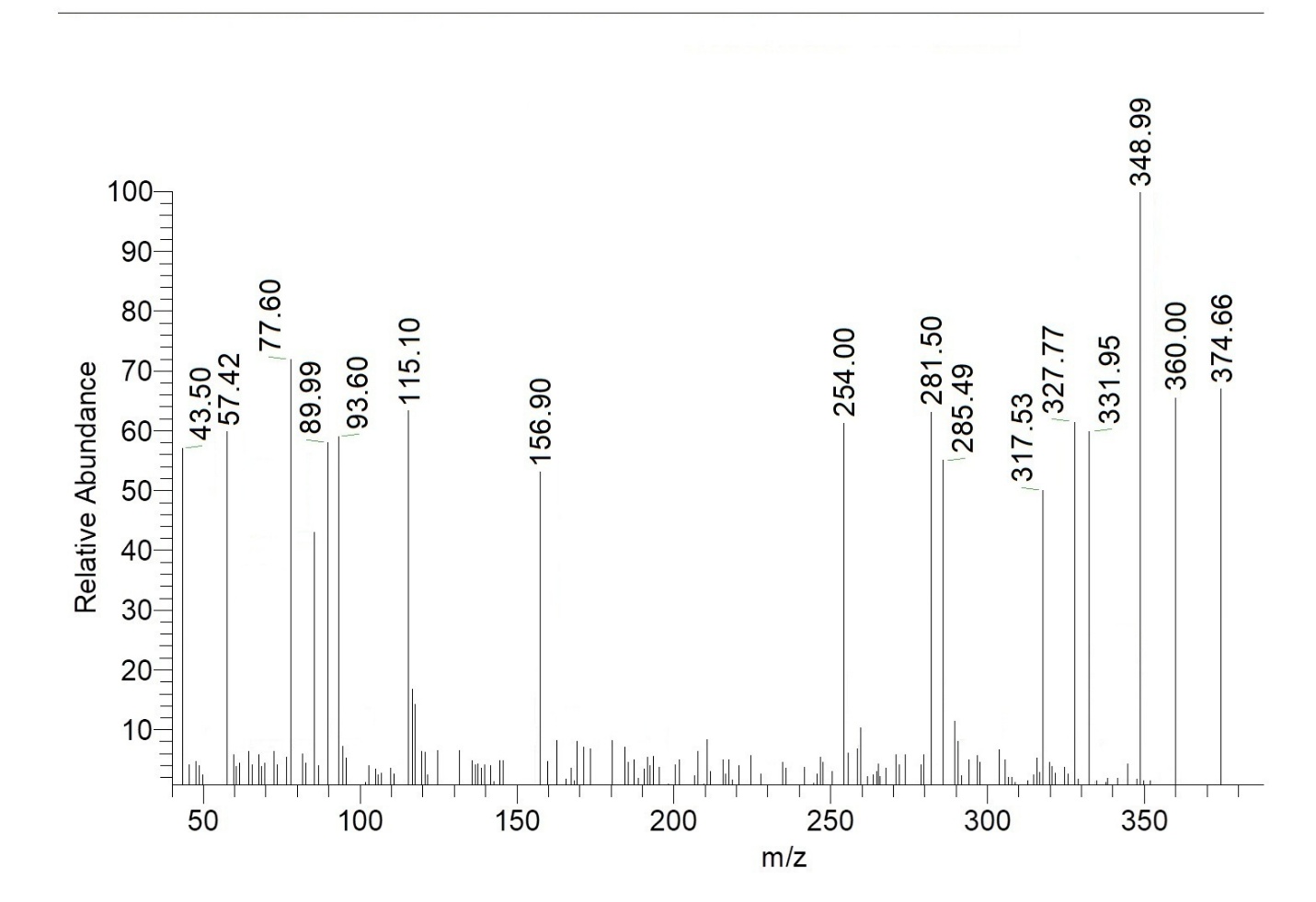
**

**Figure S51.** Mass spectrum of compound **20**

**
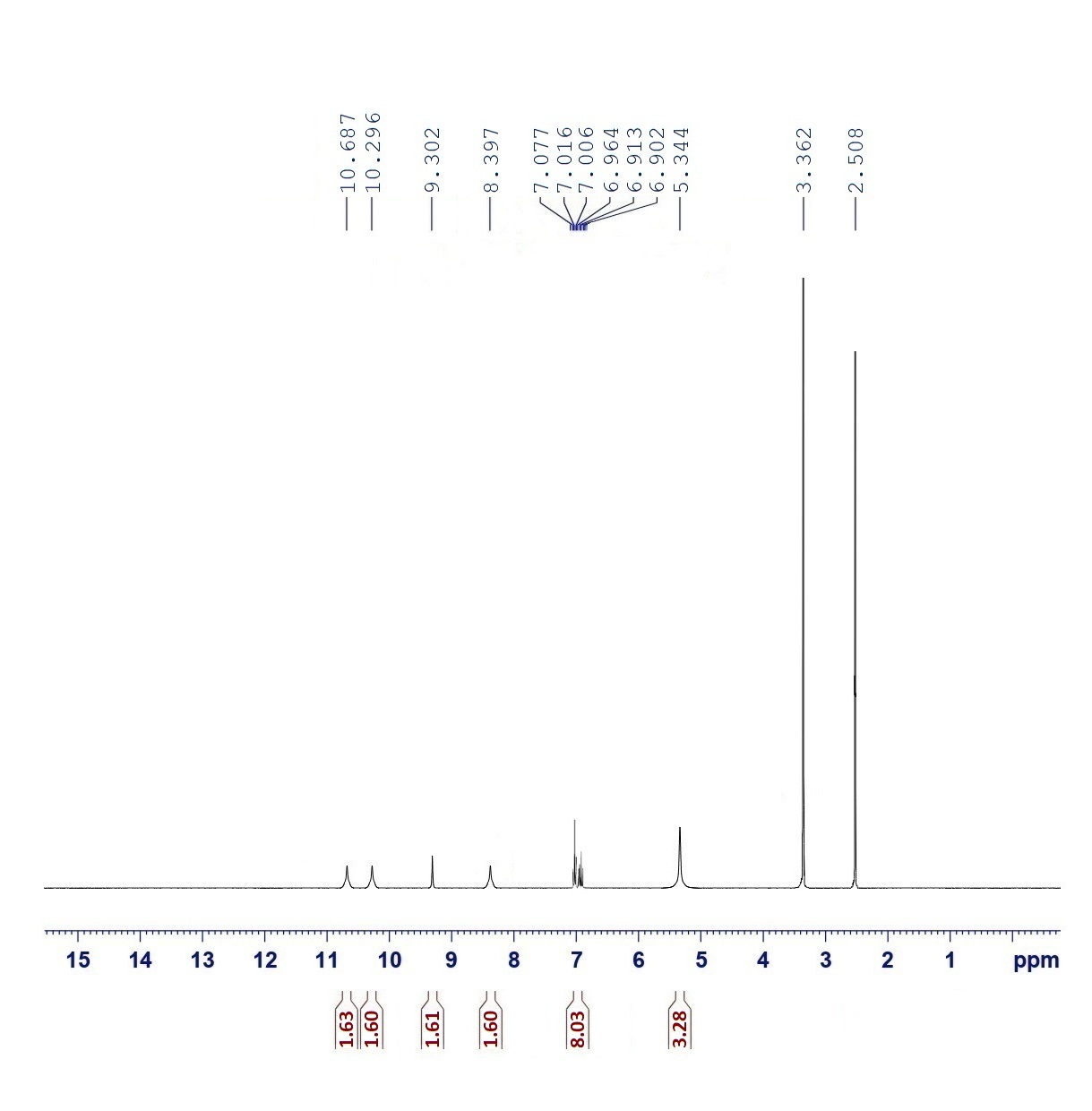
**

**Figure S52.** ^1^H-NMR of compound **22**

**
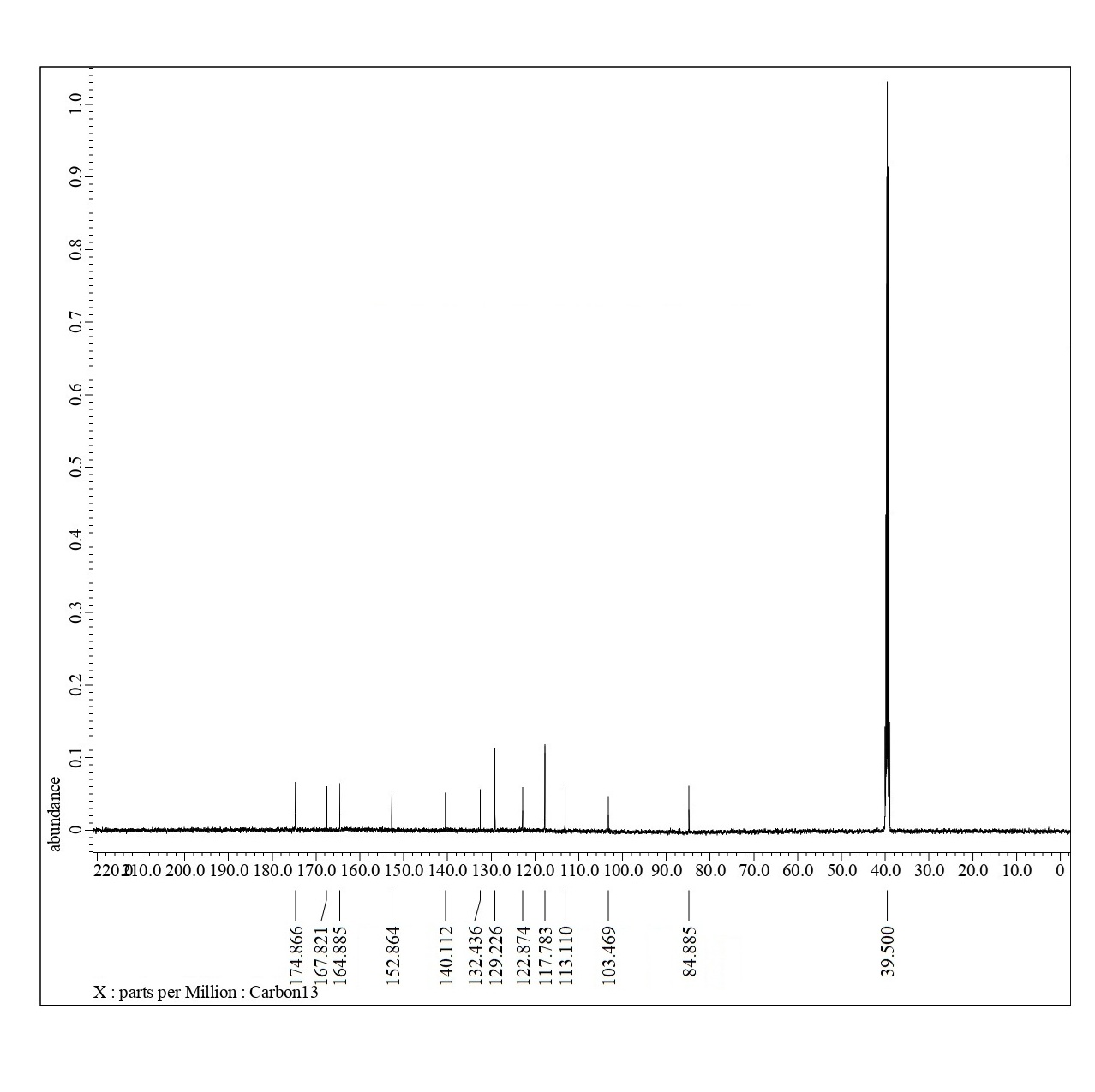
**

**Figure S53.** ^13^C-NMR of compound **22**

**
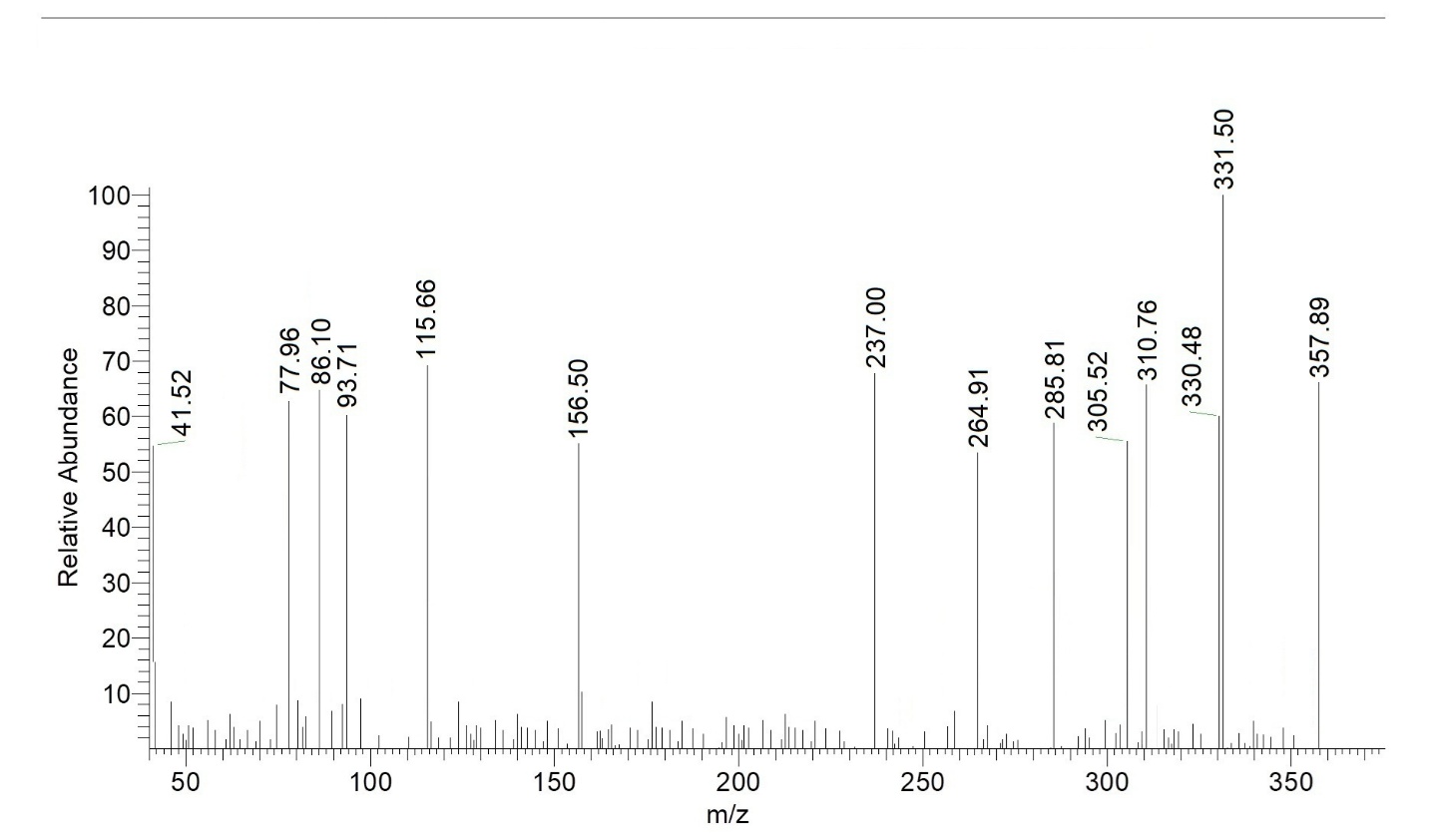
**

**Figure S54.** Mass spectrum of compound **22**

**
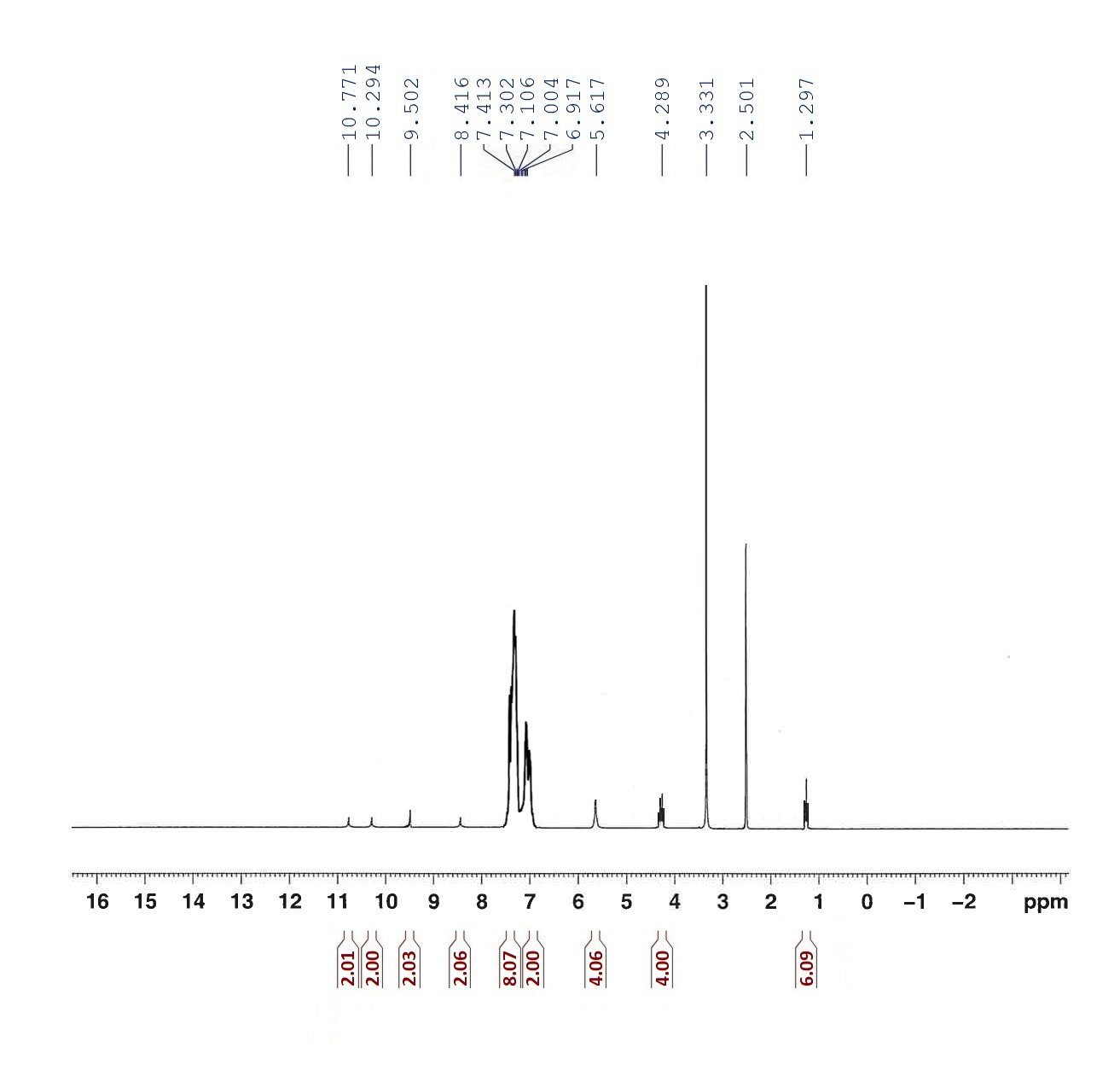
**

**Figure S55.** ^1^H-NMR of compound **24**

**
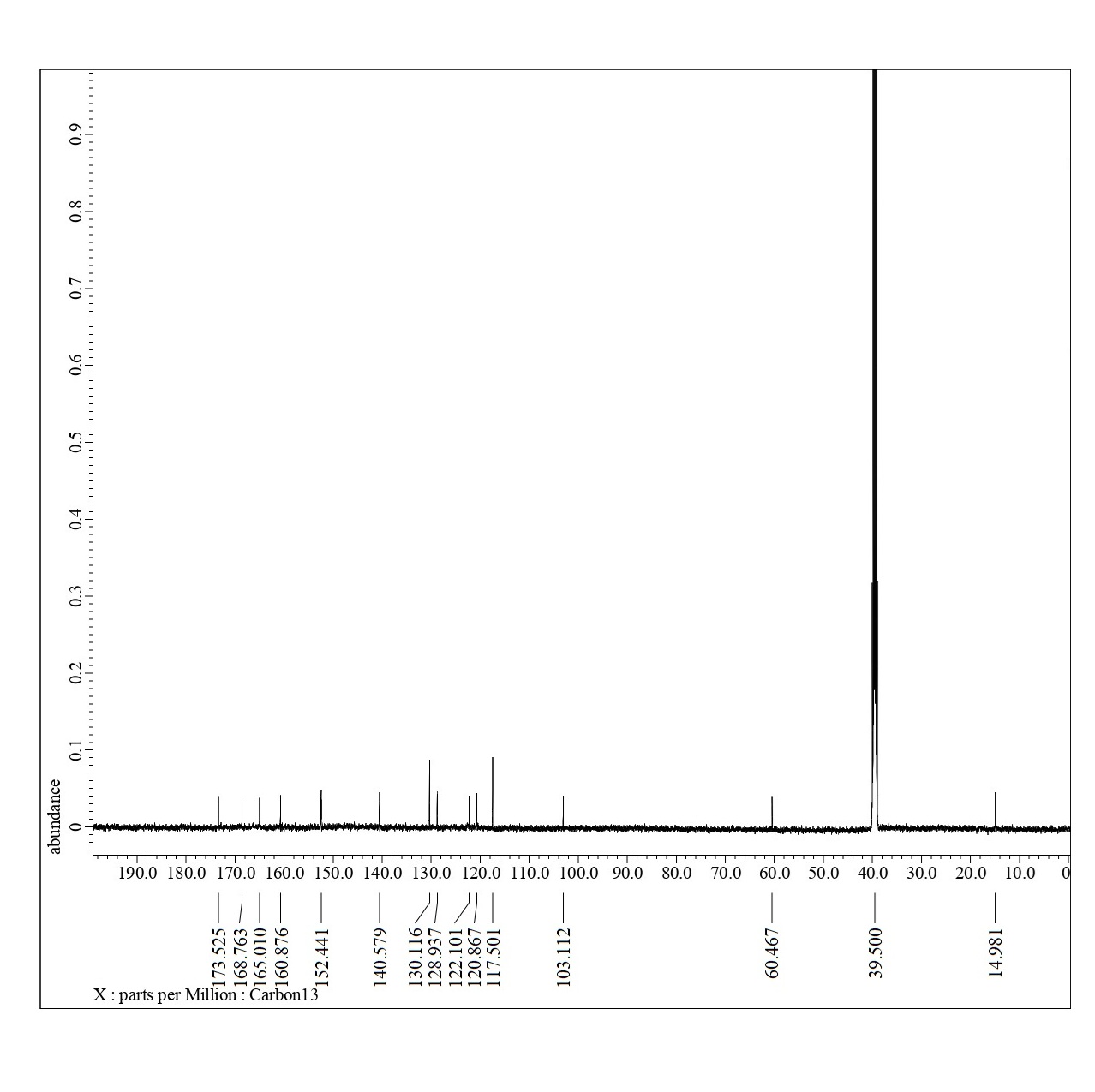
**

**Figure S56.** ^13^C-NMR of compound **24**

**
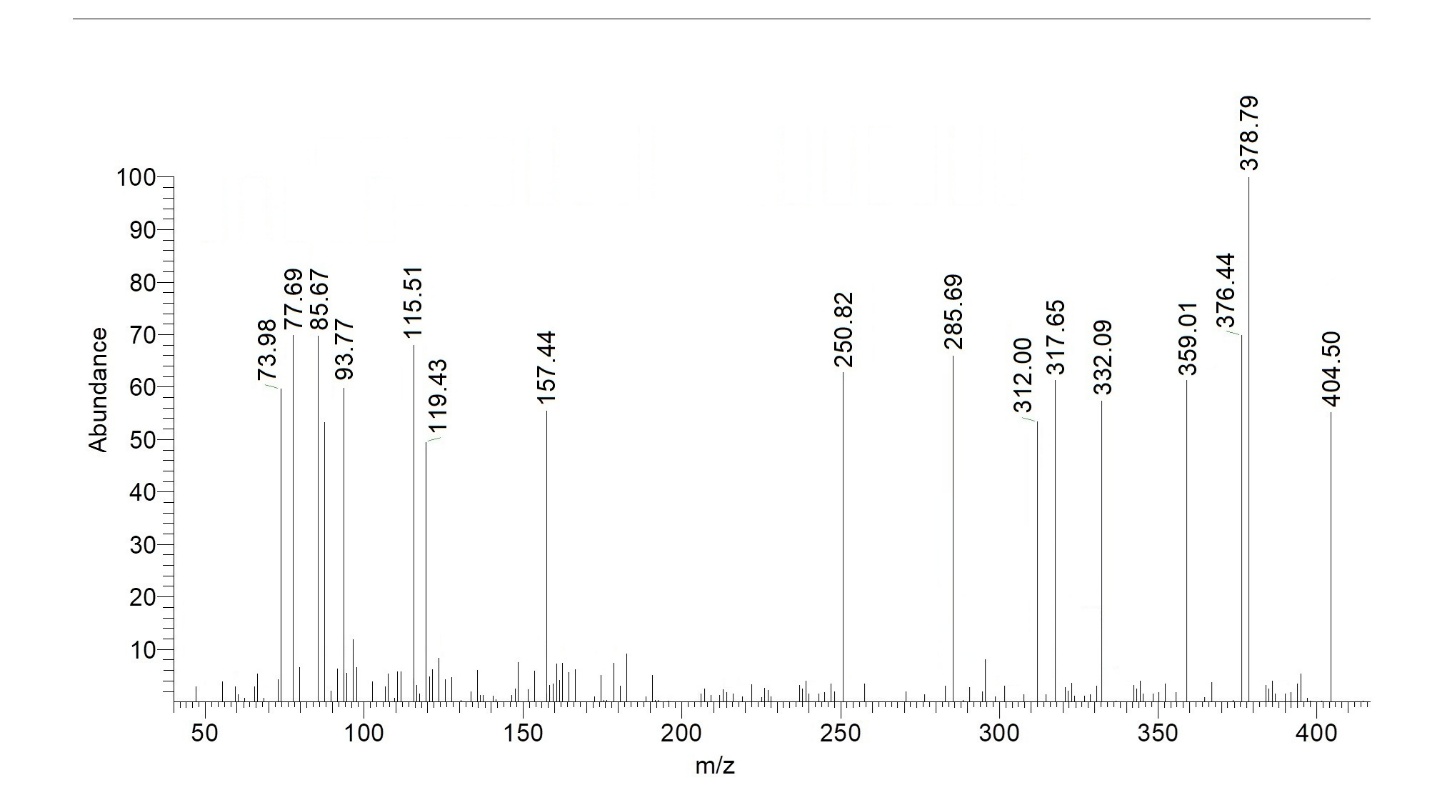
**

**Figure S57.** Mass spectrum of compound **24**
